# Pervasive effects of a dominant foliar endophytic fungus on host genetic and phenotypic expression in a tropical tree

**DOI:** 10.3389/fmicb.2014.00479

**Published:** 2014-09-12

**Authors:** Luis C. Mejía, Edward A. Herre, Jed P. Sparks, Klaus Winter, Milton N. García, Sunshine A. Van Bael, Joseph Stitt, Zi Shi, Yufan Zhang, Mark J. Guiltinan, Siela N. Maximova

**Affiliations:** ^1^Smithsonian Tropical Research InstituteUnit 9100, USA; ^2^Department of Plant Science and The Huck Institutes of the Life Sciences, The Pennsylvania State UniversityUniversity Park, PA, USA; ^3^Department of Ecology and Evolution, Cornell UniversityIthaca, NY, USA; ^4^Department of Ecology and Evolutionary Biology, Tulane UniversityNew Orleans, LA, USA; ^5^Social, Life and Engineering Sciences Imaging Center, Materials Research InstituteUniversity Park, PA, USA

**Keywords:** symbiosis, fungal endophytes, *Theobroma*, *Colletotrichum*, gene expression, plant defense, *Arabidopsis*, *Populus*

## Abstract

It is increasingly recognized that macro-organisms (corals, insects, plants, vertebrates) consist of both host tissues and multiple microbial symbionts that play essential roles in their host's ecological and evolutionary success. Consequently, identifying benefits and costs of symbioses, as well as mechanisms underlying them are research priorities. All plants surveyed under natural conditions harbor foliar endophytic fungi (FEF) in their leaf tissues, often at high densities. Despite producing no visible effects on their hosts, experiments have nonetheless shown that FEF reduce pathogen and herbivore damage. Here, combining results from three genomic, and two physiological experiments, we demonstrate pervasive genetic and phenotypic effects of the apparently asymptomatic endophytes on their hosts. Specifically, inoculation of endophyte-free (E−) *Theobroma cacao* leaves with *Colletotrichum tropicale* (E+), the dominant FEF species in healthy *T. cacao*, induces consistent changes in the expression of hundreds of host genes, including many with known defensive functions. Further, E+ plants exhibited increased lignin and cellulose content, reduced maximum rates of photosynthesis (A_max_), and enrichment of nitrogen-15 and carbon-13 isotopes. These phenotypic changes observed in E+ plants correspond to changes in expression of specific functional genes in related pathways. Moreover, a cacao gene (*Tc00g04254*) highly up-regulated by *C. tropicale* also confers resistance to pathogen damage in the absence of endophytes or their products in host tissues. Thus, the benefits of increased pathogen resistance in E+ plants are derived in part from up-regulation of intrinsic host defense responses, and appear to be offset by potential costs including reduced photosynthesis, altered host nitrogen metabolism, and endophyte heterotrophy of host tissues. Similar effects are likely in most plant-endophyte interactions, and should be recognized in the design and interpretation of genetic and phenotypic studies of plants.

## Introduction

Although non-pathogenic, microbial symbionts are increasingly recognized for their often profoundly beneficial influences on hosts (Van Rhijn and Vanderleyden, 1995; Kimbell et al., 2006; Herre et al., 2007; Li et al., 2008; Stat et al., 2008; Feldhaar, 2011; Engel and Moran, 2013), studies of genetic and physiological expression in plants typically do not control for, and therefore neglect, their effects. Moreover, how symbiont-induced changes in host genetic and physiological expression underlie associated benefits and costs are largely unexplored (Friesen et al., 2011).

The symbioses of woody plants with foliar endophytic fungi (FEF) offer a tractable experimental system in which the effects of endophytes on their host genetic and phenotypic expression can readily be assessed. All plants surveyed under natural conditions harbor FEF. Despite the fact that they are living in high density or abundance within plant tissues, they produce few, if any, visible effects on their hosts (Arnold et al., 2003; Herre et al., 2007; Rodriguez et al., 2009). In woody plants, most foliar endophyte species are acquired from the environment. Endophyte-free (generally <2% endophyte colonization) leaves can be produced by preventing spores from landing on leaf surfaces or by preventing leaf surfaces from becoming wet. Importantly, the identities of colonizing endophytes can be controlled (Arnold et al., 2003; Herre et al., 2007; Mejia et al., 2008). Experiments using these approaches have shown that foliar endophytes often enhance host defenses against pathogens and herbivores (Arnold et al., 2003; Mejia et al., 2008; Rodriguez et al., 2009). However, the actual mechanisms underlying the enhancement of host defense by FEF in woody plants are not established.

On one hand, many FEF produce antibiotic chemicals *in vitro*, and much attention has focused on the potential of these chemicals to provide enhanced host defense (Miller et al., 2002; Gunatilaka, 2006). On the other, it is well-known that plants respond genetically and physiologically to the presence of a wide variety of microbes (e.g., pathogens, non-foliar endophytes such as mycorrhizae and rhizobia, etc.) (Bailey et al., 2006; Friesen et al., 2011). However, the importance of the contribution of either endophyte-produced chemicals, or endophyte-induced enhancement of intrinsic host defenses to deter pathogens *in planta* is not known.

*Theobroma cacao*, the source of cacao beans, is a tropical tree species for which the ecology and systematics of fungal endophytes have been extensively studied, and for which there are complete genome sequences available (Crozier et al., 2006; Mejia et al., 2008; Hanada et al., 2010; Rojas et al., 2010; Argout et al., 2011; Motamayor et al., 2013). The composition of the FEF species assemblages in *T. cacao* is typical of other tropical tree species: it is highly diverse with few FEF species (e.g., *Colletotrichum tropicale*) consistently dominating the fungal assemblage, in any host plant that also includes many relatively rare species (Arnold et al., 2003; Crozier et al., 2006; Rojas et al., 2010). Several FEF species isolated from *T. cacao*, as well as microbes isolated from stems and pods of *T. cacao* and other host species have been shown to reduce leaf and pod damage caused by *T. cacao* pathogens and herbivores (Arnold et al., 2003; Evans et al., 2003; Rubini et al., 2005; Bailey et al., 2006; Mejia et al., 2008; Bae et al., 2009; Hanada et al., 2010; Krauss et al., 2010).

*Colletotrichum tropicale* occurs as an endophyte in a wide range of neotropical trees and is a dominant endophytic fungus in healthy leaves of *T. cacao* across natural and agricultural settings throughout Panama, and other parts of the New and Old World tropics (Rojas et al., 2010; Weir et al., 2012). In greenhouse experiments, E+ cacao plants inoculated and colonized with a mixture of endophyte species dominated by *C. tropicale* were more resistant to damage by the pathogen *Phytophthora palmivora* (Arnold et al., 2003). In agricultural field trials, *T. cacao* inoculated with *C. tropicale* showed lower incidence of black pod disease caused by *Phytophthora* spp. (Mejia et al., 2008). Thus, far, no evidence suggests chemical inhibition of *Phytophthora* spp. by *C. tropicale* (Mejia et al., 2008). Further, inoculations with *C. tropicale* have been shown to reduce herbivore damage in several tropical host plant species (Rojas et al., 2010; Van Bael et al., 2011, 2012).

Using 3347 and 17,247 unigenes *T. cacao* microarrays, quantitative PCR (RT-qPCR) and the completed sequences of the *T. cacao* and *Arabidopsis thaliana* genomes, we assessed changes in host gene expression in response to endophyte inoculation by comparing leaves inoculated with the foliar endophyte *C. tropicale* (E+) to endophyte-free, un-inoculated leaves (E−), in three experiments in which seedlings were grown under growth chamber conditions. In the leaf tissue from the first gene expression experiment we also determined changes in lignin and cellulose content. In addition, we used the functional annotation of significantly up- and down-regulated genes in the first two experiments as guides for expected changes in host phenotypic and physiological expression. Specifically, in two subsequent experiments examining phenotypic responses to endophyte inoculation, we measured photosynthetic responses and stable isotope ratios for nitrogen and carbon in E+ and E− leaves in plants grown under greenhouse conditions. In all inoculation experiments, E+ leaves showed significantly more endophyte colonization than E− leaves (see Table 1). Further, selecting a host gene (*Tc00g042540*, with a functional annotation suggesting defensive function) that was among the most highly up-regulated genes in E+ treatments, we conducted transient gene expression experiments to assess the effect of increased expression of this gene on host pathogen resistance in the absence of endophyte treatment. Finally, we conducted a third (time course) microarray experiment to document the dynamics of host genetic expression in response to endophyte inoculation through time.

**Table 1:** **Summary of experiments**.

	**Growth conditions**	**Analysis**	**Type and number of endophyte inoculations (#)**	**Time of sample collection**	Endophyte colonization Mean % ± SE		**Replicates # and Statistical test for endophyte colonization**
					E + leaves^*^	E − leaves^**^	
1st Microarray	Growth chamber	Genetics	Whole plant (4)	14 dpi^***^	80 ± 6%	1 ± 1%	*n* = 18 leaves per treatment, 32 leaf pieces per leaf;
		= Microarray (3347 unigenes)					
		= QPCR					
		Phenotype					Mann–Whitney U statistic = 144, chi^2^ with 1 *df* = 17.7, *p* < 0.001.
		= Phloroglucinol- HCL staining					
		= Raman microscopy					
2nd Microarray	Growth chamber	Genetics	Whole plant (1)	3 dpi	100 %	0 %	*n* = 3 E− and 2 E+ leaves, 32 leaf pieces per leaf.
		= Microarray (17247 unigenes)					
3rd Microarray	Growth chamber	Genetics	Whole plant (1)	0, 3, 7, and 14 dpi	40.6 %	0 %	*N* = 3E− and 3 E+ leaves, 32 leaf pieces per leaf.
		= Microarray (17247 unigenes)					
1st Phenotypic	Growth Chamber	Phenotype	Whole plant (10)	28 dpi	73.4 ± 4%	0.8 ± 0.8 %	*n* = 4 leaves per treatment, 32 leaf pieces per leaf;
		= Photosynthesis					Mann–Whitney U statistic = 16, chi^2^ with 1 *df* = 5.6, *p* < 0.05.
2nd Phenotypic^***^	Greenhouse	Phenotype	E+ and E− leaves paired within plant controlling for leaf age (10)	20 dpi	91.4 ± 1.7%	36.6 ± 3.5	*n* = 34 leaves per treatment, 32 leaf pieces per leaf;
		= N and C isotope measurements					paired analysis;
							Wilcoxon signed rank, *z* = 5.02, *p* < 0.000.

* *Theobroma cacao leaves inoculated with endophyte*.

** *Un-inoculated T. cacao leaves*.

*** *dpi, days post inoculation. In the cases of multiple inoculations, = days post last inoculation*.

**** *In the second phenotypic response experiment, we used a paired leaf design in which some leaves (oldest and youngest pairs) were inoculated (E+) and others un-inoculated (E−) in the same individual. The relatively high level of endophyte colonization in E− leaves in this experiment reflects both the environment from the greenhouse that makes it difficult to maintain leaves completely free of endophytes and the difficulty of inoculating one leaf while keeping the other endophyte free. This makes for a conservative test of the effects of C. tropicale on both nitrogen and carbon isotopes ratios*.

Collectively, these experiments indicate that FEF can exert profound influences on host gene expression that are related to changes in multiple aspects of the host's physiology, metabolism, anatomy, and resistance to pathogens and herbivores. Importantly, in this case, we selected *C. tropicale*, routinely the dominant species found in foliar endophytic fungal communities that establish in healthy *T. cacao* leaves (Rojas et al., 2010). In the case of this ecologically relevant endophyte, we found that enhanced resistance to pathogen damage results from increased expression of a host gene that is among the most highly up-regulated by *C. tropicale* inoculations, without the endophyte or its products being present in the host tissues. The generality of these findings on endophyte effects on host genetic and physiological expression is supported by the facts that (1) FEF are ubiquitous associates of plants worldwide, (2) that *C. tropicale* in particular is a dominant member of the endophyte community in many tropical plants (Rojas et al., 2010), and (3) that several studies document endophytic effects on host plant defense and physiology. Thus, our results demonstrate that endophyte presence and potential effects should be recognized and accounted for in the design and interpretation of studies of genetic and phenotypic expression in plants.

## Materials and methods

### Plant material and generation of plants symbiotic with endophyte (inoculation experiments)

Seeds from open pollinated *T. cacao* trees accession UF12, grown in a plantation in Charagre, Bocas del Toro province, Panama, were used for all microarray and phenotypic response experiments reported in this article. Endophyte free seedlings were generated at the Smithsonian Tropical Research Institute, Panama, as previously described (Arnold et al., 2003). In brief, cacao seeds were surface sterilized by immersing them in 0.5% sodium hypochlorite for 3 min and rinsed with sterile water before being placed for germination in plastic trays with soil (2:1 mixture of clay rich soil from Barro Colorado Island, Panama and rinsed river sand) and incubated in growth chambers. One-month-old seedlings were transplanted to individual pots (600 ml volume) containing the same soil mixture and kept in the growth chambers or transferred to a greenhouse depending on the experiment. Germination of seeds and seedling growth was done in Percival growth chambers (model I35LL, 115 volts, 1/4 Hp, series: 8503122.16, Percival Scientific, Inc., Perry, IA) with 12/12 h light/dark photoperiod and temperatures of 30 and 26°C, respectively. A summary of growth conditions and experimental designs is presented in Table 1.

Three microarray and two phenotypic response experiments were conducted to compare gene expression and anatomical and physiological phenotypes of endophyte inoculated *T. cacao* leaves (E+) with those of control un-inoculated *T. cacao* leaves (E−). Tissue used for the first microarray experiment was also evaluated for lignin and cellulose content in the leaf epidermal cells of E+ and E− plants. All microarray experiments and the first phenotypic response experiment were conducted in Percival growth chambers under the conditions detailed above and the second phenotypic response experiment was conducted in a greenhouse (Table 1). In all of these experiments, the endophyte treatment consisted of inoculation of cacao leaves with conidia (spores) suspensions of the endophyte *C. tropicale* strain 5101 (=CBS 124949) following previously described methods (Mejia et al., 2008). The percentage of endophyte colonization (number of leaf pieces with mycelia over total leaf pieces plated in culture media) per single leaf on E+ and E− leaves was measured as previously described (Mejia et al., 2008) and statistically analyzed using Wilcoxon signed rank test or Mann—Whitney U test (Table 1). The first microarray experiment was conducted to determine endophyte effect on host leaf gene expression using a 3347-unigenes spotted-oligo array. The second microarray experiment was conducted to test for consistency of the groups of host genes for which significant shifts in expression (up- or down- regulation) were produced by endophyte inoculations using a 17,247-unigenes Roche NimbleGen custom oligo array. The third microarray experiment was designed as a time course experiment to assess cacao leaf gene expression changes in E+ relative to E− leaves over a period of 2 weeks, with samples collection at 0, 3, 7, and 14 days post endophyte inoculation.

The first phenotypic response experiment focused on comparing photosynthesis (A_max_) between endophyte inoculated and un-inoculated plants. The second phenotypic response experiment was conducted in a greenhouse (using seedlings that were germinated and inoculated in the growth chambers) to confirm photosynthesis results from the first phenotypic response experiment under more natural conditions and to permit an assessment of endophyte induced shifts in carbon and nitrogen isotope signatures without the potential effects of isotope depletion often observed in relatively small closed chambers. Further, this experiment followed a paired leaf E+ and E−, on the same plant, design that permits greater statistical power.

### Microarray and RT-qPCR methods

For the first microarray experiment, we employed a custom 70-mer oligonucleotides array (*T. cacao* 3K microarray) spotted at The Pennsylvania State University (PSU) Core Genomics Facility. This array contained 3347 unigenes (spotted in duplicates) and a set of non-hybridizing oligos as negative and probe spike-in controls (Alien, Spot Validation System, Stratagene). These gene sequences were collated from several different EST sequencing projects and from other cacao genes sequenced at the time of microarray development, prior to the whole genome sequencing. A total of 2154 different *T. cacao* genes with their respective locus identifiers were represented in this array and 2015 of the genes contained *A. thaliana* locus identifiers and functional annotation based on *e*-value cutoff of 1^*^e-5 using TBLASTX (Altschul et al., 1990) on unigene sequences from this array against The Arabidopsis Information Resource (TAIR) database (Berardini et al., 2004). Samples used for microarray analyses consisted of six E+ and six E− mature leaves (equivalent to leaf developmental stage E; Mejía et al., 2012), each leaf from a different individual plant. These leaf samples were collected 14 dpi (Table 1), preserved in RNAlater according to manufacture's instructions (Applied Biosystems/Ambion, Austin, TX), and transported to PSU where RNA extractions were performed as previously described (Verica et al., 2004). Total RNA sample concentration and purity was assessed using a NanoDrop spectrophotometer and RNA quality was determined using an Agilent Bioanalzyer.

Hybridizations were performed by the Genomics Core Facility at PSU according to published facility protocols (http://www.huck.psu.edu/facilities/genomics-up/protocols/nimblegen-protocols). One μg of total RNA was amplified using mRNA amplification kit (Amino Allyl MessageAmp II™, Ambion, Austin, TX) prior to labeling and hybridization. aRNA was dye coupled with Cy3 or Cy5 (GE Health Care #RPN5661) and subsequently purified according to manufacturer's instructions. The samples were paired and each Cy3 labeled sample (1.5 μg) was combined with a Cy5 labeled sample (with 1.5 μg) in dye-swap design. The paired samples were mixed together, fragmented (using Ambion AM8740) and individually hybridized to a single array at 42°C for 18 h. A total of six arrays were hybridized resulting in a total of 12 measurements per treatment (six biological and six technical replicates). Arrays were washed to remove non-specifically bound target and were scanned with an Axon 4000A scanner.

For the second and third microarray experiment, we used a Roche Nimblegen oligonucleotide glass custom *T. cacao* gene expression 4X72k (four arrays of 72,000 probes) array, containing four probes of 50–60 mers in length for each of 17,247 unigenes (*T. cacao* 17K microarray, design ID 7114 manufactured by Roche). The microarray was designed based on EST sequences containing 6,853 contigs and 12,959 singlets from mixed cacao tissues (Argout et al., 2008), and 2781 unigenes resulting from 6572 ESTs generated from several previous cacao EST sequencing projects (Jones et al., 2002; Pugh et al., 2004; Verica et al., 2004). For the second microarray, three E+ and 4 E− leaf samples (leaf developmental stage D; Mejía et al., 2012) were collected at 3 dpi, each leaf from a different plant. For the third microarray, three E− leaf samples were collected at time 0, just prior to inoculation, and four E+ leaf samples (leaf developmental stage C–D; Mejía et al., 2012) were collected at 3, 7, and 14 dpi, each leaf from a different plant. Amplification, dye coupling, purification of labeled samples and hybridizations (one Cy5 and one Cy3 labeled sample mixed together per array) were performed also at the Genomics Core Facility at PSU.

The 17,247 unigenes on this microarray were blasted against TAIR9 CDS database and 11,225 unigenes had *A. thaliana* identifiers and annotations with the *e*-value cutoff of 1^*^e-5 using TBLASTX. The 6022 unigenes without *A. thaliana* annotations were blasted against the cacao genome database using BLASTN and 4931 out of 6022 unigenes had cacao identifiers and predicted full length gene sequences. Those 4931 cacao full length gene sequences were further blasted against TAIR9 CDS database and 4518 out of 4931 cacao genes to obtain their *A. thaliana* IDs at *e*-value cutoff of 1^*^e-5. At the end there were 11,445 genes with *T. cacao* locus identifiers and 8195 genes with *A. thaliana* locus identifiers (At) that were used for gene ontology (GO) categorization.

To verify microarray results from the first experiment, the RNA samples used for the microarray hybridization were subjected to RT-qPCR analysis (TaqMan® probe based system). Specifically, the expression of eight unigenes (representing six genes) that were highly up-regulated in the first microarray experiment was evaluated in the six E+ and six E− samples (three technical replicates each) used for first microarray experiment. Gene specific primers (Supplementary Table 1) were synthesized at the PSU Nucleic Acid Facility with a MerMade12 automated DNA synthesizer (Bioautomation, Plano, TX). Gene specific fluorescent probes were synthesized by Biosearch Technologies (Novato, CA). The fluorescent label used at the 5′ end on the cacao genes probes was 6-carboxyfluorescein (6-FAM) and quencher at the 3′ end of the gene probe was BHQ1 (Biosearch). The total volume of the PCR reaction was 25 μl and the mix included: 5 μl of cDNA (~12.5 ng), 12.5 μl 2× TaqMan® Universal Master Mix (#4304437, Applied Biosystems, Foster City, CA), 400 nmoles of each primer, and 200 nmoles of probe. The PCR reactions were ran in 96 well thin-walled PCR plates in an Applied Biosystems 7300 Q-PCR system (Foster City, CA) with the following reaction conditions; 2 min at 50°C, 10 min at 95°C, followed by 40 cycles of 15 s at 95°C, and 1 min at 60°C. Each sample was amplified in duplicate and the results were averaged. The expression of housekeeping genes *TcACT (Tc01g01090)* and *TcUBQ* (*Tc02g024050*) from cacao was also assessed and used to normalize the data. Amplification efficiency of all target and reference genes was calculated from the slopes of the dilution curves for each sample (E = 10^(−1/slope^)) (Bustin, 2004). Average efficiency for each gene was then calculated and used for efficiency data correction. The data normalization, efficiency correction, statistical randomization test and relative E+/E− expression ratios were computed using REST software (Pfaffl et al., 2002). Ratios (fold difference) with *p*-values less than 0.05 were considered significant.

### Microarray data analysis

Microarray data analyses of first and second microarray experiment were conducted using the Limma package of the Bioconductor software (Gentleman et al., 2004; Smyth, 2005). For the first microarray experiment, the background correction and normalization were performed using the Normexp and print-tip loess methods, respectively (Ritchie et al., 2007). The avedups function of Limma was employed for averaging log^2^ scale intensity values for duplicate spots from the normalized data. A gene-wise linear model was fitted to normalized average log scale intensity values of cDNA spots and the empirical Bayes method implemented in Limma was used for statistical analysis and assessment of differential expression (Smyth, 2004). A moderated *t*-test was conducted to compare endophyte inoculated and control leaf samples at 14 dpi and genes with *p* < 0.01 after Benjamini Hochberg (BH) correction for multiple testing correction were considered as significantly differentially expressed. The difference in expression between the controls (E−) and the treatments (E+) is presented as the log^2^ fold up- or down-regulation. For the second microarray experiment raw data was background corrected and normalized using the RMA algorithm available in the Limma package. Similarly as above, a gene-wise linear model was fitted to normalized data and empirical Bayes used for statistical analysis. A moderated *t*-test was conducted to identify genes with significant difference (*p* < 0.05 after BH correction for multiple testing) in gene expression between E+ and E− leaves at 3 dpi. The data from the third microarray experiment were analyzed with software ArrayStar 4 (DNASTAR). Pair files were imported to ArrayStar and processed using the RMA method and quantile normalization. Samples were grouped by time point and an *F*-test ANOVA was employed for finding genes with significant (*p* < 0.05 after FDR correction) difference in expression across the time points assessed. Data from the three microarray experiments are available at the NCBI Gene Expression Omnibus (GEO, accession number GSE54732).

### Functional characterization of microarray data

The potential function of differentially expressed cacao genes was assigned based on the function of the best hit after using BLAST against with the *A. thaliana* and *T. cacao* genomes. Genes with available *A. thaliana* loci accession numbers were classified according to GO terms using the tools for GO annotations at TAIR (Berardini et al., 2004) and at web-based agriGO (Du et al., 2010).

### Term enrichment analysis

To identify cacao gene sets particularly affected by endophyte inoculation, the list of differentially expressed genes with *A. thaliana* locus accession numbers, from the first and second microarray experiments, were imported into agriGO and singular enrichment analysis was performed. For each microarray experiment, the background list of genes for comparison was all the genes with *A. thaliana* locus identifiers present in the specific microarray. The statistical method applied was the hypergeometric test, Holm adjustment for multiple test correction, significance level 0.05 and complete GO analysis.

### Metabolic pathway analysis with MapMan

In order to perform metabolic pathway analysis of *T. cacao* genes with MapMan software (Thimm et al., 2004), we generated a mapping file with functional predictions of proteins using peptide sequences generated based on the *T. cacao*, Criollo genome sequence (Argout et al., 2011). The mapping file was generated with the Mercator pipeline for automated sequence annotation of the MapMan website using default parameters plus conservative and InterProScan (Lohse et al., 2014). Briefly a fasta file with 28,802 peptide sequences of cacao were uploaded to the Mercator tool for comparison with reference databases of protein sequences and a text mapping file with gene IDs assigned to MapMan bins (gene functional categories) was automatically generated. The file was consequently used for MapMan pathway analysis and visualization of differentially expressed genes into metabolic pathways. Files with list of differentially expressed genes and their respective expression fold changes between conditions for each of the first and second microarray experiments were imported to MapMan and genes visualized into metabolic pathways. Pathways explored in more detail included biotic stress (Pest/Pathogen attack), for identifying genes associated to plant-microbe interactions, and photosynthesis. The list of genes differentially expressed in the third microarray experiment was imported to MapMan for their functional categorization and later comparison with results from the first and second microarray experiments (Table 2).

**Table 2:** **MapMan Bins (functional categories of genes) with more elements (“genes”) affected by endophyte inoculations in the microarray experiments**.

**Code and name of functional category MapMan software**	**Number of elements affected per functional category**		
	**1st Microarray analysis**	**2nd Microarray analysis**	**3rd Microarray analysis**
29. Protein	57	176	26
35. Genes not assigned to any function	55	220	41
27. RNA	30	83	23
26. Miscellaneous function	21	47	3
34. Transport	16	33	3
30. Signaling	10	41	3
20. Stress	13	31	2
1. Photosynthesis	9	25	0
31. Cell	9	23	8
33. Development	8	25	6
17. Hormone metabolism	7	20	1
10. Cell Wall	5	19	0
11. Lipid metabolism	5	24	3
13. Amino acid metabolism	4	15	2

*The table is sorted by the top 14 categories of terms more affected by endophyte in the 1st microarray experiment, and provides a comparison of gene expression across the experiments*.

### Phloroglucinol-HCL staining of lignin in cacao leaves

Six E− and six E+ cacao plants used for the first microarray analysis were preserved in RNAlater. Three to four approximately 1 cm^2^ sections were cut from each leaf. The sections from each treatment (E+ and E−) were stained with 2% Phloroglucinol-HCL for 20 min. After the staining, 14 E+ and 18 E− sections were immediately assayed for the development of the purple color under an Olympus BX61Epi-Fluorescence Microscope (Olympus America Inc., Melville, NY) using a 10× objective. Images were acquired for with a Hamamatsu Orca-ER camera and processed with Olympus SlideBook 4.1 software. The purple color intensity (total pixel intensity) in the vascular tissue of each sample was measured using ImageJ software (http://rsbweb.nih.gov/ij/). Average intensity of each section was calculated and randomization tests indicated significant differences between the E+ and E− treatments (*p* < 0.001).

### Confocal raman microscopy

A WITec CRM200 confocal Raman microscope was used to acquire lignin spectral images from mature leaves (preserved in RNAlater) of the six E− and six E+ plants from first microarray experiment. In brief, the spectral images were generated by acquiring one spectrum (1 s integration time) at each pixel. Each pixel spacing was 500 nm. The lignin spectral images were generated using WITec's Witecproject software by integrating over the spectral interval 1495–1560 relative reciprocal centimeters (cm^−1^) at each pixel. Brighter regions in the spectral images correspond to higher total intensity within the region of integration.

Two-dimensional chemical images of lignin distribution in adaxial leaf epidermal cells were generated by integrating over spectral interval from 1495 to 1560 relative reciprocal centimeters (cm^−1^) at each pixel. We observed at 100× magnification that the cell corners and cell walls of both treatments had a greater intensity and thus greater lignin concentration than the inner part of the leaf cells. We performed a total of 12,000 individual scans at 40× magnification per E− and E+ treatments using random samples from all 6 biological replicates, including a approximately 240 cells per treatment. We recorded the overall carbohydrate distribution by integrating from 500 to 3000 cm^−1^. For this analysis Raman bands were assigned to cellulose or lignin components according to published literature and average spectra were calculated for each individual treatment (Gierlinger and Schwanninger, 2006).

### Phylogenetic analysis of tubulin genes

To provide a more detailed understanding of the potential involvement in cell wall modification of the different forms of the tubulin genes that were up-regulated in cacao (*TcTU*) in E+ plants, full-length protein coding sequences of these genes were compared to α (*TUA*) and β (*TUB*) tubulin sequences from *Populus tremuloides* and *A. thaliana*. To identify all tubulin genes from cacao, full-length CDS sequences of six *A. thaliana* (*AtTUA*) and eight *Populus* (*PoptrTUA*) genes were compared against *T. cacao* ESTs and Genome databases using TBLASTX (Altschul et al., 1990). Full-length CDS sequences of all tubulin genes from *A. thaliana, P. tremuloides*, and *T. cacao* (Supplementary Table 2) were aligned using Muscle software (Edgar, 2004). Additionally multiple sequence alignment was performed with all cacao tubulin genes and the EST sequences of tubulin genes up-regulated in the microarray.

A phylogenetic tree was inferred by neighbor-joining analysis of all α-tubulin gene sequences from *A. thaliana, P. tremuloides*, and *T. cacao* using Mega 4.0 software (Tamura et al., 2007). For this analysis, α-tubulin gene sequences from *Physcomitrella patens* (*PpTUA*) genes were used as outgroup. The pairwise deletion option was selected to address alignment gaps and missing data, and branch support was obtained through 2000 bootstrap replicates.

### Photosynthetic measurements

Photosynthesis related measurements were conducted in the first phenotypic response experiment at 27 dpi on leaves of developmental stage E (Mejía et al., 2012). Net CO_2_ uptake capacity was measured using a Licor 6400 portable photosynthesis meter (Li-Cor, NE). Light saturated rates of photosynthesis (A_max_) were obtained from light response curves of net CO_2_ uptake between 0 and 800 μmol photons m^−2^ s^−1^ under ambient CO_2_ conditions. Cuvete temperature was at 30°C. Air flow through the cuvettes was 500 μmol s^−1^.

### Carbon and nitrogen isotope measurements

Leaf samples (young and mature leaves corresponding to developmental stages C and E, respectively; Mejía et al., 2012) from the second phenotypic response experiment were dried and then shipped from Panama to Cornell University packed in desiccant. Upon arrival, the tissue was ground to a fine powder with a mortar and pestle and sub-samples of 2.55–3.15 mg were weighed using a microbalance (Model 4504MP8; Sartorius Corp. Edgewood, NY, USA). Tissue N and C content and δ^15^N and δ^13^C were measured using a CHN elemental analyzer (Model Carlo Erba NC2500; Thermo Finnigan, San Jose, CA, USA) coupled to a continuous flow isotope ratio mass spectrometer (Model Delta Plus; Thermo Finnigan, San Jose, CA, USA). Variation in repeated sample runs of the same material was ±0.01%. All analyses were conducted at the Cornell Stable Isotope Laboratory (COIL).

### Functional analyses of a gene involved in host defenses and plant-fungal symbioses

We conducted a series of experiments in which a *T. cacao* gene that is highly up-regulated in E+ treatments (*Tc00g042540*) was up-regulated in host tissues without the presence of *C. tropicale* or any of its products. The coding sequence *of TcChi1* in binary vector pGAM00.0511 (Maximova et al., 2006) was replaced with PCR amplified full length CDS of gene *Tc00g042540* (from genotype Scavina 6) under the control of high-level constitutive E12-Ω CaMV-35S promoter (Mitsuhara et al., 1996). The new binary vector and a control pGH00.0126 (Maximova et al., 2003) control vector (VC) were used to transiently transform cacao leaf discs from greenhouse grown mature Scavina 6 plants by *Agrobacterium* vacuum infiltration as previously described (Shi et al., 2013). Expression of *Tc00g042540* transgene was determined by RT-qPCR (primer set: RTF: ACTTGCAATATAGGGCGCTAGCCT and RTR: ACTTCTGGCGGGAAATACCACCTT) using Takara SYBR® premix EX TaqII kit (Clontech) according to the user's manual. Each reaction was performed in duplicates in Roche Applied Biosystem StepOne Plus Realtime PCR System (15 min at 94°C, 40 cycle of 15 s at 94°C, 20 s at 60°C, and 40 s at 72°C). The specificity of the primer pairs was examined by visualization on the 2% Agarose Gel and dissociation curve. Analysis was performed using *TcACT* (*Tc01t010900*) as reference gene (primer set: *TcACT*-RTF: AGCTGAGAGATTCCGTTGTCCAGA and *TcACT*-RTR: CCCACATCAACCAGACTTTGAGTTC).

The transient EGFP fluorescence was observed under a microscope as previously described (Maximova et al., 2003) and leaf areas with more than 80% EGFP fluorescence surface coverage were subjected to pathogen infection assay (Mejía et al., 2012; Shi et al., 2013). The right side of each leaf segment, delineated by the midvein and positioned adaxial side up, was inoculated with 3 agar plugs of *Phytophthora capsici* mycelium as previously described (6 VC and 6 *Tc00g042540* biological replicates). Three sterile agar plugs were placed at the left side of each leaf segment and used as negative controls. Inoculated leaves were then incubated at 27°C and 12:12 (Light: Dark) light cycle for 3 days before the evaluation of disease symptoms. Images were taken using Nikon D90 digital camera and measurements of each lesion size were performed using ImageJ software. Average lesion sizes were calculated from 18 replicates (6 leaf segments, 3 replicate inoculations per segment) and significance was determined by single factor ANOVA.

In order to assess the effects of increased expression of *Tc00g042540* on *P. capsici* DNA transcription (a proxy for pathogen metabolic activity and virulence), we used RT-qPCR to measure the ratio of *P. capsici* actin-coding DNA (*PcACT*) to cacao actin-coding DNA (*TcACT*), compared to controls (VC). Four lesions were collected as a 1.4 cm × 1.4 cm square surrounding the inoculation site and genomic DNA was extracted using TissueLyzer and DNeasy plant mini kit (Qiagen, Cat# 51304). *P. capsici* actin A (F: GACAACGGCTCCGGTATGTGCAAGG and R: GTCAGCACACCACGCTTGGACTG) and *TcACT* (*Tc01g010900*) were used as pathogen and host targets. RT-qPCR was performed as previously described (Wang et al., 2011).

## Results

### Endophyte regulation of host transcription

In all microarray experiments endophyte colonization was significantly higher in inoculated (E+) compared to un-inoculated (E−) leaves. See Table 1 for description and summary of all inoculation experiments. Analyses of gene expression in the first microarray experiment (a 3347-unigenes microarray) identified a total of 193 differentially expressed *T. cacao* genes (107 up- and 86 down-regulated, roughly 9% of all genes on this microarray) in E+ leaves relative to E− leaves (Moderated *t*-test, *p* < 0.01 after Benjamini-Hochberg multiple testing correction, Supplementary Tables 3, 4).

**Figure 1:**
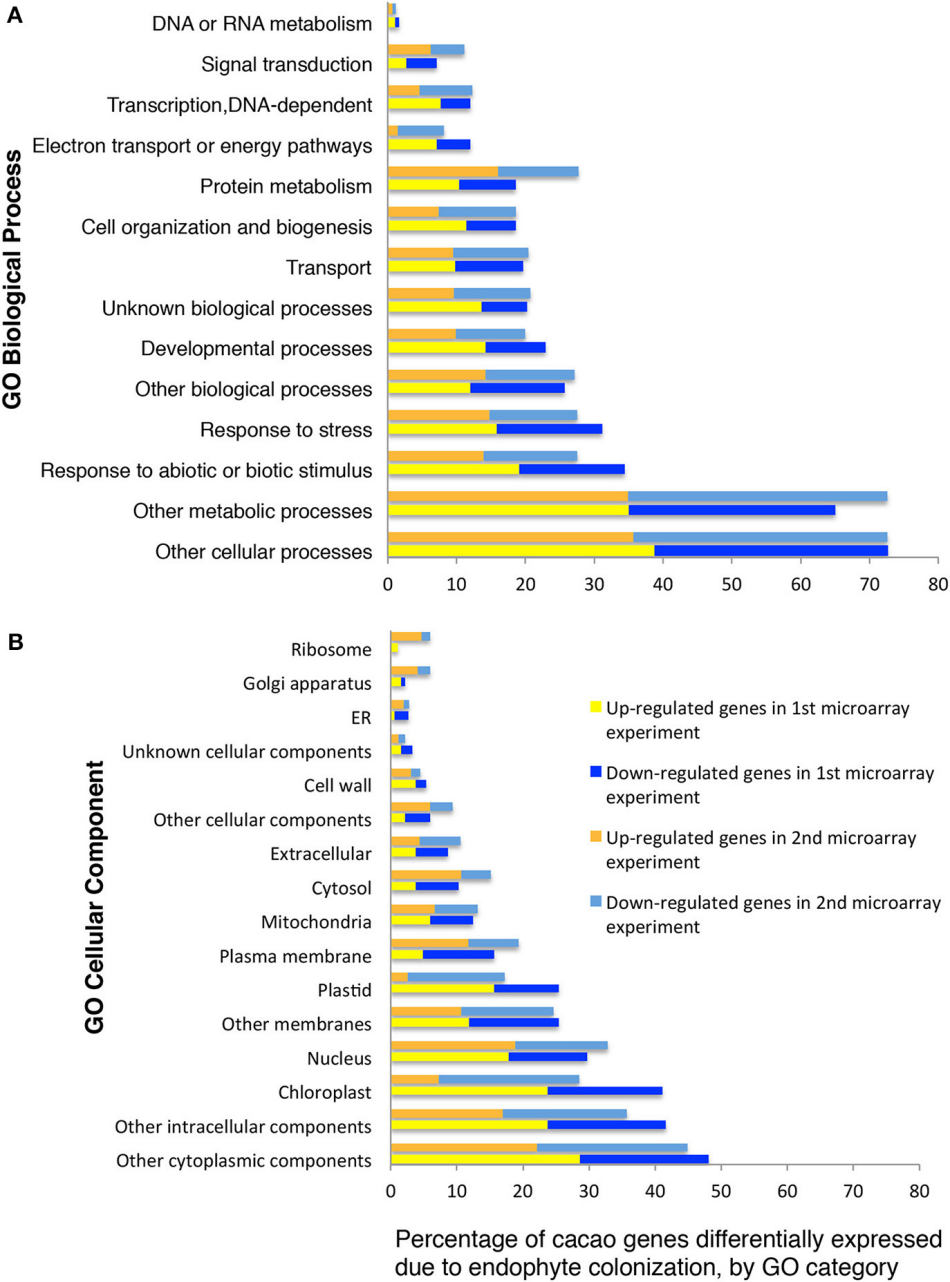
**Functional GO categorization of *T. cacao* genes with significant differences in expression due to endophyte *C. tropicale* inoculation**. Data represent the percentages of genes regulated by endophyte inoculation in different GO categories (i.e., 100 times the number of genes in each GO category divided by the total number of genes differentially expressed). **(A)** Genes sorted by Biological Process terms; **(B)** Genes sorted by Cellular Component terms. The GO categorization was made for 188 and 724 of the *T. cacao* genes differentially expressed in the first and second microarray experiments, respectively, and that had *A. thaliana* locus identifiers.

We classified 102 and 86 of the up- and down-regulated genes, respectively, using GO terms based on homology to *A. thaliana* (13 GO biological processes and 15 GO cellular components, methods, Figures 1A,B). A high proportion of the differentially expressed genes encode chloroplast proteins identified with photosynthetic function (Figures 1A,B) and there was a significant enrichment for GO terms “chlorophyll binding” (hypergeometric test, *p* < 0.05 after FDR). We performed RT-qPCR of eight unigenes (representing six genes) identified by this microarray analysis as being highly up-regulated by E+ treatments. Up-regulation was confirmed in all cases (Supplementary Table 5) and the more precise RT-qPCR analysis determined increases ranging from 2.9 to 53.2 fold, usually higher than values estimated by microarray hybridization.

A second gene expression experiment using a subsequently developed 17,247-unigenes microarray provided comparison with the first microarray experiment, and identified additional *T. cacao* affected by *C. tropicale* inoculation. Here, we identified significant changes in the expression of 856 *T. cacao* genes (7.5 % of the total), 433 up-regulated and 423 down-regulated genes in E+ relative to E− leaves (moderated *t*-test and *p* < 0.05 after BH adjustment for multiple testing correction, Supplementary Tables 6, 7), and significant GO enrichment for several chloroplast terms (Supplementary Figure 1).

Functional analyses of genes based on GO and MapMan (Thimm et al., 2004) terms also indicated that many of the regulated genes in first and second microarray experiments are involved in cellulose and lignin deposition and host cell wall hardening, nitrogen metabolism, photosynthesis, and biotic stress responses (Supplementary Tables 8, 9). The top 10 GO and MapMan (i.e., protein, signaling, RNA, cell wall, photosynthesis, hormone metabolism, miscellaneous function, cell, stress, and genes not assigned to any function) gene categories with more genes regulated by endophyte inoculation were consistent between the first and second microarray experiments (Figure 1, Table 2).

**Figure 2:**
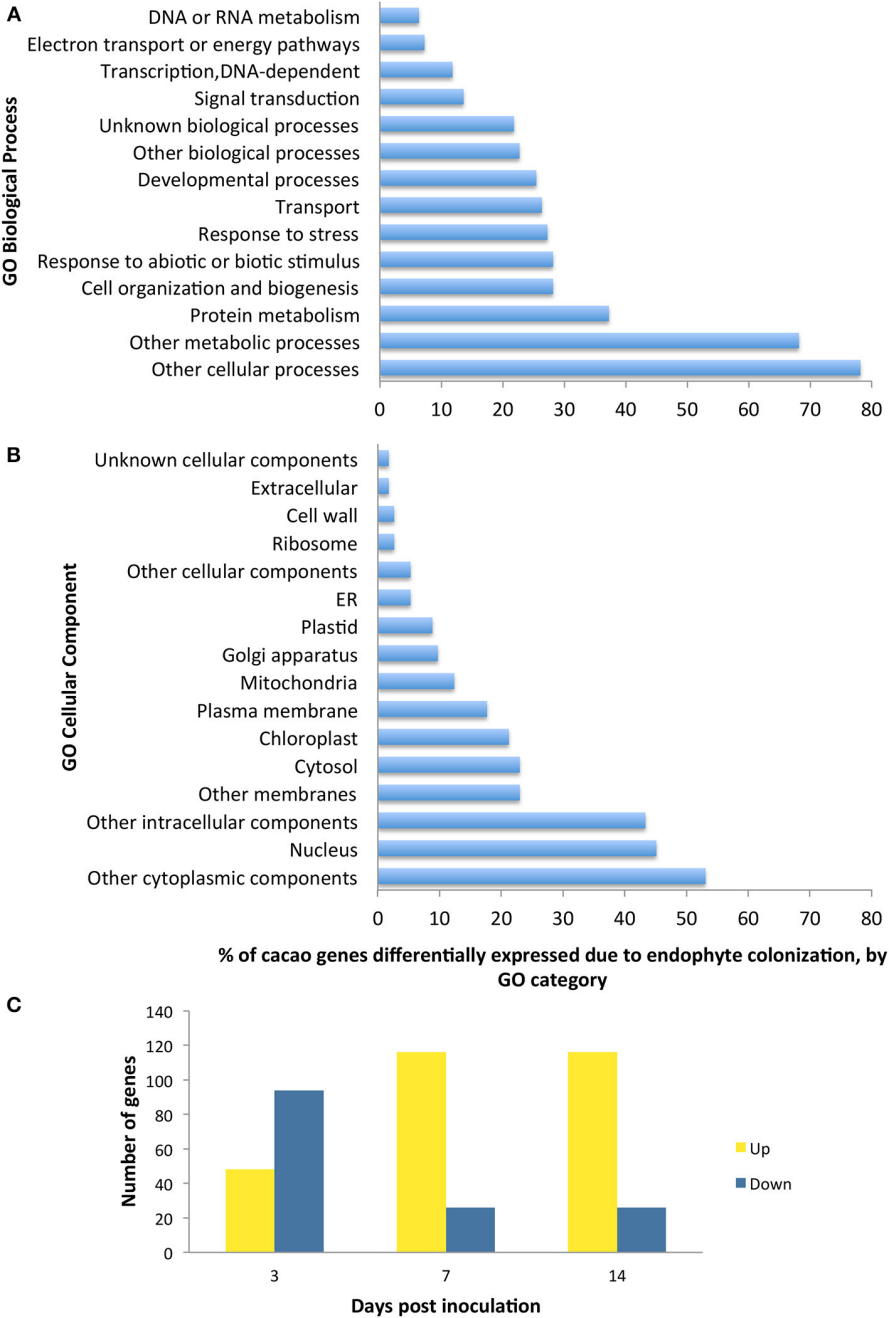
**Functional GO categorization of *T. cacao* genes with significant changes in expression due to endophyte *C. tropicale* inoculation across four time points evaluated during 2 weeks post inoculation**. A total of 142 genes showed significant difference in expression (*F*-test ANOVA, *p* < 0.05 after FDR correction). **(A)** Genes sorted by Biological process terms; **(B)** Genes sorted by Cellular Component terms; **(C)** Number of genes up-regulated and down-regulated at specific time points relative to time 0. Note that the relative proportion of genes up- or down-regulated changes through time after inoculation, highlighting the utility of standardizing time of sampling in studies of host gene induction by symbiotic mircoorganisms.

We conducted the third microarray experiment (time course) to view the dynamics of gene expression changes in E+ relative to E− leaves over a period of two weeks. We found 142 genes with significant changes in expression through the entire time course and with fold changes in expression between time points ranging from 1 to 7.4 [*p* < 0.05 after false discovery rate (FDR) correction, Supplementary Table 10]. We observed that the initial host response was characterized largely by down regulation, following by up-regulation of host genes (Figure 2). We classified 112 of these genes according to GO terms based on homology to *A. thaliana* (14 GO biological processes and 16 GO cellular components) and found that GO categories of host genes with more genes affected by the endophyte under biological processes and cellular component were similar to the first and second microarray experiment (Figure 2).

### Endophyte regulation of gene expression for defense

Inoculation and successful host colonization by the endophyte, *C. tropicale*, causes significant up- and down-regulation of scores of host genes involved in defense against biotic stresses (i.e., pathogens and herbivores). Notably, genes affected were involved in the ethylene signaling and defense response pathways as well as signaling proteins (e.g., receptor kinases, Supplementary Table 8). Further, E+ inoculations also produced changes in the expression of genes associated with the synthesis, modification, and degradation of cell wall (e.g., Proline rich proteins; Bradley et al., 1992); peroxidases and components of the jasmonic acid defense pathway; pathogenesis related proteins (e.g., PR4 protein); redox state proteins; genes coding beta glucanases (defense against pathogenic fungi), heat shock proteins, transcription factors, proteins involved in secondary metabolism and proteolysis; and other genes relevant to plant-microbe interactions such as NPR3, nodulin, and endochitinases (Supplementary Table 8).

### Endophyte regulation of transcription of host cell wall genes

Twenty of the significant genes between E+ and E− leaves in the first and second microarray experiment were associated to cell wall biogenesis (12 up-regulated and 8 down-regulated genes, Supplementary Table 8). In both of these microarray experiments, we observed significant up-regulation of a gene (*Tc01g035310*) coding for a putative proline rich protein (Supplementary Table 4) previously linked to hardening of cell walls (Bradley et al., 1992). We also observed two up-regulated putative tubulin genes *(TcTUA1* and *TcTUA5*). *TcTUA1* is similar to *PoptrTUA3* and *PoptrTUA5* found in *Populus tremuloides*, two Class I α tubulin genes shown to be abundant in xylem and preferentially expressed in wood-forming tissue (Supplementary Figure 2) (Oakley et al., 2007). Microtubules are well-known to be involved in cellulose biogenesis through linkages with the cellulose synthesis machinery.

**Figure 3:**
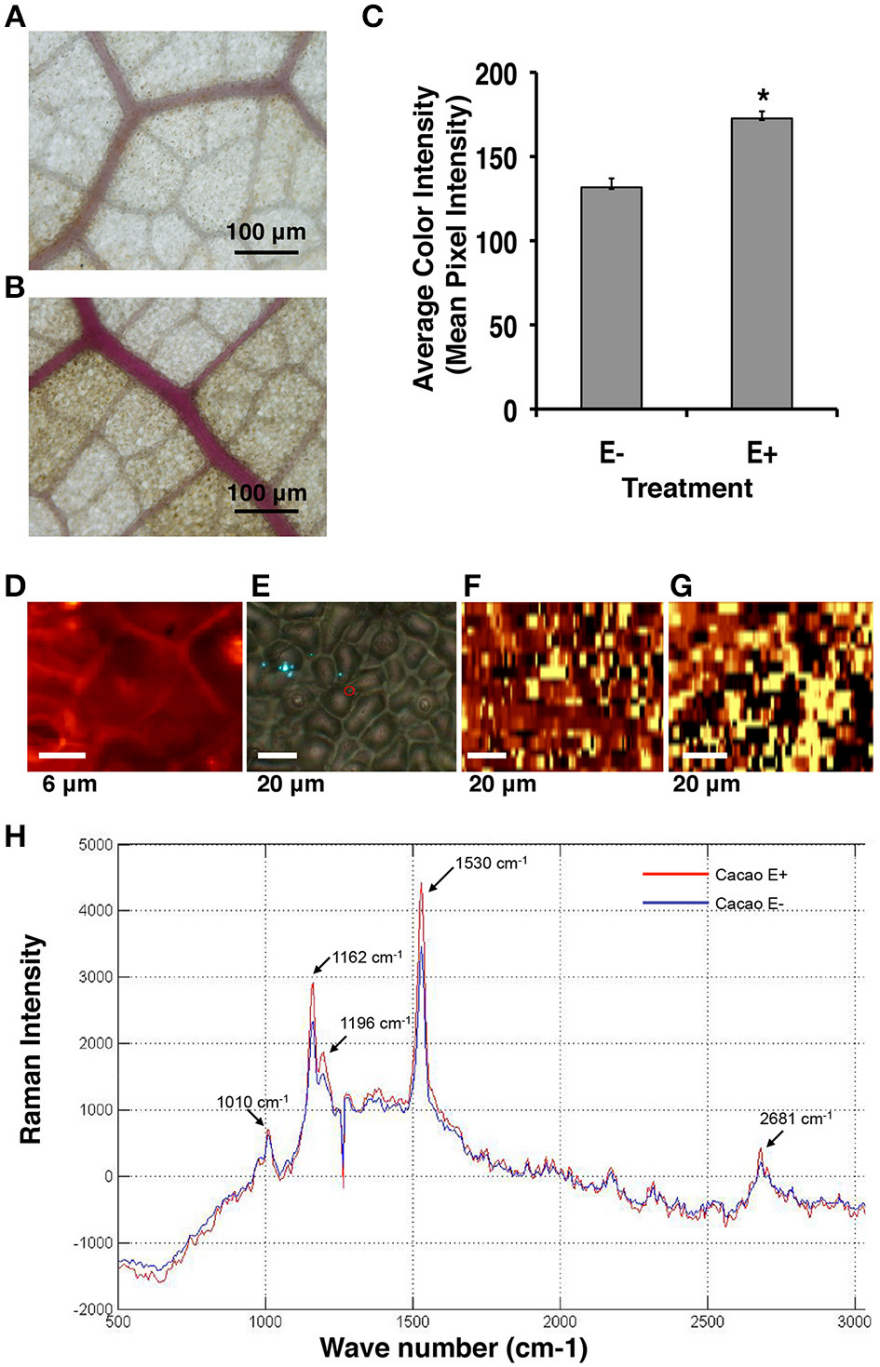
**Endophyte inoculated *T. cacao* leaves (E+) have higher lignin content than un-inoculated (E−) leaves. (A)** Phloroglucinol-HCL stained E− leaf; **(B)** Phloroglucinol-HCL stained E+ leaf; **(C)** Comparing color intensity of the E− and E+ stained leaves (means ± SE). E+ leaves exhibited higher purple staining of the vascular tissue compared to E− leaves indicating ~23% higher lignin accumulation in the E+ tissues. ^*^Significance determined at *p* < 0.001. **(D–H)** Raman imaging and spectrometry reveal higher concentration of lignin and cellulose content in E+ epidermal leaf cells relative to E−. **(D–G)** Representative images of epidermal leaf cells: Bright yellow areas indicate high concentration of lignin and dark black regions indicate very low concentration of lignin; **(D)** Two-dimensional Raman image (false color) of lignin spatial distribution in the epidermal cells at 100×; **(E)** Bright field (white light) image of epidermal leaf cells at 40×; **(F)** Two-dimensional image of lignin spatial distribution in E− leaf cells at 40×; **(G)** Two-dimensional image of lignin spatial distribution in E+ leaf cells at 40×; **(H)** Spectral bands of lignin (1530 cm^−1^) and cellulose (1162 cm^−1^) (see Section Materials and Methods). These results were very similar to results from the Phloroglucinol-HCL staining assay.

Consistent with the microarray data, Phloroglucinol-HCL staining and Raman microscopy analyses indicated that the amount of lignin and cellulose was indeed significantly greater in E+ relative to E− leaves used in the first microarray experiment for genetic analyses (Figures 3A,B). The comparison of the mean intensity values for E+ and E− samples indicated a ~23% increase of lignin content in the epidermal cells of E+ leaf samples (Figure 3C). For Raman microscopy, bands were assigned to cellulose or lignin components (Gierlinger and Schwanninger, 2006). Average spectra values revealed that epidermal leaf cells from inoculated plants contained ~23% more lignin and ~20% more cellulose (Figures 3D–H).

### Endophyte down-regulation of chloroplast genes and reduced A_max_

Analysis of the first and second microarray experiments indicated that many chloroplast and photosynthesis related genes were down-regulated in E+ plants (Figure 1, Supplementary Tables 4, 7). These included genes encoding for: PSAD-2 (photosystem I subunit D-2), PSBY photosystem II PsbY protein, RuBisCO small subunit 1A (RBCS-1A) among many others. Additionally several chloroplast related genes were also regulated by the endophyte inoculation in the third microarray experiment (Figure 2). Consequently, we evaluated the effects of E+ treatments on maximum rates of host photosynthetic activity (A_max_) and other photosynthetic parameters under growth chamber conditions in which entire plants were either E+ or E−.

**Figure 4:**
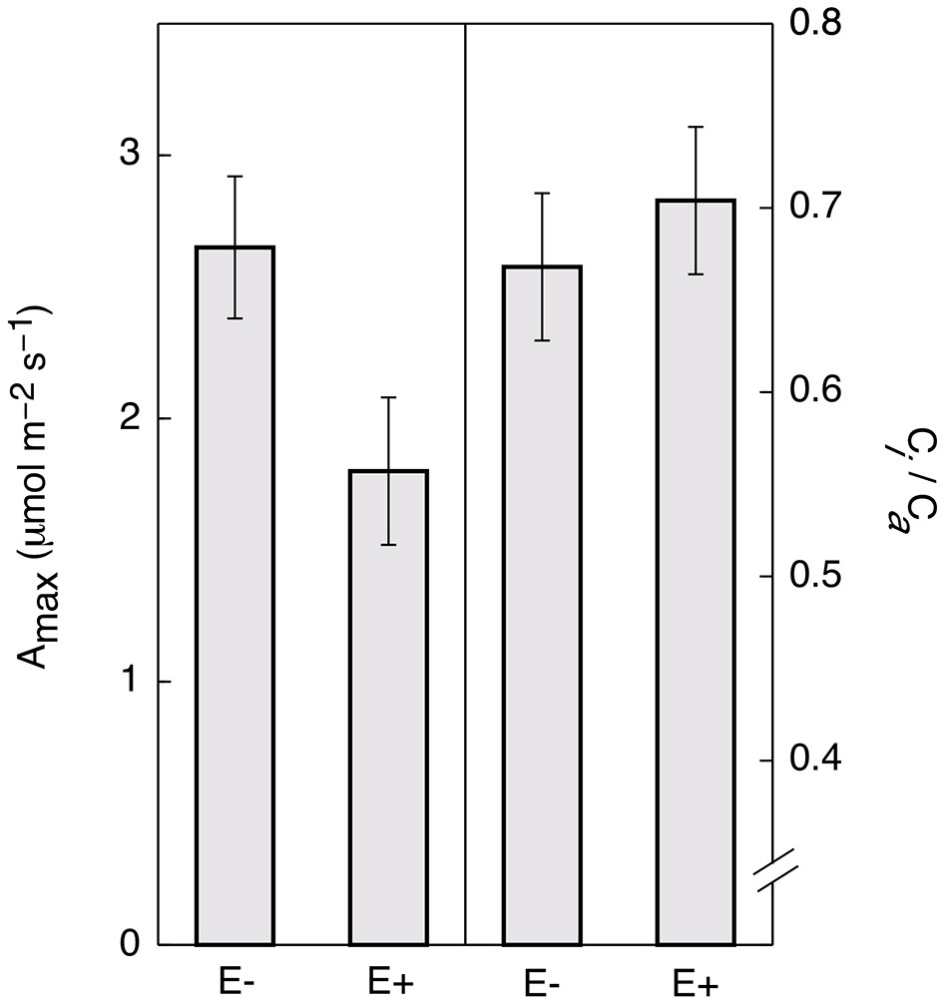
**Endophyte inoculation reduces maximum rates of photosynthesis (Amax) in *T. cacao* leaves**. Maximum photosynthesis (mean Amax ± SD) in endophyte *C. tropicale* inoculated (E+) cacao leaves was reduced compared to un-inoculated (E−) (left), Student's two-tailed *t*-test: *p* < 0.0001. The ratio of internal to ambient CO_2_ (mean ci/ca ± SD) was not affected (right).

Controlling for leaf age and time of day, E+ leaves maintained significantly lower light saturated rates of maximum photosynthesis rates (A_max_) throughout the day (~32%, Figure 4). There was no significant difference in *c_i_/c_a_*(the ratio of intercellular to ambient CO_2_ concentrations; Figure 4) determined by gas exchange (i.e., the term *c_i_* in traditional gas exchange experiments refers to the CO_2_ concentration in the substomatal cavities rather than to the concentration at the point of carboxylation in the chloroplast) indicating that the observed reductions in photosynthesis were not due to increased stomatal limitation of CO_2_ uptake. These results are consistent with the genetic data, suggesting that endophytes negatively affect photosynthetic activity by down-regulating many associated genes.

### Endophyte effects on carbon and nitrogen isotope ratios

We also encountered endophyte-induced changes in expression of many genes involved in nitrogen metabolism. GO categorization of the significant genes in the first and second microarray experiments identified a total of 115 genes in the category “nitrogen compound metabolic” (61 up-regulated and 54 down-regulated, Supplementary Table 9), providing strong evidence for an effect of endophytes on host nitrogen metabolism. These genes were also classified in other nitrogen GO categories: cellular nitrogen compound biosynthetic process (23 genes), cellular nitrogen compound metabolic process (36 genes), and regulation of nitrogen compound metabolic process (52 genes). Key nitrogen metabolism genes affected by *C. tropicale* included glutamine synthetase 2 and glutamate synthase in both, first and second microarray experiments.

Given that inoculation with *C. tropicale* influences photosynthetic properties (first phenotypic experiment) that correspond to the changes in expression of several photosynthesis-related genes (above), we conducted a second phenotypic experiment under greenhouse conditions to examine stable carbon isotope composition of E+ and E− plants. Based on the observed effects of endophyte inoculation on nitrogen metabolism, we also used this greenhouse experiment to examine stable nitrogen isotope composition in the same plants. In this experiment we controlled for among plant effects by comparing C and N isotope signatures for paired E+ and E− treated leaves of the same age on the same plants. We found that inoculation with *C. tropicale* significantly enriched both foliar δ^13^C and δ^15^N compared to un-inoculated controls (Table 3).

**Table 3:** Enrichment^*^ of Carbon 13 and Nitrogen 15 isotopes in *T. cacao* leaves inoculated with endophyte *C. tropicale*.

	**E−**	**E+**	***p*-value**
Mean δ^13^C ± SE in mature leaves	−34.13 ± 0.11	−33.49 ± 0.14	<0.003^**^
Mean δ^13^C ± SE in young leaves	−32.41 ± 0.06	−32.03 ± 0.10	<0.002^***^
Mean δ^15^N ± SE in mature leaves	4.96 ± 0.09	5.46 ± 0.10	<0.001^***^
Mean δ^15^N ± SE in young leaves	4.69 ± 0.09	5.07 ± 0.09	<0.0001^***^

* *The observed enrichment of 15N and 13C isotopes in E+ plants occurs over relatively short periods (a few days to weeks), with enrichment increasing over time*.

** *Paired t-test*.

*** *Wilcoxon signed rank test*.

### Functional analyses of a gene involved in host defenses and plant-fungal symbioses

**Figure 5:**
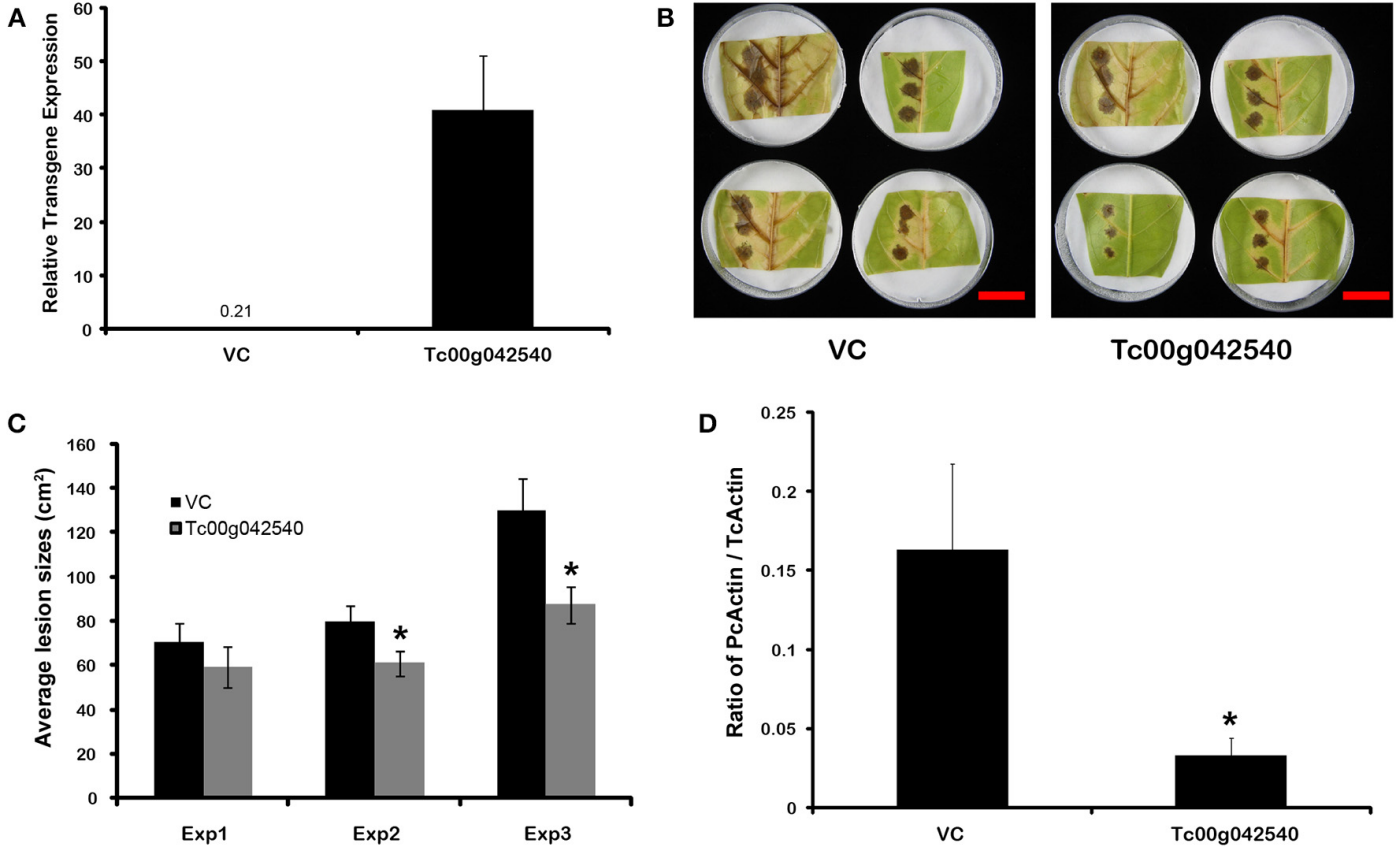
**Transient over-expression of endophyte inducible gene *Tc00g042540* (a putative trypsin inhibitor) reduces *Phytophthora capsici* damage and virulence in *T. cacao* leaves**. ^*^Significant differences for all analyses were determined by single factor ANOVA (*p* < 0.05). **(A)** *Tc00g042540* gene expression relative to *TcACT* (*Tc01g010900*) at 2 days after *Agrobacterium*-mediated transformation (means ± SE). The basal expression of *Tc00g042540* in Vector control (VC) tissues is only 0.21; **(B)** Representative images of the variation in lesions sized caused by *P. capsici* on VC and *Tc00g042540* over-expressing leaves 2 days after pathogen inoculation (scale Bar = 3 cm); **(C)** Average lesion sizes of three independent transformation experiments 2 days after pathogen inoculation (means ± SE). Bars represent means of 12 individual lesions per treatment per experiment; **(D)** Ratio of actin expression of the pathogen compared to host, a proxy for pathogen virulence, is higher in VC than in *Tc00g042540* over-expressing leaves. This was quantified by RT-qPCR (means ± SE), 2 days after pathogen inoculation.

The potential for defensive function of a specific gene up-regulated by endophyte treatments in the absence of direct endophyte inoculations was evaluated using transient transgene expression coupled with pathogen assays. A gene of previously unknown function (*Tc00g042540*) was among the most highly up-regulated cacao genes found in E+ treatments in the first microarray experiment. This gene had been annotated as a 21 kDa seed protein (Argout et al., 2011) and has a weak sequence similarity to a known trypsin inhibitor (functional prediction from Mercator and MapMan analyses; (Lohse et al., 2014)) thus suggesting a defensive function. This gene was cloned by PCR amplification from cacao genotype Scavina 6 and transiently over-expressed in cacao leaves from the same genotype. The transformed cacao leaves had less damage caused by pathogen *P. capsici* than leaves transformed with a control vector (Figure 5). Thus, a *T. cacao* gene that is up-regulated by inoculation with *C. tropicale* (E+) can limit pathogen damage without endophytes or any endophyte-produced chemicals physically present in the host plant tissue.

## Discussion

FEF are ubiquitous associates of healthy, apparently asymptomatic plants, in nature. Experiments have shown that host plants inoculated with endophytes are often more resistant to pathogen and herbivore damage (Arnold et al., 2003; Campanile et al., 2007; Mejia et al., 2008; Van Bael et al., 2009). In particular, we show that *T. cacao* seedlings inoculated (E+) and colonized with *C. tropicale*, the FEF species most commonly encountered in healthy *T. cacao* leaves, exhibit markedly different patterns of gene expression compared to un-inoculated leaves (E−). Across the microarray experiments, gene categories that show consistent effects of endophyte inoculations include: defense related (e.g., ethylene pathway, receptor kinases), cell wall development, chloroplast, and nitrogen metabolism (Figure 1). Further, these endophyte-induced effects on the expression of specific functional genes correspond to endophyte-induced changes in phenotypic expression in the host.

Our genetic and experimental results indicate that inoculation of the *T. cacao* with *C. tropicale* enhances the expression of large suites of host genes that are known to contribute to host defense against pathogen and herbivore attack. Specifically, the transient expression experiments show that over-expression of a gene (*Tc00g042540*) that is highly up-regulated in E+ treatments significantly decreased pathogen damage to its host. Importantly, at least in the case of this fungal species that dominates endophyte communities in healthy *T. cacao* (*C. tropicale*), increased host resistance is not due to any direct endophyte effect on pathogens (e.g., chemicals that endophytes might produce independently of the host (Mejia et al., 2008; Higginbotham et al., 2013), or even the physical presence of the endophytes. Instead, this experiment shows that endophytes can affect pathogen damage indirectly, by inducing increased expression of host genes that are demonstrably effective in enhancing disease resistance. Additional studies are needed to assess the relative contribution of direct and indirect effects of different foliar endophyte species (both systemically and locally) on host resistance to different pathogens (Mejia et al., 2008; Adame-Álvarez et al., 2014). Based on previous studies (Herre et al., 2007; Mejia et al., 2008), we expect that direct, chemical effects will be more important in some endophyte species than others, and that the specific chemicals contributing to defense will vary among species. Ultimately, the composition of the entire endophyte community will in large part determine levels of host resistance to individual pathogen and herbivore species that vary in their sensitivities to host defense and different chemicals produced by different components of the endophyte community (Arnold et al., 2003; Herre et al., 2007; Mejia et al., 2008; Adame-Álvarez et al., 2014).

More generally, among the genes and pathways identified with disease resistance in *A. thaliana* and other host plants, *C. tropicale* inoculation causes the up-regulation of many key components of the ethylene defense pathway in *T. cacao*, as well as several signaling genes (e.g., receptor kinases). E+ plants exhibited relative up-regulation of the ethylene overproduction protein 1 (*ETO1*) and the ethylene forming enzyme (*EFE, ACC oxidase*), up- and down-regulation of two ethylene responsive element binding family protein genes (*EREBP*), and down-regulation of the EIN3-binding F-box protein 1 (*EBF1*). Among the primary targets of the ethylene defense are necrotrophic pathogens such as *P. palmivora* which causes less damage in E+ cacao leaves inoculated with *C. tropicale* and other endophytes (Arnold et al., 2003; Mejia et al., 2008; Dodds and Rathjen, 2010). Further, receptor kinases play an important role in detection of pathogenic and non-pathogenic microbes at the cellular surface or within the cell (Dodds and Rathjen, 2010). These results suggest that one of the effects of the endophytes is to prime the host plant's defensive responses to pathogens for increased early detection by receptor kinases at cellular surfaces and subsequent intracellular responses mediated by cytoplasmic kinases and the ethylene transduction pathway. Additional studies are needed to determine the degree to which different endophyte species up-regulate different receptor kinases and other signaling genes (potentially affecting which pathogens might be recognized, and which host defense pathways are preferentially induced).

Interestingly, EIN3 and other components of the ethylene transduction pathway are required for the root endophytic fungus *Piriformospora indica* to balance beneficial and non-beneficial traits in its symbiosis with *A. thaliana* (Camehl et al., 2010). The conspicuous induction of the ethylene pathway is also involved in other mutualistic plant microbial symbioses (Pieterse et al., 1998; Khatabi and Schäfer, 2012), among them mycorrhizal-root and rhizobia-nodule associations. Although it appears that many fungal and bacterial mutualists have co-opted the ethylene pathway as a key component of their interactions with their plant hosts, the specific ways in which pathogens and mutualistic endophytes differentially influence expression and function of the ethylene pathway remain to be defined.

Inoculation and colonization with *C. tropicale* increased the lignin (~23%) and cellulose (~20%) content of E+ leaves of *T. cacao*. Recent comparative studies showed that plant species with relatively high cellulose content and lamina density (mass per unit volume of leaf) also tend to exhibit high leaf fracture toughness, and that these two traits together correlate with reduced herbivory rates and increased leaf lifespan across tropical plant species (Coley, 1983; Kitajima et al., 2012). Previous experimental studies with other host plant species (*Cucumis sativus, Merremia umbellata*, and *T. cacao)* show that *C. tropicale* inoculations can reduce leaf damage by tropical generalist herbivores, specifically leaf–cutting ants (Van Bael et al., 2012; Estrada et al., 2013). While further experiments are needed, evidence suggests that endophyte-induced increases in lignin and cellulose in their hosts' cell walls (in combination with endohyte-produced chemicals; Estrada et al., 2013) could contribute to the defense against pathogens and herbivores.

The decrease (~32%) in A_max_ with *C. tropicale* inoculation (Figure 4) is consistent with the observed down-regulation of many genes coding for proteins in the photosynthetic pathways. Further, the observed enrichment in foliar δ^13^C (~2%, Table 3), the increased lignin and cellulose deposition in the cell wall, increased hyphal presence inside leaf tissues, and lack of *c_i_/c_a_* differences between E+ and E− leaves are also consistent with an increase in mesophyll resistance to CO_2_ diffusion. Increased mesophyll resistances (i.e., increased resistances to CO_2_ diffusion between the sub-stomatal cavity and the site of enzymatic fixation within the chloroplast) would decrease the rate of CO_2_ diffusion to the point of carboxylation within the chloroplast and limit the rate of photosynthesis, and reduce the enzymatic discrimination against the heavier carbon isotope. Additionally, E+ plants exhibited down-regulation of host genes that can promote photosynthetic function by eliminating reactive oxygen species that interfere with electron transport (e.g., ascorbate peroxidase 1, among others). Although more detailed studies of the mechanisms underlying the observed decreases in A_max_ and the increases in foliar δ^13^C in E+ plants are needed, endophytes clearly reduce the photosynthetic competence of their hosts.

The consistent enrichment of the foliar isotope ratio of nitrogen in the presence of endophytes observed in this study was perhaps surprising. Foliar nitrogen isotopes are thought to be a reflection of the soil solution δ^15^N (a consistent value in the controlled greenhouse conditions of this study) modified by within-plant fractionations that are thought to be relatively constant for a given plant species (Evans, 2001; Craine et al., 2009). The ^15^N enrichment in E+ plants observed in this study (0.3–0.5 δ^15^N, Table 3) suggests either that endophytes alter the processes of nitrogen uptake into and/or reduction within host plants, or that endophytes increase the preferential loss of ^14^N from the leaf tissue, or both. Plant leaves are known to produce significant amounts of nitrogen trace gases (Sparks, 2009) and it is possible that increases in gaseous losses could lead to enrichment of the heavy N isotope in foliar tissue. Further, if endophytes utilize nitrogen from the host through heterotrophy of host products or tissues (as seems likely), then nitrogen could cycle between endophyte and host, experience fractionating losses, and enrich ^15^N in leaf tissue over time (see Table 3). The ultimate cause of enriched foliar δ^15^N in the presence of endophytes observed in this study is unclear. Nonetheless, endophytes appear to fundamentally alter nitrogen metabolism in their host. Detailed studies are needed of the specific nitrogen metabolism genes that are influenced by E+ treatments and the degree to which endophyte heterotrophy of host tissues contribute to the significant shifts in δ^15^N.

## Conclusion

Several experiments show that both foliar and other types of endophytes affect many characters that are usually thought of as “plant” characters (e.g., ability to resist either biotic or abiotic stresses, physiological responses, chemical and even stable isotope composition, and genetic composition and expression). Foliar endophytes can benefit their hosts through increased resistance to pathogen and herbivore damage. This study identifies several likely genetic and physiological mechanisms that can work alone or in combination to produce these effects. Importantly, in the case of this ecologically relevant, dominant endophyte species, we demonstrated that increased resistance to pathogen damage can occur in the absence of any direct endophyte treatment or any chemical that they produce. Up-regulation of host gene products that are themselves up-regulated as part of the host response to endophytes appear to be responsible. Yet these and other experiments also show clear costs of endophytes: reduced photosynthetic capacity and altered nitrogen metabolism. It appears that in a pathogen- and herbivore-free world, foliar endophytes would represent a net loss to the host, but are in fact providing a net benefit that increases as pathogen and herbivore pressure increases. Further, we note that asymptomatic endophytic fungi are often close relatives of fungal pathogens (Freeman and Rodriguez, 1993; Mejia et al., 2008). Ultimately a detailed genetic understanding of what makes an endophyte a neutral or even a beneficial symbiont is needed in the context of what makes some congeneric species pathogens (Freeman and Rodriguez, 1993).

Studies of genotypic and phenotypic expression in plants (e.g., defense, physiology, etc.) usually do not control for, or even consider, the potential effects of endophyte colonization. This and other studies have shown that, over the course of even a few days, the effects of foliar endophytes on host genetic and phenotypic expression can be rapid and large [e.g., marked reductions in pathogen and herbivore damage (Craine et al., 2009; Rodriguez et al., 2009; Friesen et al., 2011; Van Bael et al., 2012; Estrada et al., 2013); ~23% increase in leaf lignin content, ~20% increase in cellulose; ~32% reduction in A_max_; shifts in carbon (~2%) and nitrogen (~9%) isotopic signatures]. Studies of host plant genetic and phenotypic expression should be designed and interpreted with potential endophyte effects on their host clearly in mind (Arnold et al., 2003; Bailey et al., 2006; Herre et al., 2007; Mejia et al., 2008). We have shown the multiple effects that one dominant foliar endophyte species can have on its host's genetic and phenotypic expression. Plants normally harbor many endophyte species (e.g., foliar fungal or bacterial endophytes, arbuscular mycorrhizae, or nitrogen fixing bacteria, etc.), and studies of their separate and combined effects on host genetic and phenotypic expression are needed.

## Author note

Recent published and in press work suggests similar patterns and mechanisms are responsible for symbiont-conferred protection against natural enemies across both plants and insects (see Douglas, 2014; Gerardo and Parker, 2014).

### Conflict of interest statement

The authors declare that the research was conducted in the absence of any commercial or financial relationships that could be construed as a potential conflict of interest.
