# Supplementary material for: Pervasive effects of a dominant foliar endophytic fungus on host genetic and phenotypic expression in a tropical tree
Source: Front Microbiol. 2014 Sep 12;5:479. doi: 10.3389/fmicb.2014.00479 (PMC4162356; doi:10.3389/fmicb.2014.00479)
Supplement: Supplementary file 1 [file Presentation1.PDF]

Supplementary Information

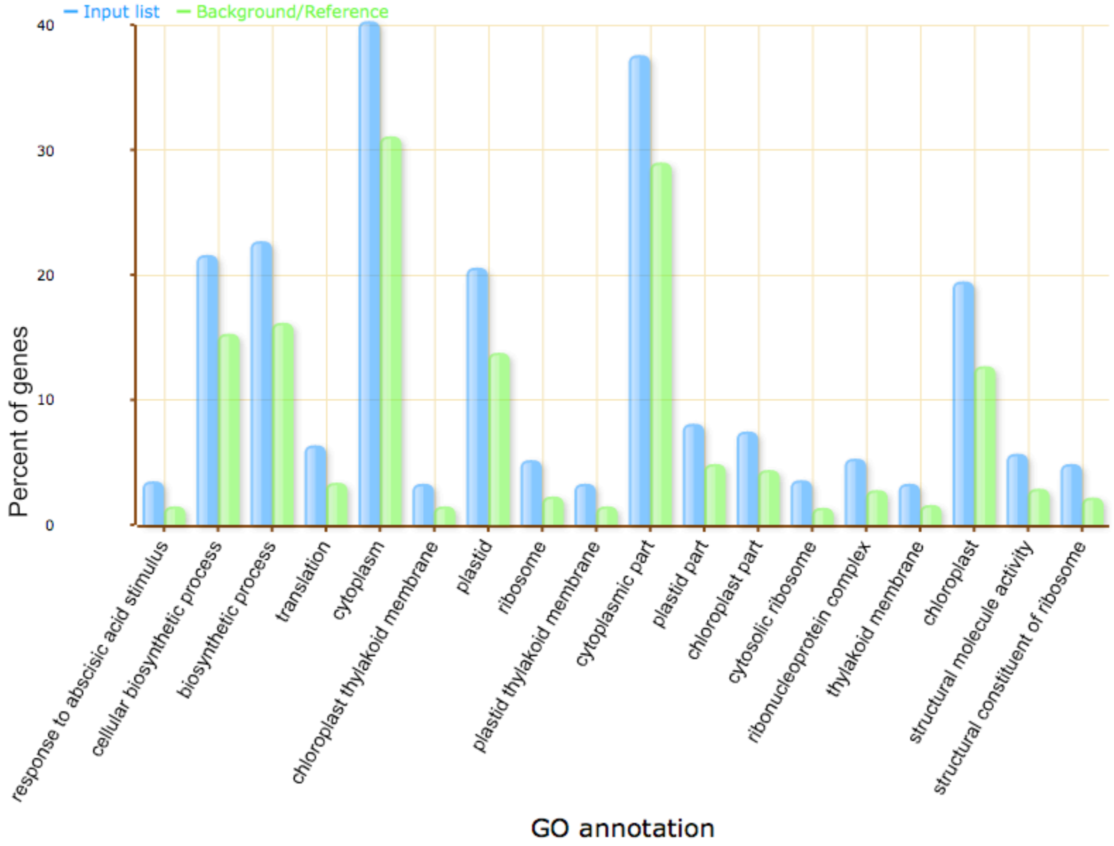

Supplementary Figure 1 | GO terms overrepresented in the list of genes differentially expressed in the second microarray experiment comparing E+ and E- treated cacao leaves. Input list refers to the list of genes differentially expressed and background list to all the genes in the array.

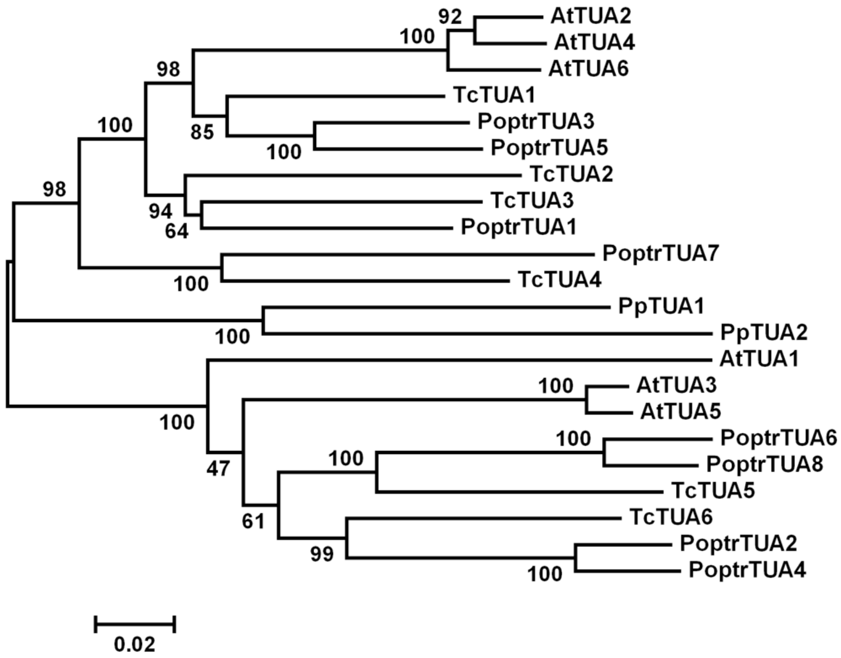

Supplementary Figure 2. Neighbor-Joining phylogenetic tree based on analysis of full-length α-tubulin coding sequences of *T. cacao* (TcTUA), *Arabidopsis* (AtTUA), and *Populus* (PoptrTUA). *Physcomitrella patens* (PpTUA) sequences were used as outgroup. Bootstrap values indicated next to each node.

Supplementary Table 1. Primer and probe sequences used for RT-qPCR TaqMan® assays.

Oligo Sequence ID	Primer Sequences	Probe Sequences
1562/ Tc09g034100	5'-CTCATTGCAGTGGTATTGGTTTTT-3'<br>5'-CGATGAGCATGAAGCTGCA-3'	5'-ACCAGACAGACACGC GGAAGGCC-3'
1852/ Tc00g042540	5'-CCAGAAATTGTCGTTCAAAGAGG-3'<br>5'-TGCCATCGGCATTTGAAA-3'	5'-CCGACGACGATGGTCTTCCGTGA-3'
1202/ Tc08g012560	5'-TGACTGGCGGAGGAGTGG -3'<br>5'-CGGAAATCATGGCCTGGA-3'	5'-CCGCAGTGTGGAGTGTACAGGGAGC-3'
3372/Tc01g013 950	5'-TGGAGGTTGATGACCAAAATTTACT-3'<br>5'-TGCAGAGATTAAGTCATTTGAGCAA-3	5'-TGGTGAACGCTCTACCCAGTGTTGGA-3'
1844/ Tc05g027250	5'-GGTGTTAGACCTCGAAACTGCAT-3'<br>5'-GTCGTCACAATCCACAACCTTATAGT-3'	5'-TTGACACCAATGGGAACGGCATCA-3'
1625/ Tc05g023720	5'-CGTCTCCCCGATATATACCTCTACTA-3'<br>5'-GTCGTCACAATCCACAACCTTATAGT-3'	5'-TGGGTGTATGGGATTGATGTGAAAGC-3'
2650/ Tc00g042540	5'-TGCCTTCACATCAAAATCATATTTC-3'<br>5'-CGGAGTTCATCACCATCACTATCA-3'	5'-TAGCCAGAGCTGATGAATCCCCGGT-3'
3107/ Tc00g042540	5'-CCAAAAGCCGGCAATGTATT-3'<br>5'-ACAAAGCTGGGCTGTGAAGAC-3'	5'-TTGCTTGAGGGTGGAGGTGCTTAAAGG-3'
1582	5'-TCCTGGGAGTGGTGGTTCTC-3'<br>5'-GGTTTCCCATAGCCGGGTAA-3'	5'-CCACTCCCTCCCTTGGTACCTACGA-3'
1541/ Tc01g015010	5'-TTCCTTTCTGCTTCATTACTACACAAC-3'<br>5'-TGAGCATCTTAGAAACACCTGCA-3'	5'-CAATGGTGCTTGTCAGCGGCAAGAA-3'
TcUBQ/ Tc02g024050	5'-GGGAATTCGATTAAGCAGTGGTA-3'<br>5'-CAGAGAAATTAAACCACTCGGGA-3'	5'-CGAGAGCAGCGACACCATCGACA-3'
TcACT/ Tc01g010900	5'-GCAGACCGAATGAGCAAGGA-3'<br>5'-GTGGTGCAACGACCTTAATCTTC-3'	5'-TCACTGCCCTTGCTCCAAGCAC-3'

Supplementary Table 2. Accession number of DNA sequences used in the phylogenetic analysis of tubulin genes.

Gene	Accession No.	Database
AtTUA1	AT1G64740	TAIR
AtTUA2	AT1G50010	TAIR
AtTUA3	AT5G19770	TAIR
AtTUA4	AT1G04820	TAIR
AtTUA5	AT5G19780	TAIR
AtTUA6	AT4G14960	TAIR
PoptrTUA1	Potri.002G111900	Phytozome
PoptrTUA2	Potri.003G220300	Phytozome
PoptrTUA3	Potri.001G289500	Phytozome
PoptrTUA4	Potri.001G004600	Phytozome
PoptrTUA5	Potri.009G085100	Phytozome
PoptrTUA6	Potri.013G056800	Phytozome
PoptrTUA7	Potri.017G081000	Phytozome
PoptrTUA8	Potri.019G036000	Phytozome
TcTUA1	Tc09g014070	CocoaGen DB
TcTUA2	Tc01g007190	CocoaGen DB
TcTUA3	Tc08g006650	CocoaGen DB
TcTUA4	Tc00g013710	CocoaGen DB
TcTUA5	Tc05g024170	CocoaGen DB
TcTUA6	Tc09g020300	CocoaGen DB
PpTUA1	AB096718.1	CocoaGen DB
PpTUA2	AB096719.1	CocoaGen DB

Supplementary Table 3. ***Theobroma cacao*** **genes up-regulated in leaves inoculated with endophyte** ***C. tropicale*** **(E+) at 14 days post inoculation compared to control E- leaves (1st microarray experiment).**

Oligo ID (unigene)	Tc Identifier	At Identifier	logFC	adj.P.Val (BH)	Annotation
1562	Tc09g034100	AT5G22920	2.85	7.89E-05	Putative RING finger and CHY zinc finger domain-containing protein 1
1541	Tc01g015010	N/A	2.49	0.006425932	Hypothetical protein
3107	Tc00g042540	N/A	2.37	0.0089428	21 kDa seed protein
607	Tc00g032450	N/A	2.36	0.000831026	Hypothetical protein
3372	Tc01g013950	N/A	2.34	7.89E-05	Small heat-shock protein, putative
1852	Tc00g042540	N/A	2.32	0.005205315	21 kDa seed protein
2650	Tc00g042540	AT1G17860	2.04	0.004081327	21 kDa seed protein
2678	Tc09g029650	AT5G10240	1.99	0.004806639	Asparagine synthetase [glutamine-hydrolyzing]
1625	Tc05g023720	N/A	1.94	0.001086555	Hypothetical protein
3286	Tc08g012560	AT1G77120	1.85	0.006971637	Alcohol dehydrogenase class-P
1202	Tc08g012560	AT1G77120	1.79	0.003356512	Alcohol dehydrogenase class-P
2167	Tc08g012560	AT1G77120	1.74	0.002748598	Alcohol dehydrogenase class-P
945	Tc03g029830	AT1G19270	1.68	0.00072612	Putative protein of unknown function
3125	Tc08g015310	AT1G35420	1.59	0.000674132	Putative Carboxymethylenebutenolidase homolog
3578	Tc09g030280	AT2G29630	1.53	0.000139484	Phosphomethylpyrimidine synthase
67	Tc00g049230	AT5G42965	1.48	0.00072612	Putative ribosomal recycling factor
3395	Tc06g018080	AT4G05180	1.44	0.003516622	Oxygen-evolving enhancer protein 3-2, chloroplastic
1276	Tc09g012670	N/A	1.44	0.007359855	Hypothetical protein
1241	Tc06g015510	AT1G28330	1.42	0.00667688	Auxin-repressed 12.5 kDa protein
3017	Tc09g035400	AT5G58260	1.41	0.006737283	Putative NAD(P)H-quinone oxidoreductase subunit N
3692	Tc09g021240	AT3G32930	1.41	0.000485805	Putative uncharacterized protein
1539	Tc08g004960	AT2G30600	1.40	0.003380419	Predicted protein
1623	Tc01g027810	AT1G03130	1.40	0.008595476	Photosystem I reaction center subunit II, chloroplastic
2706	Tc10g006930	N/A	1.34	0.000796656	Putative uncharacterized protein Sb01g043440
2399	Tc03g003190	N/A	1.24	0.003643057	Putative uncharacterized protein
1633	Tc06g018080	AT4G05180	1.23	0.007086348	Oxygen-evolving enhancer protein 3-2, chloroplastic
2228	Tc01g040840	N/A	1.22	0.000846934	Zinc finger A20 and AN1 domain-containing stress-associated protein 4
53	Tc06g018680	AT5G54770	1.20	0.000214127	Thiazole biosynthetic enzyme, chloroplastic
826	Tc05g008920	AT3G52750	1.18	0.000817763	Cell division protein ftsZ

1130	Tc09g005490	N/A	1.17	0.00196756	Photosystem II reaction center X protein
3174	Tc10g005310	AT3G54890	1.17	0.003380419	Chlorophyll a-b binding protein 6A, chloroplastic
3234	Tc05g002600	AT5G01530	1.16	0.00755627	Chlorophyll a-b binding protein CP29.2, chloroplastic
2620	Tc09g004470	AT4G29190	1.15	0.001534282	Zinc finger CCCH domain-containing protein 20
2221	Tc02g026660	AT5G43230	1.14	0.000817763	Putative uncharacterized protein
361	Tc01g035120	AT2G46210	1.14	0.00218019	Putative Fatty acid desaturase 3
814	Tc10g005040	AT3G52290	1.10	0.002185813	Putative Protein IQ-DOMAIN 1
1926	Tc01g027210	N/A	1.10	0.008815784	Hypothetical protein
2210	Tc05g024980	AT1G08570	1.10	0.000884846	Thioredoxin-like 1
1836	Tc09g014070	AT1G04820	1.10	0.005961964	Tubulin alpha-2 chain
2696	Tc10g001740	AT3G51880	1.10	0.002773635	Putative uncharacterized protein
3043	Tc01g036150	AT3G61310	1.04	0.000796656	AT-hook DNA-binding protein
616	Tc02g025760	AT1G67740	1.03	0.001734709	Photosystem II core complex proteins psbY, chloroplastic
3454	Tc01g035110	AT2G46220	1.00	0.001957463	Putative uncharacterized protein
2038	Tc10g000750	N/A	0.99	0.000971308	Putative Polyadenylate-binding protein-interacting protein 2
1558	Tc03g005310	N/A	0.98	0.000713822	Putative uncharacterized protein
1463	Tc00g012130	AT3G26740	0.97	0.001534282	Light-regulated protein, putative
1144	Tc03g009540	AT5G65310	0.96	0.005917735	Putative uncharacterized protein
1223	Tc03g001980	N/A	0.96	0.0018252	Oleosin (Fragment)
1345	Tc07g004790	AT5G54770	0.95	0.000713822	Thiazole biosynthetic enzyme, chloroplastic
19	Tc10g015490	AT1G04940	0.94	0.008510902	Putative Uncharacterized protein ycf60
1778	Tc00g046750	AT1G67090	0.93	0.003091334	Ribulose bisphosphate carboxylase small chain, chloroplastic
2540	Tc03g013820	AT5G50250	0.93	0.005010586	31 kDa ribonucleoprotein, chloroplastic
1731	Tc01g018570	AT2G05070	0.93	0.002928239	Chlorophyll a-b binding protein 151, chloroplastic
2647	Tc03g009540	AT5G65310	0.90	0.007345518	Putative uncharacterized protein
1934	Tc04g028340	N/A	0.89	0.005503291	Late embryogenesis abundant protein Lea5-D
1553	Tc03g008290	AT3G47470	0.88	0.002928239	Chlorophyll a-b binding protein 4, chloroplastic
1216	Tc01g035310	AT3G61540	0.86	0.003034147	Putative Proline iminopeptidase
1786	Tc05g024170	AT5G19780	0.85	0.004415328	Tubulin alpha-3 chain
584	Tc03g019840	N/A	0.84	0.007345518	Putative uncharacterized protein
1481	Tc05g014020	AT3G56680	0.82	0.003284157	Predicted protein
1714	Tc01g013570	AT3G58610	0.81	0.002773635	Ketol-acid reductoisomerase, chloroplastic
1511	Tc03g021770	AT5G24490	0.81	0.004092315	30S ribosomal protein 1, chloroplastic
3384	Tc01g031680	AT2G47400	0.81	0.00156296	Calvin cycle protein CP12
3597	Tc07g003890	N/A	0.80	0.001734709	Putative Protein synthesis inhibitor II

1720	Tc09g014070	AT1G04820	0.78	0.006524282	Tubulin alpha-2 chain
2317	Tc07g004790	AT5G54770	0.78	0.003250399	Thiazole biosynthetic enzyme, chloroplastic
1520	Tc07g004790	AT5G54770	0.78	0.003250399	Thiazole biosynthetic enzyme, chloroplastic
3800	Tc01g033920	AT5G22400	0.79	0.00530225	Predicted protein
2719	Tc08g003550	N/A	0.77	0.009413429	Putative Predicted protein
3648	Tc05g030320	AT1G56220	0.77	0.000971308	Putative uncharacterized protein
1478	Tc08g011150	AT1G21750	0.77	0.004587037	Protein disulfide-isomerase
1784	Tc09g028910	N/A	0.74	0.001957463	Histone deacetylase HDT1
1071	Tc06g010450	AT1G72416	0.73	0.00218019	Chaperone protein dnaJ, putative
1239	Tc01g013560	AT3G58610	0.73	0.002769226	Ketol-acid reductoisomerase, chloroplastic
3108	Tc09g029720	AT2G29990	0.73	0.007291724	Putative Probable NADH dehydrogenase
2402	Tc04g019410	AT3G48560	0.73	0.002897274	Acetolactate synthase 2, chloroplastic
1240	Tc04g028380	AT4G15930	0.72	0.001628176	Dynein light chain 1, cytoplasmic
1138	Tc01g006300	AT3G49940	0.72	0.001734709	LOB domain-containing protein 38
1143	Tc04g005120	AT2G31410	0.71	0.0018252	Putative uncharacterized protein
1849	Tc04g004710	N/A	0.71	0.00218019	Putative Shikimate kinase, chloroplastic
2493	Tc02g008320	AT1G49510	0.71	0.001440213	Putative uncharacterized protein
2920	Tc02g031250	AT2G04030	0.69	0.008602806	Putative Heat shock cognate 90 kDa protein
2262	Tc09g029700	AT1G07170	0.69	0.005698411	Uncharacterized protein At1g07170/At2g30000
3287	Tc09g030140	AT2G29690	0.69	0.006425932	Anthranilate synthase component I-2, chloroplastic
2861	Tc06g015510	AT1G28330	0.67	0.002088907	Auxin-repressed 12.5 kDa protein
176	Tc08g001700	AT1G75060	0.67	0.000846934	Putative uncharacterized protein
1588	Tc01g020040	AT3G25410	0.65	0.007345518	Putative Uncharacterized sodium-dependent transporter yocS
2992	Tc09g028910	N/A	0.64	0.00858536	Histone deacetylase HDT1
24	Tc09g023530	AT3G10330	0.63	0.006063126	Transcription initiation factor IIB
1465	Tc04g005190	AT3G26720	0.63	0.005695233	Putative Lysosomal alpha-mannosidase
2874	Tc09g014070	AT1G04820	0.63	0.008882262	Tubulin alpha-2 chain
3499	Tc09g005650	N/A	0.62	0.0018252	Putative uncharacterized protein
3605	Tc00g046760	AT4G30200	0.61	0.00771255	Putative Protein VERNALIZATION INSENSITIVE 3
3200	Tc08g000460	AT1G07700	0.60	0.003380419	Predicted protein
3778	Tc00g004350	AT4G25990	0.59	0.004323393	Putative Protein CHLOROPLAST IMPORT APPARATUS 2
1403	Tc05g002870	AT5G01210	0.59	0.007825986	Putative BAHD acyltransferase DCR
1898	Tc03g026940	AT1G63180	0.58	0.00419029	UDP-glucose 4-epimerase 1
2524	Tc05g006010	AT1G07920	0.58	0.009252036	Elongation factor 1-alpha
3490	Tc03g019670	AT5G52100	0.58	0.007825986	Putative dihydrodipicolinate reductase 3, chloroplastic
3625	Tc09g003510	AT5G11480	0.58	0.005961964	GTP-binding protein At2g22870

1902	Tc01g020090	N/A	0.57	0.003250399	Putative Lipoxygenase 2.1, chloroplastic
3527	Tc09g004460	AT3G53920	0.55	0.009288043	Putative uncharacterized protein
1195	Tc05g031090	AT1G56350	0.55	0.006971637	Putative Peptide chain release factor 2
2596	Tc02g001650	AT1G09810	0.54	0.003282426	Yth domain containing protein, putative
1758	Tc01g019260	AT2G04270	0.53	0.007572834	Predicted protein (Fragment)
2698	Tc10g016300	AT5G05280	0.53	0.002088907	RING-H2 finger protein ATL3C
1773	Tc06g016300	N/A	0.53	0.008649098	Polyphenol oxidase, chloroplastic
2863	Tc05g004890	AT5G02530	0.52	0.001734709	Putative RNA and export factor-binding protein 2
3599	Tc04g004710	N/A	0.51	0.00351393	Putative Shikimate kinase, chloroplastic
3208	Tc05g024180	AT5G18100	0.49	0.007825986	Superoxide dismutase [Cu-Zn] 2
1362	Tc04g028210	AT3G16640	0.48	0.005272515	Translationally-controlled tumor protein homolog
2878	Tc02g019690	AT5G62000	0.48	0.006738415	Auxin response factor 2
1689	Tc02g008880	N/A	0.47	0.005205315	Thioredoxin M-type 3, chloroplastic
1245	Tc09g023530	AT3G10330	0.46	0.005961964	Transcription initiation factor IIB
3023	Tc02g027360	AT1G70985	0.46	0.008882262	Predicted protein
343	Tc04g029230	AT4G16155	0.45	0.005961964	Dihydrolipoyl dehydrogenase
2334	Tc02g032880	AT1G14060	0.45	0.005969817	Hypothetical protein
3590	Tc02g013800	AT5G08280	0.41	0.005408968	Porphobilinogen deaminase, chloroplastic
3429	Tc01g029430	AT4G03150	0.41	0.0089428	Predicted protein
2115	Tc00g038640	AT1G07820	0.40	0.006971637	Histone H4
2530	Tc07g012930	AT1G17200	0.40	0.009288043	UPF0497 membrane protein f16
2533	Tc00g000920	N/A	0.40	0.009228584	Putative uncharacterized protein

Supplementary Table 4. ***Theobroma cacao*** **genes down-regulated in leaves inoculated with endophyte** ***C. tropicale*** **(E+) at 14 days post inoculation compared to control E- leaves (1st microarray experiment).**

Oligo ID (unigene)	Tc Identifier	At Accession	logFC	adj.P.Val (BH)	Annotation
1654	Tc04g024360	AT4G14690	-3.36	1.24E-05	Early light-induced protein, chloroplastic
1031	Tc01g033560	AT1G01060	-3.01	1.24E-05	Putative Protein LHY
1861	Tc04g024360	AT4G14690	-2.91	4.77E-05	Early light-induced protein, chloroplastic
2311	Tc10g016510	AT3G56290	-2.91	0.001020321	Predicted protein
2836	Tc02g014240	N/A	-2.88	4.77E-05	Predicted protein
355	Tc04g021580	AT3G24190	-2.78	0.000223391	Protein ABC1, mitochondrial, putative
3184	Tc03g018090	AT5G51440	-2.28	0.001683468	23.6 kDa heat shock protein, mitochondrial
3182	Tc02g009700	AT2G15020	-2.15	0.000295206	Putative uncharacterized protein
3413	Tc04g017020	N/A	-2.06	0.001686281	Putative Uncharacterized UDP-glucosyltransferase At1g05670
1446	Tc10g002920	AT2G39730	-2.02	0.000817763	Ribulose bisphosphate carboxylase/oxygenase activase 1, chloroplastic
236	Tc09g030680	AT1G53540	-2.01	0.002769226	18.5 kDa class I heat shock protein
1410	Tc05g005680	AT5G02780	-1.99	0.00072612	Protein IN2-1 homolog B
3532	Tc10g002920	AT2G39730	-1.98	0.001833667	Ribulose bisphosphate carboxylase/oxygenase activase 1, chloroplastic
3411	Tc06g006590	N/A	-1.82	0.001430698	N/A
1673	Tc09g030680	AT1G53540	-1.77	0.00218019	18.5 kDa class I heat shock protein
1430	Tc10g002920	AT2G39730	-1.70	0.00072612	Ribulose bisphosphate carboxylase/oxygenase activase 1, chloroplastic
271	Tc09g005310	N/A	-1.64	0.00871947	17.9 kDa class II heat shock protein
1678	Tc10g002920	AT2G39730	-1.56	0.001734709	Ribulose bisphosphate carboxylase/oxygenase activase 1, chloroplastic
1095	Tc01g026990	AT3G54660	-1.56	0.00137409	Glutathione reductase, chloroplastic (Fragment)
1826	Tc04g021630	AT5G13930	-1.54	0.002520431	Chalcone synthase 2
1516	Tc04g021630	AT5G13930	-1.53	0.00218019	Chalcone synthase 2
1261	Tc07g007210	AT5G54250	-1.44	0.004353544	Cyclic nucleotide-gated ion channel 4
3617	Tc00g025880	N/A	-1.43	0.003091334	Putative uncharacterized protein
1217	Tc04g021630	N/A	-1.40	0.003486062	N/A
1512	Tc01g026990	AT3G54660	-1.37	0.002928239	Glutathione reductase, chloroplastic (Fragment)
1513	Tc06g019310	AT4G04610	-1.35	0.002541862	5'-adenylylsulfate reductase 3, chloroplastic
1213	Tc00g057520	AT3G54500	-1.34	0.001734709	Putative uncharacterized protein
1658	Tc08g011380	AT1G44575	-1.34	0.005010586	Photosystem II 22 kDa protein, chloroplastic

3374	Tc09g029150	AT5G22830	-1.25	0.002366383	At5g22830
1109	Tc07g003570	N/A	-1.24	0.003034147	1-acyl-sn-glycerol-3-phosphate acyltransferase
1426	Tc04g020060	AT3G23660	-1.23	0.001734709	Putative Protein transport protein SEC23
3748	Tc08g011220	AT5G54160	-1.21	0.000713822	Caffeic acid 3-O-methyltransferase
3390	Tc00g015640	AT1G75270	-1.20	0.006776216	Glutathione S-transferase DHAR1, mitochondrial
3393	Tc01g010150	AT5G64840	-1.19	0.002165356	ABC transporter F family member 5
2307	Tc03g017540	AT5G24120	-1.18	0.002773635	RNA polymerase sigma factor rpoD, putative
2770	Tc07g004100	AT4G27030	-1.16	0.003785171	Predicted protein
1327	Tc10g003310	AT3G53750	-1.16	0.000680547	Actin
1386	Tc02g028050	AT1G70700	-1.12	0.006553136	Putative Protein TIFY 6B
1270	Tc09g004830	AT4G33010	-1.07	0.008874615	Glycine dehydrogenase [decarboxylating], mitochondrial
10	Tc03g020100	AT5G23950	-1.06	0.001734709	Putative uncharacterized protein
1959	Tc09g033010	AT3G09640	-1.04	0.00889239	L-ascorbate peroxidase 2, cytosolic
1377	Tc04g026150	AT4G26740	-1.02	0.004904226	Caleosin
1579	Tc08g005260	AT1G42970	-1.02	0.003091334	Glyceraldehyde-3-phosphate dehydrogenase B, chloroplastic
2539	Tc03g022890	AT1G64660	-1.00	0.007345518	Putative Methionine gamma-lyase
3231	Tc02g006400	AT4G38970	-0.98	0.00137409	Probable fructose-bisphosphate aldolase 1, chloroplastic
3661	Tc03g023580	AT2G44530	-0.97	0.002928239	Ribose-phosphate pyrophosphokinase 1
1568	Tc09g005980	AT3G43540	-0.97	0.006524282	Putative uncharacterized protein
1097	Tc08g007740	AT4G30020	-0.95	0.002773635	Subtilisin-like protease
1331	Tc09g013970	AT4G34350	-0.93	0.008420192	4-hydroxy-3-methylbut-2-enyl diphosphate reductase
2543	Tc00g057520	AT3G54500	-0.92	0.003246957	Putative uncharacterized protein
1896	Tc05g002470	AT2G38230	-0.90	0.003400087	Probable pyridoxal biosynthesis protein PDX1
3086	Tc03g017540	AT5G24120	-0.90	0.005698411	RNA polymerase sigma factor rpoD, putative
3564	Tc03g017400	N/A	-0.90	0.000846934	Protein phosphatase 2c, putative
1167	Tc04g002700	AT3G04120	-0.90	0.001734709	Glyceraldehyde-3-phosphate dehydrogenase, cytosolic
1892	Tc09g033010	AT3G09640	-0.89	0.006244458	L-ascorbate peroxidase 2, cytosolic
2079	Tc00g017110	AT4G28150	-0.89	0.005961964	Hypothetical protein
3731	Tc05g012720	AT5G36790	-0.89	0.008882262	Putative Phosphoglycolate phosphatase
836	Tc01g039090	AT1G05940	-0.89	0.001534282	Putative Uncharacterized amino acid permease yfnA

1360	Tc07g011890	N/A	-0.87	0.002098809	Secologanin synthase
2703	Tc10g001840	AT4G00430	-0.87	0.005486819	Probable aquaporin PIP-type 7a
2556	Tc08g002400	AT5G66460	-0.86	0.006971637	Mannan endo-1,4-beta-mannosidase 7
1645	Tc04g030020	AT3G06520	-0.85	0.001109342	RNA binding protein, putative
3251	Tc02g010870	AT5G09410	-0.81	0.000992942	Calmodulin-binding transcription activator 2
2376	Tc00g057520	AT3G54500	-0.79	0.002847057	Putative uncharacterized protein
3356	Tc01g015210	AT1G06570	-0.79	0.007240928	4-hydroxyphenylpyruvate dioxygenase
56	Tc01g034650	AT4G01050	-0.78	0.003516622	Uncharacterized protein At4g01050
1636	Tc06g006590	AT5G25430	-0.78	0.0018252	Boron transporter 4
1370	Tc04g024330	AT4G14716	-0.78	0.002243099	1,2-dihydroxy-3-keto-5-methylthiopentene dioxygenase 3
1521	Tc02g032180	AT4G09350	-0.76	0.005062945	Predicted protein
1567	Tc08g010270	N/A	-0.76	0.005465855	Flavonol synthase/flavanone 3-hydroxylase
2046	Tc10g001840	AT4G00430	-0.75	0.0010706	Probable aquaporin PIP-type 7a
3523	Tc06g011440	AT4G19640	-0.77	0.006738415	Ras-related protein RHA1
1194	Tc01g002990	AT4G36530	-0.73	0.00419029	Putative Uncharacterized hydrolase yugF
1295	Tc08g002160	AT2G16280	-0.73	0.002165356	3-ketoacyl-CoA synthase 4
1237	Tc09g032550	AT1G07810	-0.72	0.004353544	Calcium-transporting ATPase 4, endoplasmic reticulum-type
1514	Tc01g016790	AT2G42975	-0.71	0.006063126	Putative uncharacterized protein
2166	Tc01g035920	AT1G01660	-0.71	0.003091334	U-box domain-containing protein 33
3496	Tc01g003080	AT4G36550	-0.71	0.008920999	Putative U-box domain-containing protein 5
2407	Tc08g003020	AT2G32450	-0.69	0.005893556	Uncharacterized TPR repeat-containing protein At1g05150
1450	Tc09g009310	AT1G65660	-0.68	0.008882262	Hypothetical protein
332	Tc00g003520	AT1G51200	-0.68	0.003284157	Zinc finger A20 and AN1 domain-containing stress-associated protein 8
1369	Tc01g031500	AT3G62580	-0.66	0.005010586	Putative Transmembrane protein 205
3543	Tc01g009900	AT5G64940	-0.66	0.005010586	Putative Uncharacterized protein sll1770
360	Tc05g030470	AT5G27860	-0.65	0.006425932	Hypothetical protein
1725	Tc05g010340	AT5G03630	-0.65	0.00218019	Monodehydroascorbate reductase
3100	Tc09g015300	AT4G28060	-0.64	0.002510526	Cytochrome c oxidase subunit 6B
2409	Tc06g012490	AT1G30120	-0.63	0.000992942	Pyruvate dehydrogenase E1 component subunit beta
2279	Tc10g003700	AT3G46010	-0.63	0.001734709	Actin-depolymerizing factor 2
3378	Tc03g006290	AT3G10113	-0.62	0.003284157	Predicted protein

1688	Tc00g092420	AT3G63410	-0.61	0.008119273	37 kDa inner envelope membrane protein, chloroplastic
3690	Tc02g007500	AT5G48420	-0.60	0.006776216	Predicted protein
1570	Tc07g016590	AT1G79550	-0.59	0.001924809	Phosphoglycerate kinase, cytosolic
1339	Tc04g030750	N/A	-0.58	0.003034147	Putative uncharacterized protein
1045	Tc03g023580	AT2G44530	-0.58	0.003380419	Ribose-phosphate pyrophosphokinase 1
2053	Tc08g002400	AT5G66460	-0.54	0.00419029	Mannan endo-1,4-beta-mannosidase 7
3622	Tc08g005000	AT4G35270	-0.54	0.005079809	Predicted protein
1628	Tc09g034380	AT2G30110	-0.52	0.005961964	Ubiquitin-activating enzyme E1 2
3081	Tc05g014300	AT5G04140	-0.50	0.005010586	Ferredoxin-dependent glutamate synthase, chloroplastic
1891	Tc09g017350	AT4G30210	-0.49	0.008649098	NADPH--cytochrome P450 reductase
1886	Tc00g037200	AT1G59900	-0.47	0.009402697	Pyruvate dehydrogenase E1 component subunit alpha-1, mitochondrial
1358	Tc08g003070	AT1G19910	-0.35	0.009413429	V-type proton ATPase 16 kDa proteolipid subunit

Supplementary Table 5. RT-qPCR analysis of 10 *T. cacao* genes regulated by endophyte *C. tropicale* in cacao leaf tissue.

Oligo ID/ Tc identifier	Annotation	Expression (Fold Change) E+ relative to E- leaves	P-value
1562/ Tc09g034100	Putative RING finger and CHY zinc finger domain-containing protein 1	53.162	0
1844/ Tc05g027250	Pathogenesis-related protein P2	24.381	0.001
3372/ Tc01g013950	Small heat-shock protein, putative	17.348	0
1852/ Tc00g042540	21 kDa seed protein	11.372	0.021
2650/ Tc00g042540	21 kDa seed protein	7.09	0.015
3107/ Tc00g042540	21 kDa seed protein	6.941	0.005
1541/ Tc01g015010	Hypothetical protein	6.462	0
1625/ Tc05g023720	Hypothetical protein	3.189	0.001
1582/ Tc04:20645013..20645348	Repetitive proline-rich cell wall protein	3.186	0.005
1202/ Tc08g012560	Alcohol dehydrogenase class-P	2.981	0.019
Tc01g010900	Actin 7	1.189	0.619
Tc02g024050	Ubiquitin	0.841	0.463

Ten unigenes were selected for QPCR analysis: 8 unigenes (representing 6 genes) were significantly up-regulated in all six biological replicate samples and 2 unigenes (oligo IDs 1844 and 1582) were up-regulated in only four biological replicates. *TcActin7* and *TcUbiquitin* housekeeping genes were used as references for data normalization. Data normalization, efficiency correction, statistical analysis and relative treated/control expression ratios (fold differences) were conducted as described in methods.

Supplementary Table 6. ***T. cacao*** **genes up-regulated in leaves inoculated with endophyte** ***C. tropicale*** **(E+) at 3 days post inoculation compared to control E- leaves (2nd microarray experiment).**

Oligo ID (unigene)	Tc Identifier	At Identifier	logFC	adj.P.Val (BH)	Annotation
KBA1YL18FM1_	Tc00g053030	#N/A	4.47	0.000166852	Hypothetical protein
KBB16YN05FM1_gi_110743100_dbj_BAE99442_1_	Tc05g005250	AT5G02580	4.05	0.00049603	Putative uncharacterized protein
KCAE6YJ18FM1_	Tc08g001050	#N/A	3.15	0.020296385	Putative Early nodulin-like protein 2
LITE_CONTIG_2591	Tc05g023660	#N/A	3.02	0.043305172	Predicted protein
KBB11YG18FM1_	Tc07g001580	#N/A	2.91	0.000166852	Cytochrome P450 71D10
LITE_CONTIG_6237	Tc03g025670	AT4G12390	2.38	0.017171383	21 kDa protein
KBF4YL22FM1_Lotus	Tc06g007000	#N/A	2.27	0.034056614	Inositol oxygenase 1
LITE_CONTIG_6417	Tc07g007300	AT4G10270	2.21	0.00194669	Putative uncharacterized protein
KCAA9YP17FM1_	Tc03g013020	#N/A	2.19	0.015725857	Putative Disease resistance response protein 206
KAP1YB09_hypothetical	Tc06g013930	AT1G29290	2.16	0.019604988	Predicted protein
KCAA2YN23FM1_hypothetical	Tc08g005400	AT1G72200	2.10	0.002538273	RING-H2 finger protein ATL1N
LITE_CONTIG_2777	Tc02g002750	#N/A	2.09	0.014853248	Putative uncharacterized protein
KBB11YM19FM1_	Tc02g008130	#N/A	2.06	0.001205893	Putative B3 domain-containing protein Os04g0581400
LKBE2YM10FM2_	Tc01g006470	#N/A	2.03	0.009335489	Calcium-transporting ATPase 2, plasma membrane-type
LITE_CONTIG_6497	Tc02g015010	AT4G10270	2.01	0.000898366	Predicted protein
KAT2YG13FM1_hypothetical	Tc08g012130	AT4G19950	2.00	0.009072556	Predicted protein
LITE_CONTIG_303	Tc02g009130	#N/A	2.00	0.034917595	Anthocyanin 3'-O-beta-glucosyltransferase
KBF10YK18FM1_putative	Tc03g019360	AT5G51990	1.99	0.016049731	Dehydration-responsive element-binding protein 1D
KBB4YM04FM1_Zinc	Tc01g030430	AT1G02860	1.98	0.002224521	E3 ubiquitin-protein ligase BAH1
LITE_CONTIG_6831	Tc03g003050	AT1G68320	1.93	0.013949631	Predicted protein
KCL4YK18FM1_Protein	Tc09g008520	AT4G26260	1.92	0.005539549	Inositol oxygenase 2
227368416_m05677	Tc09g034330	AT3G51770	1.90	0.000685043	Ethylene-overproduction protein 1
KBA10YN11FM1_hypothetical	Tc00g059570	AT1G55740	1.83	0.031078681	Probable galactinol--sucrose galactosyltransferase 1
LITE_CONTIG_807	Tc00g041460	AT4G28730	1.77	0.03723342	Probable glutathione S-transferase
LITE_CONTIG_6389	Tc01g006440	AT5G67480	1.74	0.001205893	Putative uncharacterized protein (Fragment)
LITE_CONTIG_2149	Tc01g032490	AT5G26751	1.74	0.001093078	Glycogen synthase kinase-3 homolog MsK-3

LITE_CONTIG_2701	Tc03g004830	AT3G18080	1.73	0.001396423	Beta-glucosidase 44
KBF1YK10RM1_syringolide_induced	Tc09g016910	AT4G25810	1.70	0.047917363	Xyloglucan endotransglucosylase/hydrolase protein 22
1562_68418_m02680	Tc09g034100	AT5G22920	1.69	0.003677679	Putative RING finger and CHY zinc finger domain-containing protein 1
LITE_CONTIG_6290	Tc08g012550	AT1G43800	1.69	0.019604988	Acyl-[acyl-carrier-protein] desaturase, chloroplastic
CL311Contig1_gi_23297392_gb_AAN12959_1_	Tc07g009490	AT1G78830	1.65	0.034731082	Putative Epidermis-specific secreted glycoprotein EP1
KAS8YB15FM1_hypothetical	Tc07g009940	AT1G66910	1.64	0.010236471	Predicted protein
LITE_CONTIG_1892	Tc05g027320	AT3G04720	1.62	0.032520334	Pro-hevein
KBB13YJ10FM1_gi_46810683_gb_AAT01656_1_	Tc09g034330	AT3G51770	1.62	0.001268659	Ethylene-overproduction protein 1
LITE_CONTIG_6836	Tc03g016070	AT5G07330	1.60	0.000685043	Predicted protein
KCAE2YM20FM1_gi_110743057_dbj_BAE99421_1_	Tc05g025040	AT3G04070	1.60	0.01779356	NAC domain-containing protein 29
KAC2YM21FM1_hypothetical	Tc05g018920	AT3G57950	1.59	0.012251306	Predicted protein
LITE_CONTIG_6516	Tc02g015040	AT4G10270	1.59	0.012031415	Predicted protein
218268414_m05066	Tc01g002550	AT1G43910	1.57	0.007086693	ATP binding protein, putative
LITE_CONTIG_4578	Tc04g005150	#N/A	1.57	0.026618326	Light-regulated protein, putative
2129_68415_m01861	Tc08g002130	AT2G16250	1.57	0.006002845	Probable LRR receptor-like serine/threonine-protein kinase At2g16250
LITE_CONTIG_1256	Tc08g008820	AT4G08950	1.53	0.003470866	Predicted protein
LITE_CONTIG_6833	Tc02g010220	#N/A	1.53	0.003936679	Putative uncharacterized protein
LITE_CONTIG_3732	Tc07g006560	AT4G27450	1.52	0.000338912	Putative Stem-specific protein TSJT1
LITE_CONTIG_5706	Tc07g007270	AT4G10270	1.52	0.002802109	Putative uncharacterized protein
LITE_CONTIG_6504	Tc01g017710	AT1G05010	1.52	0.019848659	1-aminocyclopropane-1-carboxylate oxidase 1
KAQ10YP09FM1_	Tc01g002650	#N/A	1.52	0.001370523	Predicted protein (Fragment)
LITE_CONTIG_2821	Tc01g005710	#N/A	1.50	0.033358875	Transcription factor MYB44
LITE_CONTIG_4757	Tc09g006940	AT5G21940	1.49	0.000474427	Putative uncharacterized protein
LITE_CONTIG_4030	Tc10g003610	AT2G28760	1.49	0.004599223	UDP-glucuronic acid decarboxylase 1
KAS6YP19FM1_hypothetical	Tc00g051081	AT4G23160	1.47	0.027682578	Putative Retrovirus-related Pol polyprotein from transposon TNT 1-94
LITE_CONTIG_1580	Tc02g010600	AT2G22570	1.47	0.00049603	Putative Uncharacterized isochorismatase family protein pncA
LITE_CONTIG_1823	Tc04g027160	AT5G18840	1.46	0.007265786	Sugar transporter

					ERD6-like 16
LITE_CONTIG_1640	Tc04g018110	AT3G54420	1.45	0.014629966	Endochitinase PR4
KAA10YN05FM1_hypothetical	Tc04g004790	#N/A	1.45	0.001288092	Putative Zinc finger protein CONSTANS-LIKE 14
KAA1YG04FM1_	Tc05g025730	#N/A	1.43	0.007224288	Putative 3'-5' exoribonuclease CSL4 homolog
LITE_CONTIG_4146	Tc08g007210	AT1G58420	1.43	0.001828202	Predicted protein
KBB13YE23FM1_anthocyanin	Tc00g075710	AT1G03940	1.42	0.001125723	Putative Anthocyanin 5-aromatic acyltransferase
KCAE6YG07FM1_	Tc01g006440	#N/A	1.42	0.008609217	Putative uncharacterized protein (Fragment)
LITE_CONTIG_5270	Tc02g000010	AT3G12670	1.42	0.006953612	CTP synthase
KBF2YJ21FM1_nam_like	Tc01g012710	AT5G08790	1.41	0.032755686	NAC domain-containing protein 2
KBF3YL06FM1_	Tc02g004320	#N/A	1.40	0.001867512	Protein kinase, putative
LITE_CONTIG_1911	Tc02g024370	AT1G10760	1.39	0.019848659	Alpha-glucan water dikinase, chloroplastic
KAP24YD16FM1_hypothetical	Tc01g002650	AT2G18193	1.39	0.035490727	Predicted protein (Fragment)
LITE_CONTIG_4402	Tc01g006010	AT2G23150	1.39	0.033786148	Metal transporter Nramp3
CL121Contig1_	Tc02g030080	#N/A	1.39	0.008440203	Alpha,alpha-trehalose-phosphate synthase [UDP-forming] 6
KAV3YJ10FM1_hypothetical	Tc03g022450	AT2G18196	1.38	0.028756367	Putative Copper transport protein ATOX1 homolog
KBF1YF06FM1_	Tc09g033290	#N/A	1.37	0.007647574	SNF1-related protein kinase regulatory subunit beta-1
LITE_CONTIG_2522	Tc09g033020	AT3G45640	1.37	0.005197034	Mitogen-activated protein kinase 3
LITE_CONTIG_4094	Tc01g018340	AT1G12780	1.36	0.017610971	Putative uncharacterized protein (Fragment)
CL362Contig1_	Tc03g026900	#N/A	1.36	0.001548988	Heat stress transcription factor B-2b
KAV2YI08FM1_putative	Tc08g012560	AT1G77120	1.34	0.032238868	Alcohol dehydrogenase class-P
LITE_CONTIG_5972	Tc04g016450	AT5G59320	1.33	0.011448102	Non-specific lipid-transfer protein
LITE_CONTIG_5202	Tc06g013590	AT4G18930	1.32	0.028958138	Cyclic phosphodiesterase
LITE_CONTIG_6318	Tc08g012590	AT1G77120	1.32	0.001867512	Alcohol dehydrogenase class-P
KAA15YC22FM1_hypothetical	Tc01g007370	AT2G22680	1.32	0.00421182	Protein binding protein, putative
KBB14YN08FM1_hypothetical	Tc09g031520	AT5G13820	1.32	0.000716466	Putative Telomere-binding protein 1
LITE_CONTIG_6306	Tc03g029980	AT2G35520	1.32	0.003833486	Defender against cell death 1
KAA13YD17FM1_gi_30984526_gb_AAP42726_1_	Tc01g039040	AT3G60390	1.31	0.015991269	Homeobox-leucine zipper protein HAT3
LITE_CONTIG_2861	Tc08g011870	AT1G21400	1.30	0.015615563	2-oxoisovalerate dehydrogenase subunit alpha, mitochondrial
LITE_CONTIG_1204	Tc09g030850	AT3G53990	1.30	0.000716466	Putative Universal stress protein A-like protein
KAP4YD13FM1_	Tc05g008420	#N/A	1.30	0.004321031	PAN domain-containing

					protein At5g03700
LITE_CONTIG_2070	Tc09g009030	AT1G79750	1.29	0.002538273	NADP-dependent malic enzyme
294768416_m01121	Tc09g014710	AT3G12580	1.29	0.021549918	Heat shock 70 kDa protein
KAQ9YK21FM1_putative	Tc06g015310	AT3G08910	1.29	0.001332437	Putative DnaJ homolog subfamily B member 4
LITE_CONTIG_4899	Tc08g003750	AT4G24190	1.29	0.004599223	Endoplasmin homolog
KCAK1YA16FM1_hypothetical	Tc05g028690	#N/A	1.29	0.000524006	Putative Disease resistance protein At4g27190
KAS4YK01FM1_unknown	Tc02g033420	AT1G26850	1.28	0.000500135	Probable methyltransferase PMT2
KAC4YF16FM1_GroEL_like	Tc04g022140	AT2G33210	1.28	0.019251787	Chaperonin CPN60-2, mitochondrial
KAV12YE24FM1_gi_113564560_dbj_BAF14903_1_	Tc02g033290	AT3G28960	1.26	0.011131933	Amino acid transporter
LITE_CONTIG_584	Tc01g039950	AT2G44640	1.26	0.019928234	Putative uncharacterized protein
KCL5YN19FM1_unknown	Tc10g000780	AT4G29780	1.26	0.042663747	Predicted protein (Fragment)
LITE_CONTIG_4442	Tc01g028000	AT3G22550	1.25	0.001205893	Putative uncharacterized protein
KAS9YM12FM1_Vitis	Tc07g009900	#N/A	1.25	0.005722463	BES1/BZR1 homolog protein 4
KAQ10YJ01FM1_	Tc07g005270	#N/A	1.24	0.019604988	Chitin-inducible gibberellin-responsive protein 1
LITE_CONTIG_2988	Tc08g007720	#N/A	1.24	0.043616523	Putative Acyl-CoA-binding domain-containing protein 3
LITE_CONTIG_2999	Tc02g006210	AT5G10990	1.23	0.004599223	Putative Indole-3-acetic acid-induced protein ARG7
KAT6YN15FM1_gi_111074382_gb_ABH04564_1_	Tc00g014360	AT3G11340	1.23	0.028329439	Cytokinin-N-glucosyltransferase 1
LITE_CONTIG_4032	#N/A	#N/A	1.23	0.015986042	#N/A
LITE_CONTIG_2119	Tc01g008710	AT1G22360	1.23	0.029178215	Cytokinin-O-glucosyltransferase 2
LITE_CONTIG_6551	Tc03g020090	AT4G25630	1.22	0.002906889	Putative Fibrillarin
LITE_CONTIG_2423	Tc01g032860	AT1G48300	1.22	0.043498006	Putative uncharacterized protein
LITE_CONTIG_551	Tc05g031040	AT3G62550	1.22	0.007086693	Putative Universal stress protein A-like protein
KAS7YN21FM1_hypothetical	Tc06g000140	AT4G21130	1.21	0.002154754	Putative U3 small nucleolar RNA-interacting protein 2
LITE_CONTIG_1829	Tc09g015240	AT4G30440	1.21	0.020403325	UDP-glucuronate 4-epimerase 1
KAC6YN12FM1_WD40_like	Tc08g000320	AT3G49180	1.21	0.00573846	Predicted protein
KAA13YD05FM1_hypothetical	Tc05g005020	AT2G37500	1.20	0.00821019	Arginine biosynthesis bifunctional protein argJ
KCAE6YE19FM1_Transferase	Tc09g034830	AT5G07080	1.20	0.004928964	Putative 3'-N-debenzoyl-2'-deoxytaxol N-benzoyltransferase
LITE_CONTIG_6344	Tc02g003890	AT3G12500	1.19	0.034177888	Endochitinase 1

LITE_CONTIG_5864	Tc06g017020	AT4G05070	1.19	0.003683796	Predicted protein
KCL2YM16FM1_gi_30725320_gb_AAP37682_1_	Tc09g008760	AT5G19740	1.18	0.025859461	Putative Probable glutamate carboxypeptidase 2
KAP6YK02FM1_gi_22655334_gb_AAM98259_1_	Tc04g001960	AT3G01640	1.18	0.003175088	ATP binding protein, putative
328068416_m00329	Tc05g019710	AT3G03310	1.18	0.004499883	Phosphatidylcholine-sterol O-acyltransferase, putative
LITE_CONTIG_3454	Tc10g013330	AT5G06570	1.17	0.015221966	Catalytic, putative
LITE_CONTIG_6128	Tc00g012470	AT5G06360	1.16	0.001576497	Ribosome biogenesis protein NSA2 homolog
LITE_CONTIG_3122	Tc00g033030	AT5G48100	1.16	0.002906889	Putative Laccase-15
KAP6YM24FM1_hypothetical	Tc10g004090	AT5G56420	1.16	0.00517052	Putative F-box protein At2g39490
KBA1YK21RM1_	Tc09g030840	#N/A	1.16	0.010705279	Putative Predicted protein
KAS8YD08FM1_	Tc08g011870	#N/A	1.15	0.025993999	2-oxoisovalerate dehydrogenase subunit alpha, mitochondrial
LITE_CONTIG_916	Tc05g007980	AT3G10040	1.15	0.018694992	Putative uncharacterized protein
KAA13YD16FM1_gi_24417131_dbj_BAC22508_1_	Tc05g021030	AT3G03530	1.15	0.004103041	Putative Phospholipase C 3
LITE_CONTIG_1928	Tc09g031660	AT3G46570	1.15	0.017610971	Glucan endo-1,3-beta-glucosidase
KBA8YI17FM1_gi_22655372_gb_AAM98278_1_	Tc06g013580	AT4G18950	1.14	0.027435653	Protein kinase, putative
LITE_CONTIG_1747	Tc06g012110	AT1G30320	1.14	0.004428323	DNA binding protein, putative
LKBE5YA10FM1_	Tc09g006130	AT4G33150	1.14	0.006155641	Alpha-aminoadipic semialdehyde synthase
LITE_CONTIG_5639	Tc09g031840	AT2G28840	1.13	0.000762152	Putative Serine/threonine-protein phosphatase 6 regulatory ankyrin repeat subunit A
LITE_CONTIG_1074	Tc08g011150	AT1G21750	1.13	0.001846456	Protein disulfide-isomerase
KAP2YK01FM1_hypothetical	Tc03g025660	AT5G62360	1.13	0.006002845	Putative 21 kDa protein
LITE_CONTIG_2556	Tc05g003500	AT3G53990	1.12	0.001205893	Putative Universal stress protein A-like protein
LITE_CONTIG_1001	Tc05g001510	AT5G01710	1.12	0.004029511	Putative uncharacterized protein
KBA9YP24FM1_unnamed	Tc09g028890	AT5G22640	1.12	0.020254608	Putative uncharacterized protein
LITE_CONTIG_4738	Tc02g007060	AT4G34500	1.11	0.008779424	Probable serine/threonine-protein kinase At1g01540
KAC10YD05FM1_	Tc02g001030	#N/A	1.11	0.014034267	Putative uncharacterized protein
KBA4YC17FM1_hypothetical	Tc09g004520	AT2G25930	1.09	0.012953844	Putative Protein EARLY FLOWERING 3
KBB15YA11FM1_gi_27754219_gb_AAO22563_1_	Tc08g005530	AT3G21110	1.09	0.022900489	Phosphoribosylaminoimidazole-succinocarboxamide

					synthase, chloroplastic
LITE_CONTIG_6420	Tc01g009730	#N/A	1.09	0.032217633	Predicted protein
LITE_CONTIG_4185	Tc03g023410	AT5G46580	1.09	0.001173295	Pentatricopeptide repeat-containing protein At5g46580, chloroplastic
KAC14YC24FM1_gi_113534164_dbj_BAF06547_1_	Tc04g015880	AT5G01950	1.09	0.016056276	Probable LRR receptor-like serine/threonine-protein kinase At1g06840
KAV6YO01FM1_gi_16323464_gb_AAL15226_1_	Tc01g028010	AT1G03110	1.09	0.001173295	WD-repeat protein, putative
LITE_CONTIG_5783	Tc00g021240	AT5G53860	1.09	0.002906889	Putative uncharacterized protein
KAA14YD14FM1_unknown	Tc00g051160	AT3G13062	1.08	0.004396228	Predicted protein
KBF4YL16FM1_Vitis	Tc02g007860	#N/A	1.08	0.036882702	Putative uncharacterized protein
LITE_CONTIG_5421	Tc05g011630	AT5G06320	1.08	0.042969884	Hairpin-inducing protein
LITE_CONTIG_1517	Tc05g020320	AT3G13570	1.08	0.001665774	Serine/arginine rich splicing factor, putative
LKBE5YP16FM1_	Tc06g004420	#N/A	1.08	0.007739669	Probable WRKY transcription factor 40
LKBE9YD22FM1_gi_92891107_gb_ABE90649_1_	Tc10g003410	AT3G04020	1.08	0.001511507	Putative uncharacterized protein
KAQ11YL21FM1_gi_22136156_gb_AAM91156_1_	Tc09g030280	AT2G29630	1.08	0.015948037	Phosphomethylpyrimidine synthase
KAA12YI10FM1_	Tc09g034830	#N/A	1.07	0.006749612	Putative 3'-N-debenzoyl-2'-deoxytaxol N-benzoyltransferase
KCAE5YM16FM1_	Tc00g059000	#N/A	1.06	0.021937294	Hypothetical protein
KAC12YA21FM1_hypothetical	Tc01g034910	AT3G61620	1.06	0.004932371	Exosome complex exonuclease RRP41
KAA6YB22FM1_	Tc09g008500	#N/A	1.06	0.00128445	Zinc finger CCCH domain-containing protein 49
KCL2YN21FM1_Gryllus	#N/A	#N/A	1.06	0.005857069	#N/A
KBF6YE12FM1_General	Tc05g028940	AT1G54730	1.06	0.021937294	Sugar transporter ERD6-like 5
KAQ7YI03FM1_	Tc00g056020	#N/A	1.06	0.022623086	Putative WD repeat-containing protein 3
KAQ11YJ21FM1_Medicago	Tc03g018600	AT2G36530	1.05	0.00726844	Enolase
LITE_CONTIG_1198	Tc03g013550	AT4G11630	1.05	0.00284547	50S ribosomal protein L19, putative
LITE_CONTIG_625	Tc09g029790	AT1G07200	1.04	0.006554004	Putative uncharacterized protein
KAA8YI19FM1_	Tc00g026330	#N/A	1.04	0.003774691	Putative uncharacterized protein
KBB13YE15FM1_hypothetical	Tc03g026920	AT1G12770	1.04	0.040344917	DEAD-box ATP-dependent RNA helicase 47
KCAK1YG16FM1_auxin	Tc09g021860	AT5G20730	1.04	0.002257368	Auxin response factor 2
272168415_m00309	Tc01g004540	AT2G03500	1.04	0.00722239	Putative uncharacterized

					protein
LITE_CONTIG_1969	Tc07g008370	AT5G53880	1.04	0.019848659	Hypothetical protein
KCAE5YJ24FM1_hypothetical	Tc09g034830	AT3G47170	1.04	0.013251137	Putative 3'-N-debenzoyl-2'-deoxytaxol N-benzoyltransferase
LITE_CONTIG_3391	Tc06g000890	#N/A	1.03	0.013552938	UDP-arabinose 4-epimerase 1
2394_68416_m01579	Tc07g017330	AT3G12670	1.03	0.017263747	CTP synthase 1
LITE_CONTIG_5578	Tc02g023810	AT1G60420	1.03	0.00397464	Putative Nucleoredoxin
LITE_CONTIG_2938	Tc05g003590	AT5G02020	1.03	0.011912623	Putative uncharacterized protein
KCAK5YO23FM1_	Tc10g014970	#N/A	1.03	0.00821019	60S ribosomal protein L7-1
LKBE2YG20FM2_hypothetical	Tc02g003310	AT1G25280	1.03	0.001867512	Tubby-like F-box protein 8
1195_68414_m06479	Tc05g031090	AT1G56350	1.02	0.00194669	Putative Peptide chain release factor 2
CL498Contig1_hypothetical	Tc01g035600	AT4G23490	1.02	0.015221966	Predicted protein
LITE_CONTIG_1901	Tc09g030280	AT2G29630	1.02	0.00527387	Phosphomethylpyrimidine synthase
LKBE11YM01FM1_	Tc02g009470	#N/A	1.00	0.005563641	Putative Transcription factor MYB44
LITE_CONTIG_5395	Tc00g013490	AT1G54100	1.00	0.003185736	Aldehyde dehydrogenase family 7 member B4
KCAA5YH19FM1_gi_16323464_gb_AAL15226_1_	Tc01g028010	AT1G03110	1.00	0.001884843	WD-repeat protein, putative
LITE_CONTIG_6091	Tc07g017330	#N/A	1.00	0.04631044	CTP synthase 1
KAT7YJ20FM1_gi_7546693_emb_CAB87271_1_	Tc10g001090	AT5G07150	1.00	0.006414838	Putative Probable LRR receptor-like serine/threonine-protein kinase MRH1
KCAA4YB14FM1_	Tc00g086210	#N/A	1.00	0.006217635	Putative uncharacterized protein
LITE_CONTIG_4333	Tc08g011150	AT1G21750	1.00	0.025451735	Protein disulfide-isomerase
KAT9YD21FM1_hypothetical	Tc07g008920	AT1G29900	0.99	0.001766065	Carbamoyl-phosphate synthase large chain
KAQ10YP08FM1_gi_23193178_gb_AAN14412_1_	Tc09g006130	AT4G33150	0.99	0.007160609	Alpha-aminoadipic semialdehyde synthase
KCAA3YB23FM1_hypothetical	Tc04g011050	AT3G51550	0.99	0.039900131	Receptor-like protein kinase FERONIA
LITE_CONTIG_3531	Tc03g014010	AT4G13940	0.98	0.04631044	Adenosylhomocysteinase 1
KAA11YL05FM1_hypothetical	Tc08g007720	AT4G24230	0.98	0.034388543	Putative Acyl-CoA-binding domain-containing protein 3
KAC12YK07FM1_	Tc03g020090	#N/A	0.98	0.004321031	Putative Fibrillarin
LITE_CONTIG_6309	Tc03g013270	AT5G08180	0.98	0.008907029	H/ACA ribonucleoprotein complex subunit 2-like protein
LITE_CONTIG_3506	Tc02g002560	AT2G14260	0.98	0.003185736	Proline iminopeptidase
LITE_CONTIG_4077	#N/A	AT5G18790	0.97	0.036472855	#N/A
LITE_CONTIG_5346	Tc08g015000	AT1G22610	0.97	0.031536622	Synaptotagmin, putative
KBF8YE12FM1_XLG2	Tc02g002060	AT4G34390	0.96	0.042663747	GTP-binding protein alpha subunit, gna,

					putative
LITE_CONTIG_3111	Tc09g008740	#N/A	0.96	0.029606264	Putative uncharacterized protein
KAV7YI21FM1_SCARECROW_like	Tc09g031500	AT1G07520	0.96	0.013552938	Scarecrow-like protein 14
LITE_CONTIG_2922	Tc01g017030	AT2G32190	0.96	0.015221966	Hypothetical protein
LITE_CONTIG_6326	Tc05g009030	AT1G08360	0.96	0.004624992	60S ribosomal protein L10a-1
2110_68417_m04078	Tc02g016150	AT4G28510	0.96	0.006155641	Prohibitin-2
KAT2YP11FM1_gi_11994428_dbj_BAB02430_1_	Tc03g014600	AT3G12860	0.96	0.012429454	Nucleolar protein 56
LITE_CONTIG_595	Tc04g007870	AT5G39890	0.96	0.043407044	Putative 2-aminoethanethiol dioxygenase
KBF5YD21FM1_hypothetical	Tc10g005060	AT5G60390	0.95	0.016730927	Elongation factor 1-alpha
KAA10YM19FM1_initiation	Tc09g007590	AT3G13920	0.95	0.004599223	Eukaryotic initiation factor 4A-15
LITE_CONTIG_5185	Tc00g044370	AT5G22380	0.95	0.027965787	NAC domain-containing protein 90
KBB4YN05FM1_hypothetical	Tc02g007030	AT1G19400	0.95	0.007224288	Predicted protein
KCAE5YP11FM1_Peptidylprolyl	Tc08g007220	AT3G25230	0.94	0.026626013	70 kDa peptidyl-prolyl isomerase
LITE_CONTIG_5511	Tc04g011220	AT3G51550	0.94	0.006231508	Putative receptor-like protein kinase At5g39000
KCAA8YE18FM1_hypothetical	Tc02g013230	AT3G20000	0.94	0.005539549	Mitochondrial import receptor subunit TOM40 homolog 1
LITE_CONTIG_6834	Tc05g027100	AT5G28650	0.94	0.036464189	Probable WRKY transcription factor 74
LITE_CONTIG_5688	Tc08g006660	AT5G49650	0.94	0.007647574	Putative Xylulose kinase
1765_68417_m00151	Tc01g034940	AT4G01130	0.93	0.042663747	GDSL esterase/lipase At4g01130
KCAK4YF02FM1_hypothetical	Tc00g012140	AT3G23255	0.92	0.039673851	Putative uncharacterized protein
LITE_CONTIG_1832	Tc09g011890	AT5G20250	0.92	0.006178107	Probable galactinol--sucrose galactosyltransferase 6
KAQ9YH17FM1_hypothetical	Tc04g000320	AT3G28210	0.92	0.047917363	Zinc finger AN1 domain-containing stress-associated protein 12
KAC3YH10FM1_hypothetical	Tc00g051580	#N/A	0.91	0.04631044	Putative TMV resistance protein N
LITE_CONTIG_576	Tc00g024400	AT4G21700	0.91	0.042969884	Putative Predicted protein
LITE_CONTIG_981	Tc02g002560	AT2G14260	0.90	0.026016274	Proline iminopeptidase
LITE_CONTIG_5745	Tc06g006840	AT1G15500	0.89	0.024710958	Plastidic ATP/ADP-transporter
KCAF4YE23FM1_Rhabdodendron	Tc08g011990	#N/A	0.89	0.048675446	F-box/LRR-repeat protein 5
LITE_CONTIG_3364	Tc09g029650	AT3G47340	0.89	0.026551188	Asparagine synthetase [glutamine-hydrolyzing]
KAS2YI12FM1_bZIP	Tc02g006270	AT4G38900	0.89	0.027628576	DNA binding protein, putative
KCAE6YN04FM1_	Tc01g020050	#N/A	0.89	0.015126961	Predicted protein
LITE_CONTIG_809	Tc02g027190	AT1G23100	0.89	0.008588654	10 kDa chaperonin

KCAK3YP14FM1_gi_92893589_gb_ABE91815_1_	Tc03g000320	AT3G48120	0.89	0.036522972	Hypothetical protein
KAA1YL10_	Tc09g006470	#N/A	0.88	0.003606794	Putative RRP12-like protein
KCAF2YM14FM1_	Tc09g011650	#N/A	0.88	0.046631977	Putative Blue copper protein
KCAE4YD12FM1_SPla/RYanodine	Tc08g000110	#N/A	0.88	0.034527073	Putative uncharacterized protein
LITE_CONTIG_3868	Tc02g024400	AT1G60770	0.88	0.017989968	Pentatricopeptide repeat-containing protein At1g60770
LITE_CONTIG_5532	Tc03g027230	AT5G41190	0.88	0.046821036	RNA-binding protein nob1, putative
LITE_CONTIG_3355	Tc09g001360	AT4G31860	0.88	0.013279764	Probable protein phosphatase 2C 60
118968418_m05006	Tc03g027230	AT5G41190	0.88	0.03586944	RNA-binding protein nob1, putative
LKBE5YG18FM1_	Tc09g007190	#N/A	0.88	0.015752536	Predicted protein
LITE_CONTIG_518	Tc01g003380	#N/A	0.88	0.005631744	Putative F-box protein At5g03970
LITE_CONTIG_3706	Tc01g014030	AT4G17180	0.87	0.005631744	Glucan endo-1,3-beta-glucosidase 5
KBA1YE20FM1_hypothetical	Tc09g032150	AT5G01960	0.87	0.044315003	Putative Glycine-rich RNA-binding protein 2, mitochondrial
LITE_CONTIG_2275	Tc09g005730	AT5G21940	0.87	0.014632494	Putative uncharacterized protein
LITE_CONTIG_6570	Tc01g004520	AT5G66880	0.87	0.033171411	Serine/threonine-protein kinase SRK2I
KAT5YD05FM1_adenosylhomocysteinase	Tc03g014010	AT4G13940	0.87	0.010531728	Adenosylhomocysteinase 1
2172	Tc01g028210	AT3G63250	0.87	0.019251787	Hypothetical protein
KAP4YO16FM1_Unknown	Tc06g012290	AT1G65030	0.87	0.003117947	Putative p21-activated protein kinase-interacting protein 1
LITE_CONTIG_1289	Tc08g012890	AT1G43170	0.86	0.021096476	60S ribosomal protein L3
KCAK5YM17FM1_hypothetical	Tc04g029310	AT2G19640	0.86	0.02559761	Histone-lysine N-methyltransferase ASHR2
LITE_CONTIG_161	Tc09g028860	#N/A	0.86	0.014009834	Hypothetical protein
KCAE6YE13FM1_	Tc03g013880	#N/A	0.86	0.02660485	Putative Predicted protein
KBB14YE14FM1_Solute	Tc01g036920	AT1G12600	0.85	0.003677679	Putative Adenosine 3'-phospho 5'-phosphosulfate transporter 2
KAS9YF02FM1_hypothetical	Tc02g004600	AT4G38500	0.85	0.013369257	Putative uncharacterized protein
KCAA4YL20FM1_protein	Tc10g014000	AT3G12270	0.85	0.011687593	Probable protein arginine N-methyltransferase 3
2501_68414_m00636	Tc04g019520	AT1G06070	0.85	0.005539549	Probable transcription factor PosF21
LITE_CONTIG_5615	Tc00g051160	AT1G55960	0.85	0.014632494	Predicted protein
LITE_CONTIG_5534	Tc09g031840	AT2G28840	0.85	0.007739669	Putative Serine/threonine-protein phosphatase 6 regulatory ankyrin repeat subunit A

KBA10YB09FM1_gi_20259906_gb_AAM13300_1_	Tc09g034790	AT1G16870	0.85	0.003677679	Putative uncharacterized protein
LITE_CONTIG_1070	Tc06g012240	AT2G20490	0.84	0.041764816	H/ACA ribonucleoprotein complex subunit 3-like protein
KBF3YG20FM1_gi_740202_prf__2004427A	Tc00g057870	AT4G09670	0.84	0.005722463	Uncharacterized oxidoreductase At4g09670
KCAK4YM15FM1_hypothetical	Tc04g010150	AT3G29310	0.84	0.030306466	Predicted protein
LITE_CONTIG_4430	Tc03g008330	AT5G61440	0.84	0.035302716	Thioredoxin-like 3, chloroplastic
KAS4YK07FM1_hypothetical	Tc04g014670	AT3G02570	0.84	0.033358875	Putative Mannose-6-phosphate isomerase
KAQ10YK01FM1_gi_20197006_gb_AAC23761_2_	Tc02g002060	AT2G23460	0.84	0.033786456	GTP-binding protein alpha subunit, gna, putative
CL355Contig1_gi_114050659_gb_ABI49479_1_	Tc05g005260	AT2G37400	0.84	0.033171411	Putative uncharacterized protein
LITE_CONTIG_6153	Tc06g021200	#N/A	0.84	0.009335489	Putative Pentatricopeptide repeat-containing protein At1g09410
LITE_CONTIG_4766	Tc04g004800	AT5G14030	0.84	0.003677679	Translocon-associated protein, beta subunit, putative
KAP7YA15FM1_hypothetical	Tc02g000370	AT1G05300	0.83	0.014632494	Zinc transporter 1
LITE_CONTIG_1161	Tc09g025350	AT1G14980	0.83	0.041724361	10 kDa chaperonin
KCL1YD15FM1_tuber_specific	Tc02g009470	AT4G37260	0.83	0.034295921	Putative Transcription factor MYB44
KBB10YB19FM1_hypothetical	Tc09g012150	#N/A	0.83	0.019612424	Predicted protein
LITE_CONTIG_2329	Tc02g013990	#N/A	0.83	0.011051363	Hypothetical protein
KAP6YC07FM1_gi_21554564_gb_AAM63615_1_	Tc05g010140	AT2G36310	0.83	0.022235224	Putative Uncharacterized protein C1683.06c
KBB10YL09FM1_Medicago	Tc00g091950	AT4G17900	0.83	0.00821019	Putative Uncharacterized protein At3g50808
LITE_CONTIG_1354	Tc04g001750	AT5G40510	0.82	0.008775355	Predicted protein
LITE_CONTIG_5093	Tc00g054510	AT3G52580	0.82	0.017107771	40S ribosomal protein S14
LITE_CONTIG_506	Tc01g035070	#N/A	0.82	0.00830391	rRNA-processing protein FCF1 homolog
CL456Contig1_hypothetical	Tc04g004840	AT5G13200	0.82	0.033171411	GEM-like protein 5
LITE_CONTIG_4613	Tc04g023350	AT3G05990	0.82	0.015204087	Putative Probable LRR receptor-like serine/threonine-protein kinase At1g67720
KAA4YK03FM1_gi_38017093_gb_AAR07942_1_	Tc00g015920	AT3G10690	0.82	0.016243962	DNA gyrase subunit A, chloroplastic/mitochondrial
KBA9YB22FM1_Putative	Tc06g000890	AT1G30620	0.82	0.00508481	UDP-arabinose 4-epimerase 1
LITE_CONTIG_837	Tc08g001140	AT1G47710	0.81	0.003261717	Serpin-ZX
KAP24YA06FM1_gi_	Tc00g068210	AT5G14800	0.81	0.004428323	Pyrroline-5-carboxylate

18724_emb_CAA3440 1_1_					reductase
LITE_CONTIG_6640	Tc02g004940	AT3G12920	0.81	0.012429454	Putative uncharacterized protein
LITE_CONTIG_2960	Tc06g009950	AT1G72830	0.81	0.020254608	Nuclear transcription factor Y subunit A-3
KBA10YD22FM1_sucrose	Tc08g015910	AT1G22710	0.81	0.023345751	Sucrose transport protein SUC2
LITE_CONTIG_3125	Tc07g000090	AT5G04800	0.81	0.012723784	40S ribosomal protein S17-4
KCAE8YO22FM1_gi_110742332_dbj_BAE99090_1_	Tc01g028890	AT4G02930	0.80	0.047417893	Elongation factor Tu, mitochondrial
LITE_CONTIG_5084	Tc00g068210	AT5G14800	0.80	0.007160609	Pyrroline-5-carboxylate reductase
LITE_CONTIG_6482	Tc09g030630	AT2G29530	0.80	0.025303384	Mitochondrial import inner membrane translocase subunit Tim10
LITE_CONTIG_1331	Tc05g015540	#N/A	0.80	0.028008878	Putative DNA-directed RNA polymerases I, II, and III subunit RPABC1
LITE_CONTIG_5836	Tc01g000250	AT3G55620	0.80	0.00951841	Eukaryotic translation initiation factor 6
KAS3YM14FM1_hypothetical	Tc06g010500	AT1G72440	0.80	0.033171411	Putative CCAAT/enhancer-binding protein zeta
LITE_CONTIG_1767	Tc07g017060	AT5G61170	0.80	0.035902113	40S ribosomal protein S19-3
KAC13YD24FM1_putative	Tc00g054580	AT5G22330	0.80	0.006217635	RuvB-like 1
KAC10YD24FM1_hypothetical	Tc04g023340	AT5G19330	0.80	0.034388543	Putative Vacuolar protein 8
LKBE1YN20RM1_	Tc03g029640	#N/A	0.79	0.024605103	Predicted protein
KBB10YL20FM1_gi_2832898_emb_CAA04386_1_	Tc04g028360	AT3G06860	0.79	0.032238868	Glyoxysomal fatty acid beta-oxidation multifunctional protein MFP-a
KAQ4YI19FM1_gi_125575086_gb_EAZ16370_1_	Tc04g026210	AT1G04480	0.79	0.033405324	60S ribosomal protein L23
KCAE7YO08FM1_gi_117168113_gb_ABK32139_1_	Tc04g026300	AT4G21865	0.79	0.033643917	Putative uncharacterized protein
LITE_CONTIG_5470	Tc05g028690	#N/A	0.79	0.030306466	Putative Disease resistance protein At4g27190
KAT9YH02FM1_hypothetical	Tc07g001020	AT1G55850	0.79	0.021096476	Cellulose synthase-like protein E1
KAA10YO13FM1_gi_125570288_gb_EAZ11803_1_	Tc04g028360	AT3G06860	0.78	0.041724361	Glyoxysomal fatty acid beta-oxidation multifunctional protein MFP-a
LITE_CONTIG_2901	Tc05g028310	AT5G27120	0.78	0.019468801	Probable nucleolar protein 5-1
KAV13YP15FM1_gi_51971449_dbj_BAD44389_1_	Tc08g003010	AT1G75560	0.78	0.022584614	Putative DNA-binding protein HEXBP
KBA6YO09FM1_	Tc02g007960	#N/A	0.78	0.00517052	Putative uncharacterized protein

LITE_CONTIG_2443	Tc02g002540	AT4G37760	0.78	0.038474643	Squalene monooxygenase
LITE_CONTIG_125	Tc06g009950	#N/A	0.77	0.043616523	Nuclear transcription factor Y subunit A-3
KAT5YJ08FM1_gi_147816494_emb_CAN77349_1_	Tc01g001220	AT4G35100	0.77	0.014746545	Aquaporin PIP2-7
KAV3YF03FM1_EDS1	Tc03g000280	AT3G48080	0.77	0.029178215	Lipase, putative
LITE_CONTIG_5686	Tc04g018470	AT1G10840	0.77	0.021592643	Eukaryotic translation initiation factor 3 subunit H
KAA9YA18FM1_	#N/A	#N/A	0.77	0.039673851	#N/A
LITE_CONTIG_2506	Tc05g027610	AT1G60680	0.77	0.03586944	Auxin-induced protein PCNT115
LITE_CONTIG_2753	Tc02g034060	AT1G07040	0.77	0.019759174	Putative uncharacterized protein
KAA2YK09FM1_hypothetical	Tc00g046250	AT4G23990	0.76	0.046203644	Putative Cellulose synthase-like protein E6
KAA12YA21FM1_F3F19_5	Tc02g025170	AT1G13030	0.76	0.021538955	Putative uncharacterized protein
LITE_CONTIG_527	Tc06g010970	AT3G53690	0.76	0.049265198	Predicted protein
LITE_CONTIG_2232	Tc01g030440	AT2G47710	0.76	0.023926457	Putative Universal stress protein A-like protein
LITE_CONTIG_1007	Tc06g013680	AT1G07650	0.76	0.011638067	Putative Probable LRR receptor-like serine/threonine-protein kinase At1g07650
LITE_CONTIG_6274	Tc04g017610	AT1G05710	0.76	0.003606794	Transcription factor bHLH113
KAA12YJ17FM1_	Tc06g007970	#N/A	0.76	0.032238868	Putative Zinc phosphodiesterase ELAC protein 2
LITE_CONTIG_1524	Tc09g034680	AT5G58430	0.76	0.015948037	Putative uncharacterized protein
LITE_CONTIG_1343	Tc06g012620	AT1G30070	0.76	0.008444627	Putative Calcyclin-binding protein
KAA14YH02FM1_alcohol	Tc00g022670	AT1G32780	0.76	0.015778403	Putative Alcohol dehydrogenase-like 5
LITE_CONTIG_1114	Tc05g027990	AT3G04920	0.76	0.035136656	40S ribosomal protein S24-2
KAS9YK01FM1_protein	Tc09g001360	AT4G31860	0.76	0.009287559	Probable protein phosphatase 2C 60
LITE_CONTIG_801	Tc03g017010	AT4G16720	0.76	0.028756367	60S ribosomal protein L15
LITE_CONTIG_6267	Tc01g005580	AT2G23320	0.75	0.024710958	Putative Probable WRKY transcription factor 7
LITE_CONTIG_2945	Tc03g011540	AT5G61210	0.75	0.017128993	SNAP25 homologous protein SNAP33
LITE_CONTIG_4208	Tc09g033130	AT2G27080	0.75	0.040923658	Predicted protein
LITE_CONTIG_569	Tc02g031310	AT1G26270	0.75	0.033171411	Inositol or phosphatidylinositol kinase, putative
KAP8YM11FM1_gi_113537888_dbj_BAF10271_1_	Tc09g016020	AT5G57740	0.75	0.043366754	Putative Serine/threonine-protein phosphatase 6 regulatory ankyrin repeat subunit B
KAS2YG01FM1_hypothetical	Tc09g008660	AT4G29310	0.74	0.032716806	Putative uncharacterized protein

CL209Contig1_	Tc04g020160	#N/A	0.74	0.045872391	Putative uncharacterized protein
LITE_CONTIG_1502	Tc08g015260	AT4G09160	0.74	0.013251137	Patellin-3, putative
KBA8YF20FM1_gi_110735833_dbj_BAE99893_1_	Tc05g008810	AT2G36290	0.74	0.040344917	Catalytic, putative
LITE_CONTIG_5336	Tc05g031940	AT5G26731	0.74	0.021614094	Hypothetical protein
LITE_CONTIG_6146	Tc02g007070	AT4G34490	0.74	0.023004969	Putative Adenylyl cyclase-associated protein
KAT1YL12RM1_hypothetical	Tc07g014090	AT1G56140	0.74	0.033042373	Probable LRR receptor-like serine/threonine-protein kinase At1g56130
KAC6YI16FM1_hypothetical	Tc06g017480	AT1G55890	0.74	0.043407044	Putative Pentatricopeptide repeat-containing protein At3g13150
LITE_CONTIG_2584	Tc09g030410	AT3G46980	0.73	0.019139778	Probable anion transporter 4, chloroplastic
KCAF4YK11FM1_stpk1	Tc04g001740	AT3G27580	0.73	0.019604988	Protein kinase PVPK-1
LITE_CONTIG_6770	Tc02g013990	#N/A	0.73	0.034731082	Hypothetical protein
LITE_CONTIG_4609	Tc09g001540	AT5G25110	0.73	0.017347881	CBL-interacting serine/threonine-protein kinase 25
LITE_CONTIG_1720	Tc03g011650	AT5G61170	0.73	0.014853248	40S ribosomal protein S19-3
KAQ5YE20FM1_hypothetical	Tc08g005810	AT5G49700	0.73	0.032784308	Predicted protein
LITE_CONTIG_6103	Tc01g020660	AT5G39740	0.73	0.036841753	60S ribosomal protein L5
LITE_CONTIG_2744	Tc05g005930	AT5G02960	0.73	0.046711797	40S ribosomal protein S23
1486_68418_m01713	Tc04g001160	AT5G14610	0.73	0.033171411	DEAD-box ATP-dependent RNA helicase 46
LITE_CONTIG_3107	Tc01g019470	AT3G07568	0.73	0.01448652	Putative uncharacterized protein
KCAF5YJ12FM1_F22G5_8	Tc09g031520	AT1G07540	0.72	0.032238868	Putative Telomere-binding protein 1
LITE_CONTIG_5367	Tc02g007050	#N/A	0.72	0.025859461	Predicted protein (Fragment)
LITE_CONTIG_6139	Tc03g021310	AT4G27740	0.72	0.014632494	Protein yippee-like At4g27740
CL244Contig1_	Tc01g010870	#N/A	0.72	0.010576701	Predicted protein
KAC5YK08FM1_hypothetical	Tc06g017000	AT4G21450	0.72	0.004928964	Predicted protein
3278_68417_m01804	Tc01g019670	AT4G11120	0.72	0.006036646	Putative Elongation factor Ts
LITE_CONTIG_4421	Tc01g039890	AT2G44670	0.72	0.021945146	Putative uncharacterized protein
LITE_CONTIG_6754	Tc02g020210	AT1G67360	0.72	0.013767738	REF/SRPP-like protein At1g67360
LITE_CONTIG_3216	Tc01g006470	AT4G37640	0.72	0.048739772	Calcium-transporting ATPase 2, plasma membrane-type
LITE_CONTIG_543	Tc01g035310	AT3G61540	0.71	0.034388543	Putative Proline iminopeptidase
LITE_CONTIG_3153	Tc00g005620	AT3G44190	0.71	0.017128993	Putative Apoptosis-

					inducing factor homolog A
LITE_CONTIG_5047	Tc02g008740	AT4G34412	0.71	0.017734045	Putative TP53RK-binding protein
KBA10YH21FM1_	Tc09g011720	#N/A	0.71	0.025809222	Putative clathrin assembly protein At2g25430
KCAK5YI08FM1_	Tc05g003980	#N/A	0.71	0.019139778	Putative Probable E3 ubiquitin-protein ligase HERC1
LITE_CONTIG_4485	Tc01g016490	AT3G49010	0.71	0.031913266	60S ribosomal protein L13-1
KAP6YL19FM1_gi_110737014_dbj_BAF00462_1_	Tc09g029800	#N/A	0.70	0.011638067	Putative 30S ribosomal protein S18
KAC8YF04FM1_gi_125584123_gb_EAZ25054_1_	Tc01g022340	AT3G07090	0.70	0.032784308	Putative PPPDE peptidase domain-containing protein 2
LITE_CONTIG_1741	Tc04g001610	AT5G14520	0.70	0.037127704	Putative Pescadillo homolog
LITE_CONTIG_2289	Tc00g085480	AT2G40830	0.70	0.019604988	Predicted protein
KBA8YL14FM1_	Tc08g001710	#N/A	0.70	0.036841753	Putative uncharacterized protein
LITE_CONTIG_4832	Tc06g009950	#N/A	0.70	0.021889293	Nuclear transcription factor Y subunit A-3
LITE_CONTIG_2069	Tc09g032190	AT5G59850	0.70	0.032258866	40S ribosomal protein S15a-1
LITE_CONTIG_1276	Tc05g017360	AT2G41620	0.70	0.010133718	Uncharacterized protein At2g41620
LITE_CONTIG_6211	Tc04g004060	AT3G62870	0.70	0.033786456	60S ribosomal protein L7a
LITE_CONTIG_5196	Tc03g029260	AT5G42050	0.69	0.006155641	Predicted protein
LITE_CONTIG_5358	Tc01g016120	AT3G61110	0.69	0.024826266	40S ribosomal protein S27-2
347968414_m03244	Tc02g032660	AT1G26640	0.69	0.034901374	Putative Uncharacterized protein MJ0044
LKBE7YP17FM1_gi_7268982_emb_CAB80715_1_	Tc05g019320	AT4G02220	0.69	0.032238868	Putative Programmed cell death protein 2
KAC4YJ15FM1_gi_113537348_dbj_BAF09731_1_	Tc03g029950	#N/A	0.69	0.022900489	Predicted protein
KAC4YI03FM1_gi_20259173_gb_AAM14302_1_	Tc04g029320	AT2G39990	0.69	0.011638067	Eukaryotic translation initiation factor 3 subunit F
LITE_CONTIG_5655	Tc00g033720	AT1G53280	0.69	0.012837511	Putative Protein thiJ
LITE_CONTIG_2492	Tc09g033180	AT5G21090	0.69	0.022900489	BRASSINOSTEROID INSENSITIVE 1-associated receptor kinase 1
LITE_CONTIG_4209	Tc03g028070	AT4G11240	0.69	0.009382801	Serine/threonine-protein phosphatase PP1
CL172Contig1_gi_21389677_gb_AAM48037_1_	Tc02g010310	AT3G49870	0.69	0.034901374	ADP-ribosylation factor-like protein 8A
LITE_CONTIG_4991	Tc06g009090	AT1G54270	0.68	0.026145685	Eukaryotic initiation factor 4A-14
LITE_CONTIG_3544	Tc00g009520	AT1G75080	0.68	0.010814412	BES1/BZR1 homolog protein 2
LITE_CONTIG_2050	Tc02g017210	AT1G67430	0.68	0.042230336	60S ribosomal protein

					L17-2
LITE_CONTIG_781	Tc01g029100	AT3G02560	0.68	0.047542921	40S ribosomal protein S7
LITE_CONTIG_5328	Tc02g016440	AT4G28390	0.68	0.007895851	ADP,ATP carrier protein, mitochondrial
LITE_CONTIG_3147	Tc01g033530	AT1G01050	0.68	0.048462398	Soluble inorganic pyrophosphatase
LITE_CONTIG_596	Tc06g015060	AT1G28540	0.68	0.041724361	Hypothetical protein
CL336Contig1_hypothetical	Tc05g025560	AT5G63930	0.67	0.040657308	Probable leucine-rich repeat receptor-like protein kinase At2g33170
KCL4YK24FM1_	Tc01g027040	#N/A	0.67	0.018403318	Ring finger protein, putative
KCAA3YA01FM1_gi_6984142_gb_AAF34771_1_AF227626_1	Tc01g039910	AT5G23740	0.67	0.027965787	40S ribosomal protein S11
LITE_CONTIG_2168	Tc02g034130	AT3G25890	0.67	0.034212479	Putative Ethylene-responsive transcription factor ERF118
LITE_CONTIG_4776	Tc03g029450	#N/A	0.67	0.027499766	ABC transporter F family member 3
LITE_CONTIG_234	Tc10g001880	AT3G09200	0.67	0.021983647	60S acidic ribosomal protein P0-1
LKBE7YH17FM1_	Tc01g011440	#N/A	0.67	0.029580421	Putative Elongation factor Tu GTP-binding domain-containing protein 1
KCAE3YH14FM1_Ttg1_like	Tc03g021830	AT5G24520	0.67	0.023288197	Protein TRANSPARENT TESTA GLABRA 1
KCAA8YG18FM1_CTV_2	Tc06g003470	AT1G80490	0.67	0.042908029	CTV.2
KBB13YE17FM1_RNA	Tc05g015540	AT3G57080	0.67	0.021715719	Putative DNA-directed RNA polymerases I, II, and III subunit RPABC1
KBB15YH07FM1_gi_25090122_gb_AAN72234_1_	Tc09g001600	AT2G25140	0.67	0.048925005	Chaperone protein clpB 2
LITE_CONTIG_5357	Tc09g032400	AT5G02610	0.67	0.035921087	60S ribosomal protein L35
287968417_m02487	Tc01g026730	AT4G16430	0.67	0.049579415	Transcription factor bHLH3
KCAK5YE11FM1_gi_23296881_gb_AAN13194_1_	Tc01g022340	AT3G07090	0.66	0.026626013	Putative PPPDE peptidase domain-containing protein 2
KBA8YL17FM1_	Tc06g019820	#N/A	0.66	0.036497444	50S ribosomal protein L4
KCAE7YI22FM1_gi_70905083_gb_AAZ14067_1_	Tc08g001500	AT1G65430	0.66	0.019759174	Probable E3 ubiquitin-protein ligase ARI8
KCAK4YJ11FM1_gi_7267815_emb_CAB81217_1_	Tc02g007260	AT4G11160	0.66	0.040883336	Putative Translation initiation factor IF-2
KBB12YI07FM1_gi_22136308_gb_AAM91232_1_	Tc01g018300	AT2G04540	0.66	0.042760685	3-oxoacyl-[acyl-carrier-protein] synthase, mitochondrial
KAS10YP17FM1_phosphoribosylformylglycinamidine	Tc05g006050	AT1G74260	0.66	0.035902094	Probable phosphoribosylformylglycinamidine synthase,

					chloroplastic
KAT1YF18_dormancy /auxin	Tc06g010440	AT1G54070	0.66	0.013225358	Hypothetical protein
LITE_CONTIG_1376	Tc06g012800	AT2G34480	0.65	0.029608426	60S ribosomal protein L18a
LITE_CONTIG_6333	Tc01g022430	AT5G48760	0.65	0.031741991	60S ribosomal protein L13a-4
LITE_CONTIG_2964	Tc01g033710	AT5G24510	0.65	0.039900131	60S acidic ribosomal protein P1-1
LITE_CONTIG_718	Tc04g022440	AT5G48760	0.65	0.024605103	60S ribosomal protein L13a-4
LITE_CONTIG_1347	Tc03g015330	AT3G62870	0.64	0.029422501	60S ribosomal protein L7a
KAS2YL20FM1_gi_125572842_gb_EAZ14357_1_	Tc01g013340	AT1G33420	0.64	0.036095915	PHD finger protein At1g33420
LITE_CONTIG_3351	Tc06g015310	AT2G20560	0.64	0.04503792	Putative DnaJ homolog subfamily B member 4
KAQ2YC19FM1_beta_glucan_binding	Tc00g009650	AT5G15870	0.64	0.040305303	Putative Endo-1,3(4)-beta-glucanase 1
LITE_CONTIG_3089	Tc01g039980	AT2G44610	0.64	0.012271883	Ras-related protein RABH1B
KCAA3YH12FM1_hypothetical	Tc09g034260	AT5G58575	0.64	0.032238868	Putative uncharacterized protein
2560_68414_m00514	Tc02g035030	AT1G05120	0.64	0.033171411	DNA repair helicase rad5,16, putative
LITE_CONTIG_5280	Tc03g019310	AT1G12580	0.64	0.047917363	Predicted protein
LITE_CONTIG_849	Tc05g031350	AT5G26790	0.64	0.023361789	Putative uncharacterized protein
KBB13YD11FM1_gi_10177347_dbj_BAB10690_1_	Tc01g021030	AT5G48570	0.64	0.015778403	70 kDa peptidyl-prolyl isomerase
KAV13YD01FM1_gi_24796986_gb_AAN64505_1_	Tc00g036580	AT3G28480	0.63	0.019623013	Prolyl 4-hydroxylase alpha subunit, putative
KAQ9YK20FM1_	Tc00g005690	#N/A	0.63	0.047917363	Putative Bystin
KCAK1YO13FM1_	Tc05g019600	#N/A	0.63	0.027086748	Probable nucleolar GTP-binding protein 1
LITE_CONTIG_85	Tc01g028240	#N/A	0.63	0.019964965	Putative uncharacterized protein
LITE_CONTIG_5603	Tc01g036970	AT2G45695	0.63	0.020466779	Ubiquitin-related modifier 1 homolog 1
LITE_CONTIG_1606	Tc08g001270	AT1G47640	0.63	0.021124088	Predicted protein
LITE_CONTIG_6120	Tc02g002030	AT2G15910	0.63	0.034388543	Diphthamide biosynthesis protein 3
LKBE9YB22FM1_	Tc01g010180	#N/A	0.63	0.029422501	Putative Cytochrome P450 86B1
LITE_CONTIG_3172	Tc09g005770	AT2G26300	0.62	0.018375213	Guanine nucleotide-binding protein alpha-1 subunit
LITE_CONTIG_6675	Tc04g028310	AT1G04170	0.62	0.01643745	Eukaryotic translation initiation factor 2 subunit 3
KBB3YP17FM1_hypothetical	Tc07g007460	AT4G03230	0.62	0.034388543	Putative serine/threonine-protein kinase receptor
LITE_CONTIG_2560	Tc10g002180	AT2G39920	0.62	0.027628576	Putative Uncharacterized protein At2g39920
LITE_CONTIG_5140	Tc01g016740	AT2G34480	0.62	0.024863315	60S ribosomal protein L18a

CL115Contig1_hypothetical	Tc05g003160	AT2G38000	0.62	0.046711797	Putative Uncharacterized protein C3orf32
LITE_CONTIG_1629	Tc09g033360	AT3G54960	0.61	0.042366168	Putative Protein disulfide-isomerase
3531_68415_m02556	Tc01g005800	AT2G21480	0.61	0.046917667	Probable receptor-like protein kinase At2g23200
KAS8YK24FM1_gi_27311895_gb_AAO00913_1_	Tc05g022280	AT5G16880	0.61	0.035817079	Protein transporter, putative
KCAK1YB22FM1_hypothetical	Tc01g019290	AT5G48240	0.61	0.021600835	Putative uncharacterized protein
LITE_CONTIG_6588	Tc03g000030	AT1G52600	0.61	0.015221966	Signal peptidase complex catalytic subunit SEC11C
KAV15YD13FM1_hypothetical	Tc02g009070	AT4G34100	0.60	0.038474643	Putative E3 ubiquitin-protein ligase MARCH6
LITE_CONTIG_3060	Tc03g010290	AT1G18800	0.60	0.032182525	Putative Protein SET
LITE_CONTIG_3697	Tc00g029090	#N/A	0.60	0.042842201	Hypothetical protein
LITE_CONTIG_6461	Tc03g020370	AT3G53740	0.60	0.047151633	60S ribosomal protein L36-2
LITE_CONTIG_5373	Tc05g022350	AT3G03050	0.60	0.045699391	Cellulose synthase-like protein D3
KAP13YI10FM1_	Tc09g025960	#N/A	0.60	0.040883336	Putative Heterogeneous nuclear ribonucleoprotein F
LITE_CONTIG_1118	Tc03g021500	AT3G52590	0.60	0.033275882	Ubiquitin
LITE_CONTIG_4513	Tc00g021060	AT3G16080	0.59	0.034212479	60S ribosomal protein L37-3
LITE_CONTIG_1011	Tc01g040250	AT5G13780	0.59	0.032268854	N-alpha-acetyltransferase 11, NatA catalytic subunit
LITE_CONTIG_2523	Tc01g016720	AT1G53580	0.59	0.042230336	Hydroxyacylglutathione hydrolase 3, mitochondrial
1696_68418_m06717	Tc10g002860	AT5G54000	0.59	0.028329439	Putative Protein SRG1
LITE_CONTIG_4425	Tc06g016580	AT1G61250	0.58	0.021339339	Secretory carrier-associated membrane protein 3
LITE_CONTIG_6802	Tc00g008140	AT1G79340	0.58	0.043616523	Metacaspase-5
KAA10YD19FM1_hypothetical	Tc07g014260	AT1G16670	0.57	0.036882702	Probable LRR receptor-like serine/threonine-protein kinase At1g56130
LITE_CONTIG_6208	Tc01g028600	AT4G02840	0.57	0.047417893	Small nuclear ribonucleoprotein sm d1, putative
241468415_m05905	Tc01g031870	AT2G47300	0.57	0.042024332	Putative uncharacterized protein
KCAK5YO08FM1_hypothetical	Tc06g010420	AT1G72410	0.57	0.04588655	Predicted protein
LITE_CONTIG_1130	Tc06g011720	AT1G04750	0.57	0.041681587	Putative vesicle-associated membrane protein 726
LITE_CONTIG_4274	Tc03g012560	AT3G08900	0.56	0.047729009	Alpha-1,4-glucan-protein synthase [UDP-forming] 2
223468416_m06520	Tc05g020520	AT3G58500	0.56	0.034212479	Serine/threonine-protein phosphatase PP2A-3 catalytic subunit

LITE_CONTIG_2145	Tc10g008630	AT3G20970	0.56	0.032238868	NifU-like protein 4, mitochondrial
327468418_m04778	Tc01g012390	AT5G39450	0.55	0.03224542	F-box protein At5g39450
KAC11YC05FM1_	Tc05g005760	#N/A	0.55	0.025457422	Pentatricopeptide repeat-containing protein At5g02830, chloroplastic
KCL2YE02FM1_calcium_dependent	Tc00g035530	AT5G04870	0.55	0.042759845	Calcium-dependent protein kinase 1
KAQ9YD10FM1_	#N/A	#N/A	0.55	0.04588655	#N/A
KAA13YB07FM1_hypothetical	Tc04g001360	AT5G14590	0.55	0.032238868	Isocitrate dehydrogenase [NADP], chloroplastic (Fragment)
KAT9YI12FM1_Zinc	Tc09g010720	AT1G11020	0.55	0.042579842	Predicted protein
LITE_CONTIG_4426	Tc00g025150	AT5G17165	0.54	0.048611343	Putative uncharacterized protein
LITE_CONTIG_4384	Tc01g015490	AT5G19630	0.54	0.032238868	Catalytic, putative
LITE_CONTIG_4440	Tc09g028580	AT2G27720	0.54	0.047729009	60S acidic ribosomal protein P2B
KAQ11YD04FM1_unknown	Tc08g001840	AT2G30942	0.54	0.042663747	Predicted protein
LITE_CONTIG_5933	Tc09g028570	AT3G44590	0.52	0.043407044	60S acidic ribosomal protein P2B
LITE_CONTIG_1153	Tc09g007200	AT5G35530	0.52	0.032238868	40S ribosomal protein S3-3
KAP14YH20FM1_Takifugu	Tc00g080320	#N/A	0.52	0.036522972	Nucleolar essential protein, putative
LITE_CONTIG_6199	Tc07g011170	AT1G53320	0.51	0.046165983	Tubby-like F-box protein 7
LITE_CONTIG_2056	Tc07g001020	AT3G07680	0.51	0.048928387	Cellulose synthase-like protein E1
LITE_CONTIG_5599	Tc02g013270	AT5G08520	0.51	0.036522972	Predicted protein
LITE_CONTIG_4508	Tc06g020180	AT4G22330	0.51	0.04588655	Putative Alkaline ceramidase 3
LITE_CONTIG_691	Tc04g012970	AT3G02200	0.50	0.030862631	Putative Eukaryotic translation initiation factor 3 subunit M
KAQ11YK21FM1_WD_40	Tc08g015730	AT1G73720	0.45	0.048739772	WD40 repeat-containing protein SMU1

Supplementary Table 7. ***Theobroma cacao*** **genes down-regulated in leaves inoculated with endophyte** ***C. tropicale*** **(E+) at 3 days post inoculation compared to control E- leaves (2nd microarray experiment).**

Oligo ID (unigene)	Tc Identifier	At Identifier	logFC	adj.P.Val (BH)	Annotation
381168417_m03887	Tc07g004100	AT4G27030	-6.63	6.69E-07	Predicted protein
LITE_CONTIG_199	Tc10g016510	#N/A	-4.53	1.93E-05	Predicted protein
LITE_CONTIG_339	Tc07g004100	#N/A	-4.45	1.80E-06	Predicted protein
KAT3YC20FM1_hypothetical	Tc09g009090	AT2G25770	-4.02	0.001154148	Putative Lachrymatory-factor synthase
LITE_CONTIG_1135	Tc09g016580	AT4G25780	-3.76	7.42E-06	STS14 protein, putative
KAA7YM07FM1_gi_98962119_gb_ABF59389_1_	Tc00g080340	AT4G15248	-3.66	9.22E-06	Putative Predicted protein
LITE_CONTIG_51	Tc03g000730	#N/A	-3.61	0.00128445	Hypothetical protein
LKBE13YG18FM1_gi_16323224_gb_AAL15346_1_	Tc03g018830	AT5G51720	-3.34	0.018599241	Predicted protein
LITE_CONTIG_6567	Tc04g024360	AT4G14690	-3.21	3.61E-05	Early light-induced protein, chloroplastic
LITE_CONTIG_1575	Tc02g024030	AT1G10657	-3.00	0.019835102	Predicted protein
LITE_CONTIG_96	Tc03g030500	#N/A	-2.91	0.000176907	Putative uncharacterized protein
LITE_CONTIG_1593	Tc02g033450	AT1G69640	-2.91	0.000762152	Putative Uncharacterized hydroxylase C887.15c
LITE_CONTIG_1786	Tc06g019310	AT4G04610	-2.86	0.013805483	5'-adenylylsulfate reductase 3, chloroplastic
LITE_CONTIG_1482	Tc04g002490	AT2G40435	-2.81	0.000898366	Putative Transcription factor bHLH61
1194_68417_m05187	Tc01g002990	AT4G36530	-2.79	0.000241648	Putative Uncharacterized hydrolase yugF
279568416_m01867	Tc06g013480	AT3G14770	-2.72	0.000166852	Putative Protein RUPTURED POLLEN GRAIN 1
131668416_m05111	Tc09g030230	AT3G47070	-2.62	0.00421182	Putative uncharacterized protein
LITE_CONTIG_6156	Tc02g006880	AT4G39250	-2.52	0.007265786	Predicted protein
3385_68416_m03036	Tc04g021580	AT3G24190	-2.47	0.003185736	Protein ABC1, mitochondrial, putative
LITE_CONTIG_3674	Tc08g002080	AT5G42760	-2.47	0.000898366	Predicted protein
LITE_CONTIG_3819	Tc05g005790	AT3G09600	-2.39	1.70E-05	Putative Myb-like protein G
LITE_CONTIG_2634	#N/A	#N/A	-2.31	0.003336477	#N/A
KAS8YM08FM1_	Tc00g055970	#N/A	-2.29	1.93E-05	Putative uncharacterized protein
LITE_CONTIG_3729	Tc09g004830	AT4G33010	-2.28	0.00740582	Glycine dehydrogenase [decarboxylating], mitochondrial
3623_68417_m04394	Tc09g002840	AT4G30950	-2.22	0.003677679	Omega-6 fatty acid desaturase, chloroplastic
LITE_CONTIG_3167	Tc08g008710	AT4G22110	-2.22	0.000176907	Alcohol dehydrogenase-like 5
KCAF3YK14FM1_	Tc03g030440	#N/A	-2.21	0.000408682	Predicted protein
KBF6YL24FM1_gi_45736089_dbj_BAD13114_1_	Tc03g015550	AT5G41110	-2.21	0.000898366	Putative uncharacterized protein
LITE_CONTIG_4309	Tc03g015550	AT3G26890	-2.19	0.000762152	Putative uncharacterized protein
KZOAAT_CL235Contig1_hypothetical	Tc00g073810	#N/A	-2.18	0.000524006	Putative Cytidylate kinase
KBB15YJ16FM1_hypothetical	Tc08g006760	AT4G33740	-2.15	0.00970896	Putative Predicted protein
KAT9YK17FM1_gi_453249_emb_CAA54612_1_	Tc01g003670	AT2G18570	-2.13	0.001665774	Anthocyanidin 3-O-glucosyltransferase 5

LITE_CONTIG_366	Tc02g014240	AT3G21890	-2.10	0.000762152	Predicted protein
KAP3YP20FM1_hypothetical	Tc02g030800	AT1G63840	-2.09	0.004438975	Putative uncharacterized protein
LITE_CONTIG_2848	Tc04g016570	AT5G01820	-2.09	0.003117947	CBL-interacting serine/threonine-protein kinase 11
LITE_CONTIG_619	Tc09g004830	AT4G33010	-2.08	0.002086516	Glycine dehydrogenase [decarboxylating], mitochondrial
KCL4YH09FM1_gi_28973337_gb_AAO63993_1_	Tc03g030500	AT1G72030	-2.08	0.001493718	Putative uncharacterized protein
KAC5YI11FM1_hypothetical	Tc08g007190	AT1G27480	-2.07	0.001205893	Putative Group XV phospholipase A2
KAP6YG19FM1_gi_89274163_gb_ABD65602_1_	Tc09g034710	AT1G06980	-2.06	0.014009834	Predicted protein
150068418_m06565	Tc03g017860	AT5G52900	-2.06	0.000408682	Putative uncharacterized protein
3374_68418_m02669	Tc09g029150	AT5G22830	-2.01	0.000500135	At5g22830
LITE_CONTIG_1080	Tc06g008920	AT1G17620	-2.00	0.007859438	Predicted protein
KAQ6YG23FM1_hypothetical	Tc09g020550	AT1G03870	-2.00	0.026551188	Fasciclin-like arabinogalactan protein 9
KAS2YA10FM1_hypothetical	Tc10g002710	AT5G13820	-2.00	0.016695517	Putative Telomere-binding protein 1
KAC3YD10FM1_hypothetical	#N/A	AT1G58170	-1.99	0.004297116	#N/A
LITE_CONTIG_2038	Tc03g012450	AT5G60920	-1.98	0.037040021	Protein COBRA
KAT7YD17FM1_gi_31711894_gb_AAP68303_1_	Tc04g028040	AT3G16560	-1.96	0.000500135	Probable protein phosphatase 2C 40
LITE_CONTIG_4616	Tc08g000160	AT5G42830	-1.95	0.000762152	Putative Uncharacterized acetyltransferase At3g50280
KAC12YG09FM1_ATP_sulfurylase	Tc08g003410	AT1G19920	-1.95	0.001205893	Bifunctional 3'-phosphoadenosine 5'-phosphosulfate synthase 2
LITE_CONTIG_4711	Tc05g028380	AT4G32890	-1.95	0.014696385	Putative GATA transcription factor 9
LITE_CONTIG_2382	Tc08g005980	AT5G65480	-1.94	0.000685043	Putative uncharacterized protein
LITE_CONTIG_4017	Tc09g029150	AT5G22830	-1.93	0.000495079	At5g22830
LITE_CONTIG_5436	Tc00g054430	AT5G22390	-1.92	0.000474427	Putative uncharacterized protein
LITE_CONTIG_4797	Tc01g015280	AT3G12120	-1.91	0.001017133	Omega-6 fatty acid desaturase, endoplasmic reticulum isozyme 2
LITE_CONTIG_492	Tc04g014810	#N/A	-1.88	0.00049603	Ribulose bisphosphate carboxylase small chain, chloroplastic
140368418_m00026	Tc05g002870	AT5G01210	-1.86	0.010459917	Putative BAHD acyltransferase DCR
KAS3YE03FM1_gi_89111876_gb_ABD60710_1_	Tc01g006270	AT5G67390	-1.86	0.001867512	Predicted protein
LITE_CONTIG_3466	Tc01g026880	AT2G06850	-1.85	0.000240051	Probable xyloglucan endotransglucosylase/hydrolase protein B
LITE_CONTIG_3137	Tc01g031210	AT3G62660	-1.85	0.042024332	Probable galacturonosyltransferase-like 7
KAT11YG17FM1_F12K11_2	Tc01g015000	AT1G06690	-1.84	0.00726844	Uncharacterized oxidoreductase At1g06690, chloroplastic
3316_68414_m01366	Tc06g019410	AT1G11860	-1.84	0.000685043	Aminomethyltransferase, mitochondrial
LITE_CONTIG_2288	Tc09g031930	AT5G59350	-1.83	0.00830391	Predicted protein

LITE_CONTIG_956	Tc02g012830	AT5G42420	-1.83	0.001205893	Putative Uncharacterized membrane protein At1g06890
LITE_CONTIG_321	Tc00g064020	AT1G70000	-1.81	0.000865416	Putative uncharacterized protein
LITE_CONTIG_2498	Tc03g023740	AT2G38740	-1.81	0.00053484	Dynamin-related protein 1E
318268415_m01710	Tc02g009700	AT2G15020	-1.80	0.003261717	Putative uncharacterized protein
LITE_CONTIG_1907	Tc09g016590	AT4G30320	-1.79	0.000500135	Pathogenesis-related protein PR-1
KAP3YN24FM1_	Tc09g013430	#N/A	-1.77	0.003606794	V-type proton ATPase 21 kDa proteolipid subunit
KBA9YK07FM1_hypothetical	Tc01g040660	AT2G41900	-1.75	0.01765395	Zinc finger CCCH domain-containing protein 30
LITE_CONTIG_6253	Tc02g010700	AT4G33580	-1.74	0.000865416	Carbonic anhydrase
KBA5YH08FM1_	Tc02g008760	#N/A	-1.73	0.001017133	LAG1 longevity assurance homolog 2
2409_68414_m03681	Tc06g012490	AT2G34590	-1.72	0.004103041	Pyruvate dehydrogenase E1 component subunit beta
KBB13YL03FM1_Protein	Tc03g004330	AT1G12030	-1.71	0.005118364	Predicted protein
KAS6YB22FM1_Lotus	Tc07g009610	#N/A	-1.71	0.004209879	Predicted protein
1779_68414_m03681	Tc06g012490	AT1G30120	-1.70	0.002239433	Pyruvate dehydrogenase E1 component subunit beta
KAQ10YB15FM1_hypothetical	Tc00g064020	AT1G70000	-1.70	0.000914396	Putative uncharacterized protein
LITE_CONTIG_3646	#N/A	AT1G65870	-1.68	0.001268659	#N/A
KBB12YK21FM1_gi_19033168_gb_AAL83562_1_AF468689_1	Tc05g025090	AT1G08550	-1.68	0.005710418	Violaxanthin de-epoxidase, chloroplastic
KCAF1YD08FM1_hypothetical	Tc03g013700	AT3G48280	-1.68	0.003606794	Cytochrome P450 71A25
1776_68417_m01948	Tc03g018470	AT5G44620	-1.66	0.00156536	Putative Cytochrome P450 93A1
KAV11YK19FM1_putative	Tc06g016050	AT4G04930	-1.65	0.001205893	Sphingolipid delta(4)-desaturase DES1
LITE_CONTIG_1762	Tc06g019410	AT1G11860	-1.64	0.003483416	Aminomethyltransferase, mitochondrial
3763_68418_m02874	Tc03g021450	AT5G24380	-1.64	0.004599223	Metal-nicotianamine transporter YSL3
LITE_CONTIG_294	Tc05g027980	#N/A	-1.64	0.031078681	Putative uncharacterized protein
LITE_CONTIG_3702	Tc10g002710	AT5G13820	-1.64	0.000500135	Putative Telomere-binding protein 1
LITE_CONTIG_364	Tc02g034960	#N/A	-1.63	0.001205893	Putative Probable NADP-dependent oxidoreductase P1
KAT3YF10FM1_hypothetical	Tc08g009510	AT1G77990	-1.63	0.001619461	Low affinity sulfate transporter 3
KBF10YH14FM1_	Tc04g000590	#N/A	-1.62	0.003472839	Putative DNA repair protein UVH3
3387_68414_m05928	Tc06g003200	AT1G52510	-1.62	0.002585655	Putative Haloalkane dehalogenase
KBF4YO17FM1_Protein	Tc04g023670	AT3G04910	-1.61	0.002063393	Serine/threonine-protein kinase WNK1
LKBE3YJ22FM1_gi_21436243_gb_AAM51260_1_	Tc02g012200	AT3G09580	-1.61	0.005539549	Amine oxidase, putative
LITE_CONTIG_45	Tc10g002920	#N/A	-1.60	0.00297846	Ribulose bisphosphate carboxylase/oxygenase activase 1, chloroplastic
KAV15YL16FM1_protein	Tc09g007820	AT5G56890	-1.60	0.037503718	Putative Serine/threonine-protein kinase PBS1
LITE_CONTIG_1462	Tc02g027260	#N/A	-1.58	0.001205893	Mitogen-activated protein kinase 17

1866_68416_m02391	Tc09g013770	AT3G18830	-1.58	0.004599223	Polyol transporter 5
LKBE4YE16FM1_	Tc05g003240	#N/A	-1.58	0.014259799	Predicted protein (Fragment)
KCAA3YF17FM1_hypothetical	Tc00g052000	AT5G40230	-1.58	0.006524351	Putative Auxin-induced protein 5NG4
KAQ9YD07FM1_Zinc	Tc01g040660	AT2G41900	-1.57	0.002106615	Zinc finger CCCH domain-containing protein 30
LITE_CONTIG_1386	Tc04g010980	#N/A	-1.56	0.018599241	Hypothetical protein
3253_68417_m02008	Tc05g020770	AT4G12800	-1.56	0.007647574	Photosystem I reaction center subunit XI, chloroplastic
LITE_CONTIG_4963	Tc00g028940	#N/A	-1.55	0.008689034	Kinase, putative
LITE_CONTIG_6343	Tc00g052720	AT1G78780	-1.55	0.000500135	Pathogen-related protein
LITE_CONTIG_3379	Tc09g013770	AT3G18830	-1.55	0.00450327	Polyol transporter 5
KAV1YG23_gi_116325966_gb_ABJ98584_1_	Tc06g011970	AT1G31175	-1.53	0.003569221	Predicted protein
LITE_CONTIG_477	Tc00g007220	AT1G55480	-1.53	0.004499883	Predicted protein
LITE_CONTIG_298	Tc02g002780	#N/A	-1.53	0.040305303	Putative Glutathione-regulated potassium-efflux system protein kefB
LITE_CONTIG_6248	Tc01g033910	AT4G00870	-1.53	0.021124088	Putative Transcription factor MYC2
KAT5YK22FM1_gi_30102668_gb_AAP21252_1_	Tc07g009610	AT1G53300	-1.52	0.005539549	Predicted protein
KAS1YE18_gi_29824239_gb_AAP04080_1_	Tc00g020270	AT5G61290	-1.52	0.037977291	Putative Flavin-containing monooxygenase FMO GS-OX3
1109_	Tc07g003570	#N/A	-1.52	0.015948037	1-acyl-sn-glycerol-3-phosphate acyltransferase
KAC11YB08FM1_hypothetical	Tc02g032180	AT4G09350	-1.51	0.002411981	Predicted protein
LITE_CONTIG_2507	Tc07g016580	AT3G12780	-1.51	0.010080591	Phosphoglycerate kinase, chloroplastic
KBB14YK23FM1_hypothetical	Tc06g014190	AT5G45820	-1.50	0.003185736	CBL-interacting protein kinase 5
1415_68414_m04947	Tc08g005260	AT1G42970	-1.50	0.016602567	Glyceraldehyde-3-phosphate dehydrogenase B, chloroplastic
3468_68418_m07361	Tc09g030000	AT5G58770	-1.50	0.046109756	Predicted protein (Fragment)
3682_68418_m07777	Tc00g014550	AT5G61960	-1.49	0.001302402	Predicted protein
KCL2YA19FM1_gi_13430518_gb_AAK25881_1_AF360171_1	Tc09g014050	AT1G50020	-1.49	0.001548988	Putative uncharacterized protein
1471_68417_m02008	Tc05g020770	AT4G12800	-1.49	0.015948037	Photosystem I reaction center subunit XI, chloroplastic
KBA9YB23FM1_RNA_binding	Tc05g005830	#N/A	-1.49	0.004297116	Predicted protein
KAS10YB15FM1_	Tc01g029550	#N/A	-1.48	0.003683796	Putative uncharacterized protein
KCAF7YB20FM1_hypothetical	Tc08g008920	AT1G78230	-1.48	0.000778457	Putative uncharacterized protein
LITE_CONTIG_3036	Tc02g024820	AT1G70640	-1.48	0.012723784	Putative uncharacterized protein
LKBE5YO13FM1_non_intrinsic	Tc06g019040	AT4G04770	-1.47	0.015725857	UPF0051 protein ABCI8, chloroplastic
LITE_CONTIG_897	Tc01g033120	AT3G62030	-1.47	0.006997547	Peptidyl-prolyl cis-trans isomerase CYP20-3, chloroplastic
LITE_CONTIG_1321	Tc01g009900	AT5G64940	-1.47	0.036472855	Putative Uncharacterized protein sll1770
KBA9YL02FM1_	Tc03g014080	#N/A	-1.46	0.006217635	Putative Protein cbbY
LKBE15YL06FM1_	Tc09g029620	#N/A	-1.46	0.007940346	Hypothetical protein
1413_68414_m03681	Tc06g012490	AT2G34590	-1.46	0.004426103	Pyruvate dehydrogenase E1

					component subunit beta
LITE_CONTIG_6794	Tc00g045720	#N/A	-1.46	0.001113783	Putative uncharacterized protein
LITE_CONTIG_3707	Tc08g013360	AT4G09890	-1.46	0.007224288	Predicted protein
3020_68414_m01622	Tc02g032160	AT1G13820	-1.44	0.020254608	Predicted protein
KAV7YE01FM1_amino	Tc00g059490	AT5G23810	-1.44	0.000707973	Probable amino acid permease 7
KAS7YF07FM1_ADP_glucose	Tc08g002410	AT1G27680	-1.44	0.007739669	Glucose-1-phosphate adenylyltransferase large subunit, chloroplastic/amyloplastic
LITE_CONTIG_3959	Tc06g008830	AT3G13800	-1.43	0.01904138	Putative hydrolase C777.06c
LITE_CONTIG_3016	Tc09g026080	AT1G51340	-1.43	0.019604988	Aluminum-activated citrate transporter
KBB4YI01FM1_	Tc01g035850	#N/A	-1.43	0.013552938	Probable anion transporter 2, chloroplastic
LITE_CONTIG_4916	Tc06g019090	AT1G61900	-1.43	0.001302402	Uncharacterized GPI-anchored protein At1g61900
LITE_CONTIG_910	Tc05g023470	AT5G17780	-1.42	0.007895851	Uncharacterized protein At5g17780.2
LITE_CONTIG_3409	Tc01g019860	AT5G48300	-1.42	0.016292463	Glucose-1-phosphate adenylyltransferase small subunit, chloroplastic
KAT7YC12FM1_hypothetical	Tc03g003620	AT2G41120	-1.41	0.007862252	Putative Protein of unknown function DUF309
2556_68418_m08381	Tc08g002400	AT5G66460	-1.41	0.005506931	Mannan endo-1,4-beta-mannosidase 7
1368_68416_m03065	Tc09g010180	AT3G24420	-1.40	0.011448102	Putative Sigma factor sigB regulation protein rsbQ
LITE_CONTIG_3453	Tc05g004630	AT5G59370	-1.40	0.03336163	Actin
CL550Contig1_hypothetical	Tc06g020240	AT2G20680	-1.40	0.004321031	Mannan endo-1,4-beta-mannosidase 6
LITE_CONTIG_763	Tc03g014080	AT3G48420	-1.40	0.024605103	Putative Protein cbbY
LITE_CONTIG_1346	Tc06g014720	AT1G53240	-1.39	0.002170064	Malate dehydrogenase, mitochondrial
LITE_CONTIG_84	Tc02g032180	#N/A	-1.39	0.003185736	Predicted protein
LITE_CONTIG_5969	Tc01g033560	AT2G46830	-1.39	0.014769544	Putative Protein LHY
LITE_CONTIG_4564	Tc05g005830	#N/A	-1.38	0.001205893	Predicted protein
KCAA6YK20FM1_Nitrogen_fixing	Tc09g017700	AT4G25910	-1.38	0.002228096	NifU-like protein 3, chloroplastic
KCAK3YH09FM1_gi_113594658_dbj_BAF18532_1_	Tc00g022740	#N/A	-1.37	0.011607182	Putative uncharacterized protein
LITE_CONTIG_1627	Tc09g002910	AT2G24270	-1.37	0.004103041	NADP-dependent glyceraldehyde-3-phosphate dehydrogenase
KAV9YO03FM1_	#N/A	#N/A	-1.37	0.033405324	#N/A
KAT1YP18FM1_Protein	Tc04g028650	AT5G27930	-1.37	0.001205893	Probable protein phosphatase 2C 73
KAC4YJ03FM1_	Tc01g040660	#N/A	-1.37	0.007086693	Zinc finger CCCH domain-containing protein 30
219868418_m00225	Tc05g005760	AT5G02830	-1.36	0.032574988	Pentatricopeptide repeat-containing protein At5g02830, chloroplastic
LITE_CONTIG_3969	Tc03g016190	AT5G51110	-1.36	0.001093078	Putative pterin-4-alpha-carbinolamine dehydratase
LITE_CONTIG_1638	Tc01g033650	AT5G24470	-1.36	0.004209879	Putative Two-component response regulator-like PRR95
1568_68416_m04623	Tc09g005980	AT3G43540	-1.36	0.003185736	Putative uncharacterized protein
LITE_CONTIG_679	Tc08g002400	AT5G66460	-1.35	0.000559715	Mannan endo-1,4-beta-mannosidase 7

LITE_CONTIG_464	Tc09g013990	#N/A	-1.35	0.020669821	Putative Uncharacterized protein At2g34460, chloroplastic
KAT10YC17FM1_hypothetical	Tc03g019680	AT5G52060	-1.34	0.006549561	Protein binding protein, putative
KCAK1YP24FM1_gi_431955_emb_CAA53925_1_	Tc01g026990	AT3G54660	-1.34	0.004138744	Glutathione reductase, chloroplastic (Fragment)
KAV6YF03FM1_harpin	Tc04g025830	AT3G23400	-1.34	0.00450327	Predicted protein
3687_68417_m00343	Tc04g028130	AT4G02510	-1.34	0.000344259	Predicted protein
KAV10YK15FM1_hypothetical	Tc03g010610	#N/A	-1.33	0.029580421	Putative uncharacterized protein
3661_68415_m04339	Tc03g023580	AT2G35390	-1.33	0.020194763	Ribose-phosphate pyrophosphokinase 1
LITE_CONTIG_2226	Tc01g033650	AT5G24470	-1.33	0.003261717	Putative Two-component response regulator-like PRR95
LITE_CONTIG_1132	Tc00g025170	AT5G17170	-1.33	0.004599223	Electron transporter, putative
3490_68418_m06467	Tc03g019660	AT5G52100	-1.33	0.020967934	Putative uncharacterized protein
LITE_CONTIG_5541	Tc03g019930	AT5G52570	-1.33	0.033358875	Beta-carotene hydroxylase
KBB7YG21FM1_	Tc00g043280	#N/A	-1.32	0.014626124	Sucrose-phosphate synthase 2
KAQ1YB04RM2_hypothetical	Tc08g000080	AT5G47820	-1.32	0.003472839	Putative Chromosome-associated kinesin KIF4A
KAC2YL23FM1_hypothetical	Tc02g001990	AT2G26500	-1.32	0.007895851	Cytochrome b6-f complex subunit 7 (Fragment)
KCAA4YB21FM1_Esterase/lipase/thioesterase	Tc01g013210	AT5G23530	-1.32	0.017239107	Putative Probable gibberellin receptor GID1L3
KBA3YL02FM1_	Tc06g012880	#N/A	-1.31	0.000685043	3-oxoacyl-[acyl-carrier-protein] synthase I, chloroplastic
KAP3YP10FM1_	Tc03g019680	#N/A	-1.31	0.002585655	Protein binding protein, putative
LITE_CONTIG_1048	Tc09g002140	AT4G32260	-1.30	0.002819082	Putative uncharacterized protein
KAV10YC19FM1_sialyltransferase_like	Tc09g028000	AT1G08280	-1.30	0.003606794	Sialyltransferase-like protein
LITE_CONTIG_5080	Tc04g000940	AT5G14760	-1.30	0.012837511	Putative L-aspartate oxidase 1
LITE_CONTIG_1644	Tc02g006400	AT4G38970	-1.30	0.015948037	Probable fructose-bisphosphate aldolase 1, chloroplastic
KAC3YB09FM1_	Tc01g019630	#N/A	-1.29	0.001619461	Putative uncharacterized protein
KBF5YN03FM1_	Tc02g012930	#N/A	-1.29	0.037626083	Sphingoid long-chain bases kinase 1
306568414_m08923	Tc02g001340	AT1G76680	-1.29	0.005601122	12-oxophytodienoate reductase 1
LITE_CONTIG_2041	Tc09g029070	AT1G08200	-1.29	0.019994016	Putative Bifunctional polymyxin resistance protein arnA
LITE_CONTIG_940	Tc06g009920	AT5G20540	-1.28	0.011354619	Protein Brevis radix-like 2
KBB2YO05FM1_putative	Tc08g010020	AT1G21980	-1.28	0.002755892	Phosphatidylinositol-4-phosphate 5-kinase 1
LITE_CONTIG_4127	Tc01g002070	AT4G38960	-1.28	0.001205893	Putative uncharacterized protein
LITE_CONTIG_336	Tc03g021780	#N/A	-1.28	0.011810557	Putative Two-component response regulator-like APRR5
LKBE10YK05FM1_PAP	Tc00g045350	AT1G51110	-1.27	0.013680019	Probable plastid-lipid-associated protein 12, chloroplastic
KAS7YA09FM1_membrane_associated	Tc02g034980	AT2G32480	-1.27	0.005392485	Putative zinc metalloprotease slr1821
CL276Contig1_gi_60547937_gb_AAX23932_1_	Tc03g019230	AT5G51920	-1.27	0.00194669	Predicted protein

KAQ3YF07FM1_hypothetical	Tc00g082000	AT5G49800	-1.26	0.002991806	Putative uncharacterized protein
3787	Tc01g005060	AT5G67030	-1.26	0.033358875	Zeaxanthin epoxidase, chloroplastic
305968418_m00188	Tc05g004950	AT5G02540	-1.26	0.021538955	Putative Retinol dehydrogenase 12
LITE_CONTIG_1633	Tc02g023650	AT1G23740	-1.25	0.003184445	Quinone oxidoreductase-like protein At1g23740, chloroplastic
LITE_CONTIG_899	Tc05g003260	AT3G54050	-1.25	0.014047023	Fructose-1,6-bisphosphatase, chloroplastic
LITE_CONTIG_168	Tc02g000080	#N/A	-1.23	0.005131007	Hypothetical protein
KAS9YH06FM1_hypothetical	Tc01g037180	AT2G45600	-1.23	0.005539549	Putative Probable gibberellin receptor GID1L3
LITE_CONTIG_1752	Tc09g022300	AT3G45140	-1.23	0.03336163	Lipoxygenase 2, chloroplastic
LITE_CONTIG_333	Tc02g012050	#N/A	-1.23	0.012723784	Hypothetical protein
KBB13YJ01FM1_hypothetical	Tc01g026030	#N/A	-1.23	0.004138744	Putative uncharacterized protein
1592_68417_m00418	Tc01g029360	AT4G03100	-1.23	0.003089788	Gtpase activating protein, putative
KAT6YI19FM1_	Tc02g008760	#N/A	-1.23	0.002257368	LAG1 longevity assurance homolog 2
KAT4YG17FM1_ubiquitin	Tc09g034320	AT2G30100	-1.23	0.005054528	Pentatricopeptide repeat-containing protein At2g30100, chloroplastic
KCAK2YJ17FM1_hypothetical	Tc08g008620	AT2G22430	-1.23	0.02410881	Putative Homeobox-leucine zipper protein ATHB-16
KAS3YL19FM1_	Tc05g003260	#N/A	-1.23	0.010133718	Fructose-1,6-bisphosphatase, chloroplastic
KBA7YG03FM1_	Tc02g026850	#N/A	-1.23	0.00942074	Putative uncharacterized protein
KBB13YG12FM1_Short_chain	Tc05g004950	AT2G37540	-1.22	0.042160148	Putative Retinol dehydrogenase 12
LITE_CONTIG_4874	Tc06g020610	AT2G35760	-1.22	0.048462398	UPF0497 membrane protein 5
LITE_CONTIG_3428	Tc03g002290	AT4G20360	-1.22	0.0094698	Elongation factor Tu, chloroplastic
CL338Contig1_hypothetical	Tc02g024990	AT1G67840	-1.20	0.004624992	ATP binding protein, putative
KAT5YH06FM1_gi_23296404_gb_AAN13110_1_	Tc08g002790	AT1G75460	-1.20	0.032755686	ATP-dependent peptidase, putative
KAT7YN23FM1_mitochondrial	Tc04g030560	AT3G06483	-1.20	0.000500135	Putative [Pyruvate dehydrogenase [lipoamide]] kinase, mitochondrial
KBA8YN10FM1_hypothetical	Tc03g003970	AT1G73760	-1.19	0.004321031	Ring finger containing protein, putative
KCAA8YD10FM1_s	#N/A	#N/A	-1.18	0.02410881	#N/A
KAS1YO21_hypothetical	Tc00g022620	AT4G27600	-1.17	0.002820841	Putative Uncharacterized sugar kinase slr0537
115868414_m01045	Tc05g031470	AT1G09340	-1.17	0.042760685	Uncharacterized protein At1g09340, chloroplastic
1512_68416_m06048	Tc01g026990	AT3G54660	-1.17	0.020205278	Glutathione reductase, chloroplastic (Fragment)
LITE_CONTIG_798	Tc09g003580	AT2G25737	-1.17	0.010506138	Predicted protein
LITE_CONTIG_6070	Tc03g025170	AT4G00150	-1.17	0.005722463	Putative uncharacterized protein
LITE_CONTIG_309	Tc03g002290	#N/A	-1.16	0.019139778	Elongation factor Tu, chloroplastic
3791_68415_m03069	Tc09g012720	AT2G25620	-1.15	0.016313324	Probable protein phosphatase 2C 27
KBB9YI24FM1_	Tc10g004240	#N/A	-1.15	0.008588654	PsbP-like protein 1, chloroplastic

LITE_CONTIG_153	Tc09g002840	#N/A	-1.15	0.006478197	Omega-6 fatty acid desaturase, chloroplastic
KCAA4YL09FM1_RWP_RK	Tc02g003500	AT4G35270	-1.15	0.00444274	Putative uncharacterized protein
LITE_CONTIG_5239	Tc10g004240	AT3G55330	-1.15	0.001828202	PsbP-like protein 1, chloroplastic
KAT4YF02FM1_gi_28059039_gb_AAO29981_1_	Tc04g023960	AT2G32040	-1.15	0.002686468	Transporter, putative
LITE_CONTIG_40	Tc01g026990	#N/A	-1.14	0.019198037	Glutathione reductase, chloroplastic (Fragment)
KAV4YI24FM1_	Tc05g006140	#N/A	-1.14	0.010459917	Putative MLO-like protein 3
KBF10YF09FM1_gi_10178160_dbj_BAB11572_1_	Tc03g017540	AT5G24120	-1.14	0.040305303	RNA polymerase sigma factor rpoD, putative
2460_68414_m00643	Tc04g019650	AT1G06130	-1.13	0.005758938	Probable hydroxyacylglutathione hydrolase 2, chloroplast
LITE_CONTIG_2364	Tc03g030450	AT1G32100	-1.13	0.049718673	Isoflavone reductase homolog
LITE_CONTIG_3056	Tc00g055980	AT3G09890	-1.13	0.037818724	Ankyrin repeat-containing protein, putative
KBB14YF22FM1_lipoxygenase	Tc09g022300	AT3G45140	-1.13	0.042760685	Lipoxygenase 2, chloroplastic
LITE_CONTIG_302	Tc08g008420	#N/A	-1.12	0.013552938	Putative Photosystem II oxygen evolving complex protein PsbP
KAC1YM07RM1_	Tc01g032280	#N/A	-1.12	0.015948037	NifU-like protein 1, chloroplastic
CL90Contig1_unnamed	Tc07g007410	AT5G54470	-1.12	0.005968783	Transcription factor, putative
KBB16YN08FM1_	Tc00g090040	#N/A	-1.11	0.016730927	1,4-alpha-glucan-branching enzyme 2, chloroplastic/amyloplastic
KAS2YD02FM1_hypothetical	Tc08g008750	AT1G78290	-1.11	0.003569221	Serine/threonine-protein kinase SAPK2
KAV13YG23FM1_hypothetical	Tc04g020470	AT4G13930	-1.11	0.040673244	Serine hydroxymethyltransferase 1
LITE_CONTIG_1281	Tc06g019400	AT1G62250	-1.11	0.020254608	Predicted protein
LITE_CONTIG_1530	Tc08g007670	AT5G43150	-1.11	0.024899244	Putative uncharacterized protein
KAS8YE19FM1_gi_22136218_gb_AAM91187_1_	Tc05g031240	AT5G26820	-1.10	0.007265786	Putative uncharacterized protein
KCAA3YA19FM1_gi_29029030_gb_AAO64894_1_	Tc01g012920	AT5G08720	-1.09	0.046631977	Putative polyketide cyclase
KAT4YK01FM1_gi_51971729_dbj_BAD44529_1_	Tc10g004450	AT3G12345	-1.09	0.030742549	Putative uncharacterized protein
KAV12YH05FM1_hypothetical	Tc02g032850	AT1G14030	-1.09	0.04588655	Probable ribulose-1,5 bisphosphate carboxylase/oxygenase large subunit N-methyltransferase, chloroplastic
KBB12YE21FM1_unknown	Tc10g001320	AT3G55990	-1.09	0.005054528	Putative uncharacterized protein
KBB2YI05FM1_hypothetical	Tc09g028530	AT2G27680	-1.09	0.00508481	Putative uncharacterized protein
LITE_CONTIG_2062	Tc05g012720	AT5G36790	-1.09	0.042641388	Putative Phosphoglycolate phosphatase
LITE_CONTIG_6270	Tc06g020450	AT4G16490	-1.08	0.003742223	Ubiquitin-protein ligase, putative
LITE_CONTIG_2459	Tc02g024760	AT1G70610	-1.07	0.048611343	ABC transporter B family

					member 26, chloroplastic
303968414_m06216	Tc05g028040	AT1G54500	-1.07	0.004428323	Putative Rubredoxin
KAT4YJ08FM1_uroporphyrinogen	Tc08g000970	AT2G26540	-1.07	0.046678996	Uroporphyrinogen-III synthase, chloroplastic
KBB4YH22FM1_gi_21536786_gb_AAM61118_1_	Tc00g090770	AT1G03600	-1.07	0.047072745	Predicted protein
LITE_CONTIG_2657	Tc09g029070	#N/A	-1.07	0.03117183	Putative Bifunctional polymyxin resistance protein arnA
LITE_CONTIG_4534	Tc03g024510	AT5G47240	-1.07	0.005539549	Nudix hydrolase 8
KAS2YJ22FM1_hypothetical	Tc07g000040	AT4G26470	-1.07	0.002142052	Probable calcium-binding protein CML21
351668416_m05855	Tc05g007520	AT3G53130	-1.07	0.019251787	Putative Cytochrome P450 97B2
LITE_CONTIG_2832	Tc02g002050	AT2G15960	-1.07	0.034388543	Putative uncharacterized protein
KBB11YE10FM1_hypothetical	Tc00g004350	AT5G57180	-1.06	0.004499883	Putative Protein CHLOROPLAST IMPORT APPARATUS 2
LKBE1YF12RM1_	Tc07g012900	#N/A	-1.06	0.034790244	Peptide methionine sulfoxide reductase msrB
KBA8YF02FM1_gi_3193301_gb_AAC19285_1_	Tc04g028130	AT4G02510	-1.06	0.033786456	Predicted protein
LITE_CONTIG_3131	Tc02g024160	AT1G60800	-1.06	0.003774691	Probable LRR receptor-like serine/threonine-protein kinase At2g23950
KAT3YM06FM1_mitochondrial	Tc04g030560	AT3G06483	-1.06	0.001396423	Putative [Pyruvate dehydrogenase [lipoamide]] kinase, mitochondrial
KAV15YM14FM1_gi_20197057_gb_AAC06162_2_	Tc01g037140	AT2G45630	-1.05	0.005131007	Putative Glyoxylate reductase
CL222Contig1_	Tc06g019780	#N/A	-1.05	0.01904138	Protein STRUBBELIG-RECEPTOR FAMILY 8
KBB13YD18FM1_hypothetical	Tc09g025090	AT2G32560	-1.04	0.009156526	F-box protein At2g32560
3378	Tc03g006290	#N/A	-1.04	0.04631044	Predicted protein
3809_68418_m02666	Tc02g003980	AT5G22800	-1.04	0.03336163	Alanyl-tRNA synthetase
LITE_CONTIG_3255	Tc06g012490	AT2G34590	-1.04	0.012723784	Pyruvate dehydrogenase E1 component subunit beta
KBA6YC07FM1_hypothetical	Tc00g017680	AT5G58380	-1.03	0.020942199	CBL-interacting protein kinase 18
KBB10YN20FM1_	Tc03g005260	#N/A	-1.03	0.004438975	Mitogen-activated protein kinase 9
KCAE7YA22FM1_hypothetical	Tc06g010490	AT1G54050	-1.03	0.019612424	17.4 kDa class III heat shock protein
LITE_CONTIG_1303	Tc00g090890	AT4G03400	-1.03	0.029976358	Probable indole-3-acetic acid-amido synthetase GH3.5
LITE_CONTIG_313	Tc00g054600	#N/A	-1.03	0.020194763	Putative F-box protein At4g12560
KAV1YB13_hypothetical	Tc10g009100	#N/A	-1.03	0.046821036	Putative uncharacterized protein
LITE_CONTIG_1766	Tc08g013910	AT1G22850	-1.03	0.005126138	Putative TVP38/TMEM64 family membrane protein slr0305
LKBE7YD05FM1_hypothetical	Tc01g024980	AT1G06240	-1.02	0.006720155	Putative Uncharacterized protein HI_0077
LITE_CONTIG_1098	Tc04g030750	AT1G48320	-1.02	0.003733279	Putative uncharacterized protein
KAC1YJ07_gi_21700871_gb_AAM70559_1	Tc04g004520	AT3G27050	-1.02	0.003938386	Predicted protein

_					
LITE_CONTIG_72	Tc02g023680	AT3G01500	-1.02	0.018830029	Carbonic anhydrase, chloroplastic
3662_68418_m02258	Tc04g022680	AT5G19010	-1.02	0.001653826	Mitogen-activated protein kinase 16
185668414_m03629	Tc02g034500	AT1G29700	-1.02	0.037680061	Putative uncharacterized protein
LITE_CONTIG_3020	Tc10g006740	AT3G10420	-1.02	0.043103964	Putative Uncharacterized protein ycf45
KAT2YB11FM1_	Tc00g059940	#N/A	-1.01	0.014919981	Cytochrome c1-1, heme protein, mitochondrial
LITE_CONTIG_3417	Tc00g037170	AT1G24160	-1.01	0.003185736	Predicted protein
LITE_CONTIG_4612	Tc08g008920	#N/A	-1.00	0.021592643	Putative uncharacterized protein
LITE_CONTIG_4596	Tc00g004350	AT4G25990	-1.00	0.003261717	Putative Protein CHLOROPLAST IMPORT APPARATUS 2
KAV1YE16RM1_	Tc02g024820	#N/A	-1.00	0.048928387	Putative uncharacterized protein
KAV4YA08FM1_	Tc07g011590	#N/A	-1.00	0.010814412	Mitogen-activated protein kinase 19
KBB2YL10FM1_putative	Tc05g004950	AT5G02540	-1.00	0.040883336	Putative Retinol dehydrogenase 12
KBB5YL12FM1_gi_21439_emb_CAA44055_1_	Tc00g059940	AT5G40810	-0.99	0.01245988	Cytochrome c1-1, heme protein, mitochondrial
KAA2YE12FM1_gi_30725272_gb_AAP37658_1_	Tc09g002180	AT2G25430	-0.99	0.03224542	Putative clathrin assembly protein At2g25430
KAP4YD05FM1_amino	Tc08g006680	AT5G49630	-0.99	0.002614995	Amino acid permease 6
LITE_CONTIG_1846	Tc01g037820	AT2G45290	-0.99	0.033405324	Transketolase, chloroplastic
KAP1YL12FM1_gi_110735769_dbj_BAE99862_1_	Tc01g037410	AT2G45440	-0.99	0.005914874	Dihydrodipicolinate synthase 2, chloroplastic
KAS10YL04FM1_hypothetical	Tc02g011760	AT5G64170	-0.99	0.019139778	Putative uncharacterized protein
KAQ2YE18FM1_	Tc00g035350	#N/A	-0.98	0.008405914	Predicted protein
KAP10YF13FM1_hypothetical	Tc05g000440	AT5G16520	-0.98	0.001165286	Predicted protein
LITE_CONTIG_2750	Tc01g001630	AT4G36040	-0.98	0.005438069	Chaperone protein dnaJ 11, chloroplastic
LKBE14YL12FM1_	Tc01g000440	#N/A	-0.98	0.016754061	Putative uncharacterized protein
KAT10YE17FM1_Vitis	Tc02g011760	#N/A	-0.98	0.032991123	Putative uncharacterized protein
KAQ3YC23FM1_hypothetical	Tc04g000510	AT3G28050	-0.97	0.007895851	Putative Auxin-induced protein 5NG4
KCAA10YF01FM1_gi_27363402_gb_AAO11620_1_	Tc04g003280	AT3G27110	-0.97	0.028958138	Metalloendopeptidase, putative
KAS10YD21FM1_hypothetical	Tc10g001050	#N/A	-0.97	0.022896725	Putative Serine/threonine-protein phosphatase 6 regulatory ankyrin repeat subunit B
LITE_CONTIG_5727	Tc01g019860	AT5G48300	-0.97	0.003677679	Glucose-1-phosphate adenylyltransferase small subunit, chloroplastic
KBB8YD21FM1_	Tc09g007330	#N/A	-0.97	0.003523236	Putative Ankyrin repeat-containing protein At3g12360
KBF4YA04FM1_hypothetical	Tc09g004450	AT5G11700	-0.97	0.025303384	Putative uncharacterized protein
353868418_m04675	Tc04g015190	AT5G38660	-0.97	0.010368304	Putative Thylakoid membrane protein slr0575
KAQ4YD11FM1_puta	Tc01g005720	AT4G37270	-0.96	0.019809289	Probable cadmium/zinc-

tive					transporting ATPase HMA1, chloroplastic
KAA15YN03FM1_protein	Tc05g019270	AT3G02750	-0.96	0.039900131	Probable protein phosphatase 2C 33
KCAE2YH20FM1_hypothetical	Tc01g002780	AT5G66450	-0.96	0.026626013	Putative uncharacterized protein
KAP12YJ10FM1_hypothetical	Tc06g004190	AT4G24840	-0.95	0.010056599	Putative Conserved oligomeric Golgi complex subunit 2
KAQ5YE10FM1_	#N/A	#N/A	-0.95	0.034056614	#N/A
LITE_CONTIG_3678	Tc01g033600	AT2G46820	-0.95	0.01021683	Thylakoid membrane phosphoprotein 14 kDa, chloroplastic
LITE_CONTIG_3214	Tc01g033670	AT1G01090	-0.95	0.003185736	Pyruvate dehydrogenase E1 component subunit alpha
LITE_CONTIG_5976	Tc08g011220	AT5G54160	-0.95	0.00450327	Caffeic acid 3-O-methyltransferase
KAV7YA12FM1_cytochrome	Tc04g011400	#N/A	-0.94	0.028635907	Hypothetical protein
LITE_CONTIG_3962	Tc00g050720	AT5G49720	-0.94	0.041968031	Endoglucanase 25
2296_68418_m01674	Tc08g010000	AT5G14320	-0.94	0.019848659	30S ribosomal protein S13, chloroplastic
KBF10YF10FM1_glutamate	Tc01g000350	AT4G35290	-0.94	0.030136594	Glutamate receptor 3.6
KAS2YE10FM1_gi_22137054_gb_AAM91372_1_	Tc02g003750	AT4G35250	-0.94	0.019604988	Uncharacterized protein ycf39
KCAA9YC20FM1_predicted	#N/A	#N/A	-0.94	0.027948575	#N/A
149568417_m05398	Tc02g012490	AT4G38225	-0.93	0.005539549	Predicted protein (Fragment)
KBA6YO20FM1_gi_125553992_gb_EAY99597_1_	Tc09g010970	AT5G20070	-0.93	0.007647574	Nudix hydrolase 19, chloroplastic
KAQ1YH02FM2_D8_sphingolipid	Tc09g004930	AT2G46210	-0.93	0.041124544	Putative Fatty acid desaturase 3
LITE_CONTIG_4959	Tc00g071610	AT3G02830	-0.93	0.005601122	Zinc finger CCCH domain-containing protein ZFN-like
KBB2YA12FM1_hypothetical	Tc05g027880	AT5G28150	-0.93	0.039900131	Predicted protein
LITE_CONTIG_1525	Tc02g024970	AT1G24580	-0.92	0.01132734	Protein binding protein, putative
KAP2YK16FM1_transport	Tc01g029600	AT3G62980	-0.92	0.00508481	Protein TRANSPORT INHIBITOR RESPONSE 1
LITE_CONTIG_6829	Tc00g017750	AT2G01670	-0.92	0.012170307	Nudix hydrolase 17, mitochondrial
KAT11YF16FM1_hypothetical	Tc10g003150	AT3G55440	-0.91	0.005539549	Triosephosphate isomerase, cytosolic
KAP10YF15FM1_hypothetical	Tc01g037800	AT1G02000	-0.91	0.006327318	UDP-glucuronate 4-epimerase 3
KBA8YA07FM1_hypothetical	Tc04g030150	AT3G17240	-0.91	0.006414838	Dihydrolipoyl dehydrogenase 1, mitochondrial
2586	Tc01g003400	#N/A	-0.91	0.035302716	Putative uncharacterized protein
1419_68416_m00023	Tc04g009010	AT3G01180	-0.91	0.011221883	Granule-bound starch synthase 2, chloroplastic/amyloplastic
LITE_CONTIG_5731	Tc01g038370	AT4G00050	-0.90	0.015221966	Transcription factor UNE10
LITE_CONTIG_5710	Tc08g011200	AT1G44350	-0.90	0.047917363	IAA-amino acid hydrolase ILR1-like 6
LITE_CONTIG_1283	Tc00g087820	AT5G19760	-0.90	0.007160609	Putative mitochondrial 2-oxoglutarate/malate carrier protein
LITE_CONTIG_1529	Tc03g000690	AT5G40240	-0.90	0.027130582	Putative Auxin-induced protein 5NG4

KBF9YI15FM1_Putative	Tc02g033060	AT1G14150	-0.90	0.015449922	Putative oxygen evolving enhancer protein 3, identical
gi_148726911_gb_EH057661_1_EH057661	Tc01g005120	AT5G42930	-0.89	0.021889293	Putative uncharacterized protein
KAA11YH14FM1_serine_glyoxylate	Tc09g023620	AT2G13360	-0.89	0.034731082	Serine--glyoxylate aminotransferase
KAV3YH14FM1_hypothetical	Tc04g023250	AT5G19260	-0.89	0.033029947	Putative uncharacterized protein
KBB2YL14FM1_gi_26450615_dbj_BAC42419_1_	Tc06g007350	AT3G21360	-0.89	0.02257264	Clavaminate synthase-like protein At3g21360
KCAK5YG18FM1_gi_110740923_dbj_BAE98557_1_	Tc03g006190	AT5G43940	-0.88	0.02234347	Alcohol dehydrogenase class-3
LITE_CONTIG_4301	Tc09g002180	AT2G25430	-0.87	0.004928964	Putative clathrin assembly protein At2g25430
LITE_CONTIG_4374	Tc10g006320	AT3G46510	-0.87	0.004438975	U-box domain-containing protein 14
LITE_CONTIG_3653	Tc02g012560	AT2G17350	-0.87	0.007851467	Putative uncharacterized protein
KAC10YD21FM1_gi_21700805_gb_AAM70526_1_	Tc02g004620	AT2G20920	-0.87	0.016695517	Putative uncharacterized protein
KBB11YE18FM1_	Tc05g005600	#N/A	-0.87	0.005968783	Zinc finger protein, putative
KCAA1YG12FM1_hypothetical	Tc00g014160	AT2G34860	-0.87	0.007224288	Putative uncharacterized protein
LITE_CONTIG_3258	Tc04g029150	AT4G16146	-0.87	0.039565558	Putative uncharacterized protein
116268416_m01224	Tc00g068180	AT3G10230	-0.87	0.005601122	Lycopene beta cyclase, chloroplastic/chromoplastic
KAV8YN22FM1_hypothetical	Tc07g010370	AT1G53380	-0.86	0.045406952	Predicted protein
KAV12YE11FM1_phototropin	Tc09g032890	AT3G45780	-0.86	0.01275571	Phototropin-1
KCAF3YF06FM1_	Tc00g033920	#N/A	-0.86	0.042969884	Fiber protein Fb17 (Fragment)
KBB10YH01FM1_	Tc01g003540	AT5G66720	-0.86	0.015558762	Probable protein phosphatase 2C 80
KBA9YH07FM1_gi_115502269_sp_Q3EDJ0_NAP4_ARATH	Tc01g025410	AT1G03905	-0.86	0.009544057	ABC transporter I family member 19
1200_	Tc01g029630	AT4G03180	-0.86	0.019594345	Putative uncharacterized protein
KAT8YP21FM1_dehydroascorbate	Tc04g015010	AT5G16710	-0.86	0.042969884	Glutathione S-transferase DHAR1, mitochondrial
KCAK4YK05FM1_photoregulatory	Tc01g017730	AT2G32950	-0.85	0.019139778	E3 ubiquitin-protein ligase COP1
KCAA3YH18FM1_	Tc03g014260	#N/A	-0.85	0.014632494	Ubiquinone biosynthesis protein coq-8, putative
LITE_CONTIG_6811	Tc09g025090	AT2G26850	-0.85	0.007895851	F-box protein At2g32560
267468415_m05677	Tc01g037070	AT2G45660	-0.85	0.029422501	MADS-box protein SOC1
LITE_CONTIG_902	Tc09g012860	AT2G12462	-0.85	0.013161326	Predicted protein
LKBE1YE10FM1_hypothetical	Tc09g023710	#N/A	-0.85	0.018387334	Hypothetical protein
LITE_CONTIG_1302	Tc00g090890	AT4G03400	-0.84	0.037503718	Probable indole-3-acetic acid-amido synthetase GH3.5
KCAE5YH17FM1_hypothetical	Tc09g011440	AT2G25490	-0.84	0.039900131	EIN3-binding F-box protein 1
KAQ11YC12FM1_gi_19785_emb_CAA45152_1_	Tc06g019200	AT4G04640	-0.84	0.039534177	ATP synthase gamma chain, chloroplastic
KCAA3YF23FM1_hypothetical	Tc02g006360	AT2G21290	-0.84	0.005946559	30S ribosomal protein S31, mitochondrial
1619_68414_m05351	Tc04g030150	AT1G48030	-0.84	0.046203644	Dihydrolipoyl dehydrogenase 1, mitochondrial

LITE_CONTIG_1010	Tc05g002470	AT5G01410	-0.83	0.033405324	Probable pyridoxal biosynthesis protein PDX1
2857_68415_m05090	Tc05g014300	AT2G41220	-0.83	0.046917667	Ferredoxin-dependent glutamate synthase, chloroplastic
LITE_CONTIG_3932	Tc03g031220	AT1G31420	-0.83	0.003472839	LRR receptor-like serine/threonine-protein kinase FEI 1
LITE_CONTIG_6201	Tc01g025670	AT1G04020	-0.83	0.006178107	Brca1 associated ring domain, putative
1269_68416_m05638	Tc01g000350	AT3G51480	-0.83	0.025398989	Glutamate receptor 3.6
LITE_CONTIG_6625	Tc03g013580	AT5G50200	-0.83	0.01643745	Putative uncharacterized protein
LITE_CONTIG_1021	Tc01g029040	AT2G48070	-0.82	0.014125869	Predicted protein
KBA9YG11FM1_hypothetical	Tc02g000380	AT4G19690	-0.81	0.007257553	Zinc transporter 1
KBF1YC16RM1_gi_113537115_dbj_BAF09498_1_	Tc09g030290	AT5G58870	-0.81	0.042760685	Cell division protease ftsH homolog 9, chloroplastic
LITE_CONTIG_2444	Tc02g007320	AT4G34640	-0.80	0.037825898	Squalene synthase
KAT1YD05_	Tc03g011090	#N/A	-0.80	0.006478197	Putative uncharacterized protein
LITE_CONTIG_1680	Tc03g000670	AT3G48040	-0.80	0.046203644	Rac-like GTP-binding protein 3
LITE_CONTIG_6151	Tc07g010830	AT1G78630	-0.80	0.028329439	50S ribosomal protein L13, chloroplastic
LITE_CONTIG_1637	Tc07g010370	AT1G53380	-0.80	0.021096476	Predicted protein
KBA8YD02FM1_peroxidase	Tc01g001190	AT5G42180	-0.80	0.028203815	Peroxidase 64
LITE_CONTIG_5549	Tc03g024200	AT5G47110	-0.80	0.043616523	Light harvesting-like protein 3
LITE_CONTIG_2297	Tc06g019200	AT4G04640	-0.79	0.026551188	ATP synthase gamma chain, chloroplastic
LITE_CONTIG_2311	Tc02g034100	AT2G02710	-0.79	0.040383839	Protein TWIN LOV 1
KAC5YB15FM1_hypothetical	Tc00g018080	AT5G54160	-0.79	0.008689034	Caffeic acid 3-O-methyltransferase
276068414_m08041	Tc00g001530	AT1G69870	-0.79	0.013279764	Putative Peptide transporter PTR1
LITE_CONTIG_1254	Tc04g021580	AT3G24190	-0.79	0.045333504	Protein ABC1, mitochondrial, putative
LITE_CONTIG_707	Tc00g054320	AT2G27470	-0.78	0.005722463	Predicted protein
LITE_CONTIG_559	Tc10g001240	AT3G56010	-0.78	0.007185769	Putative uncharacterized protein
LITE_CONTIG_788	Tc03g026520	AT1G63000	-0.78	0.048309905	Probable rhamnose biosynthetic enzyme 1
LITE_CONTIG_3834	Tc10g003310	AT5G59370	-0.77	0.00726844	Actin
312168416_m03167	Tc01g020420	AT3G25480	-0.77	0.012723784	Chitinase, putative
KAT9YE02FM1_SOUL	Tc05g003200	AT2G37970	-0.77	0.034388543	Putative Heme-binding-like protein At3g10130, chloroplastic
KCAA9YB21FM1_	Tc03g005260	#N/A	-0.77	0.011354619	Mitogen-activated protein kinase 9
KAQ6YE06FM1_hypothetical	Tc09g010180	AT3G24420	-0.77	0.045872391	Putative Sigma factor sigB regulation protein rsbQ
KAT3YK22FM1_gi_88010848_gb_ABD38862_1_	Tc01g017080	AT3G22210	-0.77	0.006217635	At3g22210
LITE_CONTIG_4715	Tc04g017510	AT2G43710	-0.76	0.007895851	Acyl-[acyl-carrier-protein] desaturase, chloroplastic
LITE_CONTIG_2445	Tc05g007470	AT3G53140	-0.76	0.032238868	Putative Caffeic acid 3-O-methyltransferase
KBF8YH15FM1_gi_20453407_gb_AAM19942_1_	Tc02g028230	AT4G37200	-0.76	0.042024332	Putative Thiol:disulfide interchange protein txlA homolog
CL579Contig1_interfe	Tc08g007570	AT1G27760	-0.76	0.018562138	Putative Interferon-related

ron_related					developmental regulator 1
LITE_CONTIG_5628	Tc01g019030	AT3G07720	-0.76	0.020796428	Nitrile-specifier protein 5
LITE_CONTIG_6470	Tc02g005560	AT2G21170	-0.75	0.032461907	Triosephosphate isomerase, chloroplastic
LITE_CONTIG_3568	Tc09g011440	AT2G25490	-0.75	0.017289271	EIN3-binding F-box protein 1
LITE_CONTIG_4193	Tc02g034460	AT1G68660	-0.75	0.03586944	ATP-dependent Clp protease adapter protein clpS
KCL1YG23FM1_hypothetical	Tc02g014650	AT4G03260	-0.75	0.014948947	Protein phosphatase 1 regulatory subunit, putative
114168414_m00661	Tc06g014670	AT1G06250	-0.75	0.04503792	Predicted protein
LKBE14YJ16FM1_	Tc01g003740	#N/A	-0.75	0.019139778	Geranylgeranyl pyrophosphate synthase, chloroplastic
2293_68414_m00523	Tc02g030620	AT1G05190	-0.75	0.021458817	50S ribosomal protein L6, chloroplastic
KAA13YA16FM1_gi_7267558_emb_CAB78039_1_	Tc02g007880	AT1G22930	-0.75	0.034056614	Putative uncharacterized protein
KAP3YN21FM1_CONSTANS	Tc04g019390	AT2G31380	-0.74	0.034212479	Salt tolerance-like protein
1250_68418_m08330	Tc01g001360	AT5G66120	-0.73	0.036522972	3-dehydroquinate synthase
LITE_CONTIG_2477	Tc01g004960	AT4G37000	-0.73	0.013251137	Red chlorophyll catabolite reductase, chloroplastic
LITE_CONTIG_4777	Tc01g002950	AT1G01050	-0.73	0.048928387	Soluble inorganic pyrophosphatase
KBF6YP03FM1_	Tc01g014740	#N/A	-0.73	0.034177888	Hypothetical protein
LITE_CONTIG_2250	Tc05g003220	AT5G01090	-0.73	0.032238868	Putative Agglutinin-2
LITE_CONTIG_4190	Tc04g003850	ATCG00490	-0.73	0.020254608	Ribulose bisphosphate carboxylase large chain
LITE_CONTIG_6202	Tc09g035000	AT5G58375	-0.73	0.005722463	Putative uncharacterized protein
LITE_CONTIG_1360	Tc02g004890	AT1G68010	-0.73	0.039900131	Glycerate dehydrogenase
LITE_CONTIG_2650	Tc02g013590	AT1G50320	-0.73	0.007392602	Thioredoxin-X, chloroplastic
KAS8YM22FM1_hypothetical	Tc07g015240	AT1G17220	-0.72	0.046107612	Translation initiation factor IF-2, chloroplastic
2639_68416_m06367	Tc05g016260	AT5G36170	-0.72	0.034056614	Putative Peptide chain release factor 2
LITE_CONTIG_2546	Tc06g017320	AT1G65800	-0.72	0.029422501	Putative serine/threonine-protein kinase receptor
LITE_CONTIG_1605	Tc02g021280	AT1G68490	-0.72	0.025657927	Putative uncharacterized protein
LITE_CONTIG_1776	Tc09g010360	AT1G08510	-0.72	0.033053265	Myristoyl-acyl carrier protein thioesterase, chloroplastic
KAS3YN16FM1_hypothetical	Tc04g025230	AT1G06620	-0.72	0.016730927	1-aminocyclopropane-1-carboxylate oxidase homolog 1
KAA13YC10FM1_	Tc02g007320	#N/A	-0.72	0.043616523	Squalene synthase
LITE_CONTIG_906	Tc00g074390	AT1G74780	-0.71	0.022900489	Putative uncharacterized protein
LITE_CONTIG_6166	Tc03g006490	AT1G19000	-0.71	0.036497444	Transcription factor Myb1
KAC6YA18FM1_gi_51970326_dbj_BAD43855_1_	Tc01g032510	AT1G02475	-0.71	0.040344917	Putative Streptomyces cyclase/dehydrase
LITE_CONTIG_6122	Tc04g024540	AT5G43750	-0.71	0.037680061	Putative uncharacterized protein
LITE_CONTIG_5524	Tc07g011910	AT3G14690	-0.71	0.036841753	Secologanin synthase
LITE_CONTIG_6821	Tc06g001760	AT3G15810	-0.71	0.047077862	Putative UPF0706 protein At5g01750
KAS1YF02_geranylgeranyl	Tc01g003740	AT4G36810	-0.71	0.019139778	Geranylgeranyl pyrophosphate synthase, chloroplastic
KAC1YE11FM1_P5CS1	Tc10g002590	AT3G55610	-0.71	0.018212464	Delta-1-pyrroline-5-carboxylate synthase
KAQ4YG05FM1_	Tc01g037060	#N/A	-0.70	0.019808927	Putative Lysophosphatidylcholine acyltransferase 2
LITE_CONTIG_3536	Tc03g031270	AT5G45390	-0.70	0.016410821	ATP-dependent Clp protease

					proteolytic subunit 4, chloroplastic
LITE_CONTIG_3548	Tc04g022630	AT5G35630	-0.69	0.04588655	Glutamine synthetase leaf isozyme, chloroplastic
KAC14YO09FM1_DRP	Tc06g000130	AT1G10290	-0.68	0.042760685	Dynamin-2B
KAA9YP01FM1_hypothetical	Tc05g005370	AT5G02620	-0.68	0.032238868	Ankyrin repeat-containing protein At5g02620
KCAK5YP23FM1_hypothetical	Tc03g015110	#N/A	-0.67	0.030285032	Putative Squamosa promoter-binding-like protein 13
KAP24YM03FM1_similar	Tc03g025410	AT1G62620	-0.67	0.022494614	Flavin-containing monooxygenase FMO GS-OX5
KCL2YM10FM1_hypothetical	Tc01g030500	AT4G02440	-0.67	0.013124095	Phytochrome A-associated F-box protein
LITE_CONTIG_2962	Tc02g005580	AT2G21180	-0.65	0.038107974	Predicted protein
KAC7YJ06FM1_gi_110742199_dbj_BAE99026_1_	Tc09g027630	AT2G27290	-0.65	0.03302795	Putative uncharacterized protein
LITE_CONTIG_1408	Tc05g003880	AT2G37770	-0.65	0.015948037	Putative Aldose reductase
2205_68415_m05938	Tc01g030640	AT2G47590	-0.65	0.042969884	Blue-light photoreceptor PHR2
LITE_CONTIG_1729	Tc10g016790	AT3G56460	-0.64	0.026933333	Putative Quinone oxidoreductase-like protein 2 homolog
LITE_CONTIG_2489	Tc10g003640	AT2G39570	-0.64	0.049579415	Predicted protein
LITE_CONTIG_3661	Tc03g029000	AT4G23890	-0.64	0.048928387	Putative uncharacterized protein
LITE_CONTIG_289	Tc04g003350	#N/A	-0.64	0.024605103	30S ribosomal protein S21, chloroplastic (Fragment)
KCAA6YH08FM1_hypothetical	Tc09g014810	AT5G11270	-0.63	0.028456877	Putative uncharacterized protein
LITE_CONTIG_631	Tc05g002860	AT5G01220	-0.63	0.024344877	Putative GDP-mannose-dependent alpha-mannosyltransferase
114068415_m03469	Tc09g032990	AT2G28550	-0.63	0.026626013	Ethylene-responsive transcription factor RAP2-7
KAA10YG10FM1_tubby_like	Tc01g003050	AT2G18280	-0.63	0.032182525	Tubby-like F-box protein 5
1306_68415_m03258	Tc10g012550	AT2G27110	-0.62	0.024826266	Protein FAR1-RELATED SEQUENCE 3
LITE_CONTIG_2808	Tc05g018880	AT3G57990	-0.62	0.017128102	Putative uncharacterized protein
LITE_CONTIG_3519	Tc05g018100	AT3G57800	-0.62	0.046821036	Putative Transcription factor bHLH60
LITE_CONTIG_2374	Tc03g029230	AT4G16580	-0.62	0.029953273	Putative Probable protein phosphatase 2C 55
LITE_CONTIG_1844	Tc04g028670	AT4G16070	-0.62	0.029557448	Predicted protein
LITE_CONTIG_5653	Tc01g034790	AT4G01100	-0.61	0.024605103	Putative Mitochondrial substrate carrier family protein B
LITE_CONTIG_5527	Tc01g021120	AT4G13010	-0.61	0.032888617	Quinone-oxidoreductase homolog, chloroplastic
KAA14YK09FM1_gi_21700923_gb_AAM70585_1_	Tc08g001530	AT5G42660	-0.61	0.012915752	Predicted protein
KAC10YM15FM1_	Tc03g029210	#N/A	-0.61	0.031536622	Putative uncharacterized protein
KBB16YI24FM1_auxin	Tc03g029280	AT4G23980	-0.59	0.032520334	Auxin response factor 9
KAT4YJ18FM1_hypothetical	Tc01g029630	AT4G03180	-0.59	0.040520385	Putative uncharacterized protein
LITE_CONTIG_2828	Tc05g026170	AT5G28960	-0.59	0.034731082	Putative uncharacterized protein
LITE_CONTIG_6427	Tc06g009500	AT3G13690	-0.59	0.041595522	ATP binding protein, putative
LITE_CONTIG_2629	Tc01g025580	AT4G28570	-0.58	0.042969884	Electron carrier, putative

KCAA6YP05FM1_hypothetical	Tc02g016160	AT3G54680	-0.58	0.036841753	Putative uncharacterized protein
LITE_CONTIG_113	Tc04g017510	#N/A	-0.57	0.038389257	Acyl-[acyl-carrier-protein] desaturase, chloroplastic
LITE_CONTIG_5575	Tc04g026150	AT5G55240	-0.56	0.038294425	Caleosin
LITE_CONTIG_5393	Tc08g005050	AT1G20650	-0.56	0.04503792	Serine/threonine-protein kinase PBS1
KCAA3YO12FM1_hypothetical	Tc00g046680	AT2G18890	-0.56	0.036472855	Putative BRASSINOSTEROID INSENSITIVE 1-associated receptor kinase 1
KAV9YH19FM1_gi_107738038_gb_ABF83618_1_	Tc06g003100	AT3G15900	-0.55	0.037562875	Putative uncharacterized protein
LITE_CONTIG_5307	Tc02g033620	AT1G69760	-0.55	0.042870427	Putative uncharacterized protein
LITE_CONTIG_3001	Tc00g051020	AT1G43700	-0.54	0.04116838	Putative uncharacterized protein
KAC5YG13FM1_	Tc05g006430	AT2G37195	-0.54	0.024750539	Putative uncharacterized protein
KCL1YH20FM1_nucleic	Tc02g013060	AT5G08535	-0.52	0.042983647	Nucleic acid binding protein, putative
LITE_CONTIG_1894	Tc03g024380	AT4G17560	-0.51	0.049987291	50S ribosomal protein L19-1, chloroplastic
LITE_CONTIG_5893	Tc01g035400	AT5G52660	-0.49	0.029156759	Predicted protein
LITE_CONTIG_610	Tc01g034790	AT4G01100	-0.43	0.043616523	Putative Mitochondrial substrate carrier family protein B

Supplementary Table 8. ***Theobroma cacao*** **biotic stress genes differentially expressed due to endophyte** ***C. tropicale*** **inoculation in 1st and 2nd microarray experiments comparing E+ to E- leaves.**

Microarray Experiment	Tc Identifier	LogFC	Mapman BinCode	BinName
2nd	Tc09g020550	-2	10.5.1.1	cell wall.cell wall proteins.AGPs.AGP
2nd	Tc03g012560	0.56	10.5.5	cell wall.cell wall proteins.RGP
2nd	Tc07g001020	0.79	10.2.1	cell wall.cellulose synthesis.cellulose synthase
2nd	Tc00g046250	0.76	10.2.1	cell wall.cellulose synthesis.cellulose synthase
2nd	Tc05g022350	0.6	10.2.1	cell wall.cellulose synthesis.cellulose synthase
2nd	Tc03g012450	-1.98	10.2.2	cell wall.cellulose synthesis.COBRA
1st and 2nd	Tc08g002400	-0.86 and -1.41	10.6.2	cell wall.degradation.mannan-xylose-arabinose-fucose
2nd	Tc06g020240	-1.4	10.6.2	cell wall.degradation.mannan-xylose-arabinose-fucose
2nd	Tc09g016910	1.7	10.7	cell wall.modification
2nd	Tc01g026880	-1.85	10.7	cell wall.modification
2nd	Tc09g029070	-1.29	10.1.3	cell wall.precursor synthesis.AXS
2nd	Tc09g015240	1.21	10.1.6	cell wall.precursor synthesis.GAE
2nd	Tc01g037800	-0.91	10.1.6	cell wall.precursor synthesis.GAE
2nd	Tc06g000890	1.03	10.1.9	cell wall.precursor synthesis.MUR4
2nd	Tc04g014670	0.84	10.1.20	cell wall.precursor synthesis.phosphomannose isomerase
2nd	Tc04g001960	1.18	10.1.30.3	cell wall.precursor synthesis.sugar kinases.glucuronic acid kinase
2nd	Tc03g026520	-0.78	10.1.10	cell wall.precursor synthesis.UDP-glucose 4,6-dehydratase
1st	Tc03g026940	0.58	10.1.2	cell wall.precursor synthesis.UGE
2nd	Tc01g018340	1.36	10.1.2	cell wall.precursor synthesis.UGE
2nd	Tc10g003610	1.49	10.1.5	cell wall.precursor synthesis.UXS
2nd	Tc04g004840	0.82	17.1.3	hormone metabolism.abscisic acid.induced-regulated-responsive-activated
2nd	Tc01g005060	-1.26	17.1.1.1.1	hormone metabolism.abscisic acid.synthesis-degradation.synthesis.zeaxanthin epoxidase
2nd	Tc07g006560	1.52	17.2.3	hormone metabolism.auxin.induced-regulated-responsive-activated
2nd	Tc02g006210	1.23	17.2.3	hormone metabolism.auxin.induced-regulated-responsive-activated
2nd	Tc05g027610	0.77	17.2.3	hormone metabolism.auxin.induced-regulated-responsive-activated
2nd	Tc00g090890	-1.03	17.2.3	hormone metabolism.auxin.induced-regulated-responsive-activated
2nd	Tc01g029600	-0.92	17.2.2	hormone metabolism.auxin.signal transduction
2nd	Tc08g011200	-0.9	17.2.1	hormone metabolism.auxin.synthesis-degradation
2nd	Tc07g009900	1.25	17.3.2.2	hormone metabolism.brassinosteroid.signal transduction.BZR
2nd	Tc00g009520	0.68	17.3.2.2	hormone metabolism.brassinosteroid.signal transduction.BZR
2nd	Tc02g002540	0.78	17.3.1.2.99	hormone metabolism.brassinosteroid.synthesis-degradation.sterols.other
2nd	Tc05g031040	1.22	17.5.3	hormone metabolism.ethylene.induced-regulated-responsive-activated

2nd	Tc01g030440	0.76	17.5.3	hormone metabolism.ethylene.induced-regulated-responsive-activated
2nd	Tc04g017610	0.76	17.5.3	hormone metabolism.ethylene.induced-regulated-responsive-activated
1st	Tc08g010270	-0.76	17.5.1	hormone metabolism.ethylene.synthesis-degradation
2nd	Tc01g017710	1.52	17.5.1	hormone metabolism.ethylene.synthesis-degradation
2nd	Tc10g002860	0.59	17.5.1	hormone metabolism.ethylene.synthesis-degradation
2nd	Tc04g025230	-0.72	17.5.1	hormone metabolism.ethylene.synthesis-degradation
2nd	Tc01g019030	-0.76	17.7.3	hormone metabolism.jasmonate.induced-regulated-responsive-activated
2nd	Tc02g001340	-1.29	17.7.1.5	hormone metabolism.jasmonate.synthesis-degradation.12-Oxo-PDA-reductase
1st	Tc01g020090	0.57	17.7.1.2	hormone metabolism.jasmonate.synthesis-degradation.lipoxygenase
2nd	Tc09g022300	-1.23	17.7.1.2	hormone metabolism.jasmonate.synthesis-degradation.lipoxygenase
2nd	Tc09g031660	1.15	26.4.1	misc.beta 1,3 glucan hydrolases.glucan endo-1,3-beta-glucosidase
2nd	Tc01g014030	0.87	26.4.1	misc.beta 1,3 glucan hydrolases.glucan endo-1,3-beta-glucosidase
1st	Tc05g005680	-1.99	26.9	misc.glutathione S transferases
2nd	Tc00g041460	1.77	26.9	misc.glutathione S transferases
2nd	Tc01g001190	-0.8	26.12	misc.peroxidases
2nd	Tc09g008760	1.18	29.5	protein.degradation
2nd	Tc02g002560	0.98	29.5	protein.degradation
2nd	Tc01g035310	0.71	29.5	protein.degradation
2nd	Tc00g008140	0.58	29.5	protein.degradation
2nd	Tc04g003280	-0.97	29.5	protein.degradation
2nd	Tc08g002790	-1.2	29.5	protein.degradation
2nd	Tc01g002550	1.57	29.5.9	protein.degradation.AAA type
2nd	Tc01g002650	1.39	29.5.9	protein.degradation.AAA type
2nd	Tc09g030290	-0.81	29.5.7	protein.degradation.metalloprotease
2nd	Tc02g034980	-1.27	29.5.7	protein.degradation.metalloprotease
2nd	Tc08g001140	0.81	29.5.5	protein.degradation.serine protease
2nd	Tc03g031270	-0.7	29.5.5	protein.degradation.serine protease
2nd	Tc02g031310	0.75	29.5.11	protein.degradation.ubiquitin
2nd	Tc01g006440	1.42	29.5.11.4.5.2	protein.degradation.ubiquitin.E3.BTB/POZ Cullin3.BTB/POZ
2nd	Tc04g023340	0.8	29.5.11.4.5.2	protein.degradation.ubiquitin.E3.BTB/POZ Cullin3.BTB/POZ
2nd	Tc08g005400	2.1	29.5.11.4.2	protein.degradation.ubiquitin.E3.RING
2nd	Tc01g030430	1.98	29.5.11.4.2	protein.degradation.ubiquitin.E3.RING
2nd	Tc09g034100	1.69	29.5.11.4.2	protein.degradation.ubiquitin.E3.RING
2nd	Tc01g007370	1.32	29.5.11.4.2	protein.degradation.ubiquitin.E3.RING
2nd	Tc02g004940	0.81	29.5.11.4.2	protein.degradation.ubiquitin.E3.RING
2nd	Tc06g010970	0.76	29.5.11.4.2	protein.degradation.ubiquitin.E3.RING
2nd	Tc00g085480	0.7	29.5.11.4.2	protein.degradation.ubiquitin.E3.RING
2nd	Tc01g027040	0.67	29.5.11.4.2	protein.degradation.ubiquitin.E3.RING

2nd	Tc02g009070	0.6	29.5.11.4.2	protein.degradation.ubiquitin.E3.RING
2nd	Tc09g010720	0.55	29.5.11.4.2	protein.degradation.ubiquitin.E3.RING
2nd	Tc05g005600	-0.87	29.5.11.4.2	protein.degradation.ubiquitin.E3.RING
2nd	Tc10g006320	-0.87	29.5.11.4.2	protein.degradation.ubiquitin.E3.RING
2nd	Tc02g024970	-0.92	29.5.11.4.2	protein.degradation.ubiquitin.E3.RING
2nd	Tc06g020450	-1.08	29.5.11.4.2	protein.degradation.ubiquitin.E3.RING
2nd	Tc03g003970	-1.19	29.5.11.4.2	protein.degradation.ubiquitin.E3.RING
2nd	Tc02g026850	-1.23	29.5.11.4.2	protein.degradation.ubiquitin.E3.RING
2nd	Tc05g005830	-1.49	29.5.11.4.2	protein.degradation.ubiquitin.E3.RING
2nd	Tc06g012620	0.76	29.5.11.4.3.3	protein.degradation.ubiquitin.E3.SCF.cullin
2nd	Tc10g004090	1.16	29.5.11.4.3.2	protein.degradation.ubiquitin.E3.SCF.FBOX
2nd	Tc02g003310	1.03	29.5.11.4.3.2	protein.degradation.ubiquitin.E3.SCF.FBOX
2nd	Tc08g011990	0.89	29.5.11.4.3.2	protein.degradation.ubiquitin.E3.SCF.FBOX
2nd	Tc01g012390	0.55	29.5.11.4.3.2	protein.degradation.ubiquitin.E3.SCF.FBOX
2nd	Tc07g011170	0.51	29.5.11.4.3.2	protein.degradation.ubiquitin.E3.SCF.FBOX
2nd	Tc01g003050	-0.63	29.5.11.4.3.2	protein.degradation.ubiquitin.E3.SCF.FBOX
2nd	Tc01g030500	-0.67	29.5.11.4.3.2	protein.degradation.ubiquitin.E3.SCF.FBOX
2nd	Tc09g011440	-0.84	29.5.11.4.3.2	protein.degradation.ubiquitin.E3.SCF.FBOX
2nd	Tc00g054600	-1.03	29.5.11.4.3.2	protein.degradation.ubiquitin.E3.SCF.FBOX
2nd	Tc09g025090	-1.04	29.5.11.4.3.2	protein.degradation.ubiquitin.E3.SCF.FBOX
2nd	Tc04g029320	0.69	29.5.11.20	protein.degradation.ubiquitin.proteasom
2nd	Tc04g012970	0.5	29.5.11.20	protein.degradation.ubiquitin.proteasom
2nd	Tc03g021500	0.6	29.5.11.1	protein.degradation.ubiquitin.ubiquitin
2nd	Tc09g034320	-1.23	29.5.11.1	protein.degradation.ubiquitin.ubiquitin
1st	Tc05g010340	-0.65	21.2.1	redox.ascorbate and glutathione.ascorbate
1st	Tc00g015640	-1.2	21.2.1	redox.ascorbate and glutathione.ascorbate
2nd	Tc04g015010	-0.86	21.2.1	redox.ascorbate and glutathione.ascorbate
1st and 2st	Tc01g026990	-1.56 and -1.34	21.2.2	redox.ascorbate and glutathione.glutathione
2nd	Tc05g003200	-0.77	21.3	redox.heme
1st	Tc05g024980	1.1	21.1	redox.thioredoxin
1st	Tc02g008880	0.47	21.1	redox.thioredoxin
1st and 2st	Tc08g011150	0.77 and 1.13	21.1	redox.thioredoxin
2nd	Tc03g008330	0.84	21.1	redox.thioredoxin
2nd	Tc02g013590	-0.73	21.1	redox.thioredoxin
2nd	Tc07g009610	-1.71	21.1	redox.thioredoxin
2nd	Tc09g033360	0.61	21.1.1	redox.thioredoxin.PDIL
2nd	Tc03g019360	1.99	27.3.3	RNA.regulation of transcription.AP2/EREBP, APETALA2/Ethylene-responsive element binding protein family
2nd	Tc02g034130	0.67	27.3.3	RNA.regulation of transcription.AP2/EREBP, APETALA2/Ethylene-responsive element binding protein family
2nd	Tc09g032990	-0.63	27.3.3	RNA.regulation of transcription.AP2/EREBP, APETALA2/Ethylene-responsive element binding protein

				family
2nd	Tc02g006270	0.89	27.3.35	RNA.regulation of transcription.bZIP transcription factor family
2nd	Tc04g019520	0.85	27.3.35	RNA.regulation of transcription.bZIP transcription factor family
2nd	Tc00g051020	-0.54	27.3.35	RNA.regulation of transcription.bZIP transcription factor family
2nd	Tc03g011090	-0.8	27.3.8	RNA.regulation of transcription.C2C2(Zn) DOF zinc finger family
2nd	Tc03g003050	1.93	27.3.25	RNA.regulation of transcription.MYB domain transcription factor family
2nd	Tc01g005710	1.5	27.3.25	RNA.regulation of transcription.MYB domain transcription factor family
2nd	Tc02g009470	1	27.3.25	RNA.regulation of transcription.MYB domain transcription factor family
2nd	Tc10g002710	-1.64	27.3.25	RNA.regulation of transcription.MYB domain transcription factor family
1st and 2nd	Tc03g006290	-0.62 and 1.04	27.3.26	RNA.regulation of transcription.MYB-related transcription factor family
1st and 2nd	Tc01g033560	-3.01 and -1.39	27.3.26	RNA.regulation of transcription.MYB-related transcription factor family
2nd	Tc02g013270	0.51	27.3.26	RNA.regulation of transcription.MYB-related transcription factor family
2nd	Tc01g035400	-0.49	27.3.26	RNA.regulation of transcription.MYB-related transcription factor family
2nd	Tc03g006490	-0.71	27.3.26	RNA.regulation of transcription.MYB-related transcription factor family
2nd	Tc05g005790	-2.39	27.3.26	RNA.regulation of transcription.MYB-related transcription factor family
2nd	Tc02g006880	-2.52	27.3.26	RNA.regulation of transcription.MYB-related transcription factor family
2nd	Tc06g004420	1.08	27.3.32	RNA.regulation of transcription.WRKY domain transcription factor family
2nd	Tc05g027100	0.94	27.3.32	RNA.regulation of transcription.WRKY domain transcription factor family
2nd	Tc01g005580	0.75	27.3.32	RNA.regulation of transcription.WRKY domain transcription factor family
2nd	Tc00g075710	1.42	16.8.1.21	secondary metabolism.flavonoids.anthocyanins.anthocyanin 5-aromatic acyltransferase
1st	Tc04g021630	-1.54	16.8.2	secondary metabolism.flavonoids.chalcones
2nd	Tc03g030450	-1.13	16.8.5.1	secondary metabolism.flavonoids.isoflavones.isoflavone reductase
2nd	Tc02g012200	-1.61	16.1.4	secondary metabolism.isoprenoids.carotenoids
2nd	Tc03g019930	-1.33	16.1.4.6	secondary metabolism.isoprenoids.carotenoids.carotenoid beta ring hydroxylase
2nd	Tc00g068180	-0.87	16.1.4.5	secondary metabolism.isoprenoids.carotenoids.lycopene beta cyclase
2nd	Tc05g025090	-1.68	16.1.4.21	secondary metabolism.isoprenoids.carotenoids.violaxanthin de-epoxidase
2nd	Tc01g003740	-0.75	16.1.1.10	secondary metabolism.isoprenoids.non-mevalonate pathway.geranylgeranyl pyrophosphate synthase
1st	Tc09g013970	-0.93	16.1.1.7	secondary metabolism.isoprenoids.non-mevalonate pathway.HDR
2nd	Tc07g005270	1.24	16.1.5	secondary metabolism.isoprenoids.terpenoids
1st	Tc00g092420	-0.61	16.1.3.3	secondary metabolism.isoprenoids.tocopherol biosynthesis.MPBQ/MSBQ methyltransferase
1st and 2nd	Tc05g002870	0.59 and -1.86	16.2	secondary metabolism.phenylpropanoids
2nd	Tc02g010600	1.47	16.2	secondary metabolism.phenylpropanoids

2nd	Tc09g034830	1.07	16.2	secondary metabolism.phenylpropanoids
2nd	Tc08g000160	-1.95	16.2	secondary metabolism.phenylpropanoids
2nd	Tc00g033030	1.16	16.1	secondary metabolism.simple phenols
2nd	Tc04g010150	0.84	30.3	signalling.calcium
2nd	Tc01g006470	0.72	30.3	signalling.calcium
2nd	Tc00g035530	0.55	30.3	signalling.calcium
2nd	Tc01g037060	-0.7	30.3	signalling.calcium
2nd	Tc06g000140	1.21	30.5	signalling.G-proteins
2nd	Tc08g000320	1.21	30.5	signalling.G-proteins
2nd	Tc02g002060	0.96	30.5	signalling.G-proteins
2nd	Tc01g039980	0.64	30.5	signalling.G-proteins
2nd	Tc05g019600	0.63	30.5	signalling.G-proteins
2nd	Tc09g005770	0.62	30.5	signalling.G-proteins
2nd	Tc03g000670	-0.8	30.5	signalling.G-proteins
2nd	Tc01g029360	-1.23	30.5	signalling.G-proteins
2nd	Tc08g008820	1.53	30.1	signalling.in sugar and nutrient physiology
2nd	Tc01g000350	-0.94	30.1	signalling.in sugar and nutrient physiology
2nd	Tc04g030560	-1.2	30.1.2	signalling.in sugar and nutrient physiology.pyruvate dehydrogenase kinase
2nd	Tc04g005150	1.57	30.11	signalling.light
2nd	Tc01g030640	-0.65	30.11	signalling.light
2nd	Tc02g034100	-0.79	30.11	signalling.light
2nd	Tc09g013990	-1.35	30.11	signalling.light
2nd	Tc04g024360	-3.21	30.11	signalling.light
2nd	Tc09g033020	1.37	30.6	signalling.MAP kinases
2nd	Tc07g011590	-1	30.6	signalling.MAP kinases
2nd	Tc04g022680	-1.02	30.6	signalling.MAP kinases
2nd	Tc03g005260	-1.03	30.6	signalling.MAP kinases
2nd	Tc02g027260	-1.58	30.6	signalling.MAP kinases
2nd	Tc08g010020	-1.28	30.4.1	signalling.phosphinositides.phosphatidylinositol-4-phosphate 5-kinase
2nd	Tc04g011050	0.99	30.2.16	signalling.receptor kinases.Catharanthus roseus-like RLK1
2nd	Tc04g011220	0.94	30.2.16	signalling.receptor kinases.Catharanthus roseus-like RLK1
2nd	Tc01g005800	0.61	30.2.16	signalling.receptor kinases.Catharanthus roseus-like RLK1
2nd	Tc05g003220	-0.73	30.2.19	signalling.receptor kinases.legume-lectin
2nd	Tc09g033180	0.69	30.2.3	signalling.receptor kinases.leucine rich repeat III
2nd	Tc07g014090	0.74	30.2.8.2	signalling.receptor kinases.leucine rich repeat VIII.VIII-2
2nd	Tc05g025560	0.67	30.2.11	signalling.receptor kinases.leucine rich repeat XI
2nd	Tc04g015880	1.09	30.2.99	signalling.receptor kinases.misc
2nd	Tc04g023350	0.82	30.2.99	signalling.receptor kinases.misc
2nd	Tc03g031220	-0.83	30.2.99	signalling.receptor kinases.misc
2nd	Tc02g024990	-1.2	30.2.99	signalling.receptor kinases.misc
2nd	Tc02g024160	-1.06	30.2.22	signalling.receptor kinases.proline extensin like

2nd	Tc07g009940	1.64	30.2.24	signalling.receptor kinases.S-locus glycoprotein like
2nd	Tc07g007460	0.62	30.2.24	signalling.receptor kinases.S-locus glycoprotein like
2nd	Tc06g017320	-0.72	30.2.24	signalling.receptor kinases.S-locus glycoprotein like
2nd	Tc03g019680	-1.34	20.2	stress.abiotic
1st	Tc03g020100	-1.06	20.2.2	stress.abiotic.cold
2nd	Tc09g030850	1.3	20.2.2	stress.abiotic.cold
2nd	Tc05g003500	1.12	20.2.2	stress.abiotic.cold
2nd	Tc02g033420	1.28	20.2.3	stress.abiotic.drought/salt
1st	Tc01g013950	2.34	20.2.1	stress.abiotic.heat
1st	Tc06g010450	0.73	20.2.1	stress.abiotic.heat
1st	Tc02g031250	0.69	20.2.1	stress.abiotic.heat
1st	Tc09g030680	-2.01	20.2.1	stress.abiotic.heat
1st	Tc03g018090	-2.28	20.2.1	stress.abiotic.heat
1st and 2nd	Tc02g032180	-0.76 and -1.51	20.2.1	stress.abiotic.heat
2nd	Tc08g003750	1.29	20.2.1	stress.abiotic.heat
2nd	Tc09g014710	1.29	20.2.1	stress.abiotic.heat
2nd	Tc09g029790	1.04	20.2.1	stress.abiotic.heat
2nd	Tc09g001600	0.67	20.2.1	stress.abiotic.heat
2nd	Tc06g015310	0.64	20.2.1	stress.abiotic.heat
2nd	Tc01g001630	-0.98	20.2.1	stress.abiotic.heat
2nd	Tc02g015010	2.01	20.2.4	stress.abiotic.touch/wounding
2nd	Tc02g015040	1.59	20.2.4	stress.abiotic.touch/wounding
2nd	Tc07g007270	1.52	20.2.4	stress.abiotic.touch/wounding
2nd	Tc03g013020	2.19	20.1.7	stress.biotic.PR-proteins
2nd	Tc00g051580	0.91	20.1.7	stress.biotic.PR-proteins
2nd	Tc05g028690	0.79	20.1.7	stress.biotic.PR-proteins
1st	Tc00g042540	2.37	20.1.7.6.1	stress.biotic.PR-proteins.proteinase inhibitors.trypsin inhibitor
2nd	Tc05g006140	-1.14	20.1.3.1	stress.biotic.signalling.MLO-like

Supplementary Table 9. Genes involved in nitrogen metabolism that were differentially expressed due to endophyte *C. tropicale* inoculation in the 1st and 2nd microarray experiments comparing E+ to E- leaves. This table contains a total of 115 genes, all of them classified as GO nitrogen compound metabolic. These genes were also classified in other nitrogen GO categories: cellular nitrogen compound biosynthetic process (23 genes), cellular nitrogen compound metabolic process (36 genes), and regulation of nitrogen compound metabolic process (52 genes).

Microarray Experiment	Tc Identifier	At Identifier	Log FC	Annotation
1st and 2nd	Tc09g029650	AT3G47340.1	1.99 and 0.89	Asparagine synthetase [glutamine-hydrolyzing]
1st and 2nd	Tc09g030280	AT2G29630.1	1.53 and 1.08	Phosphomethylpyrimidine synthase
2nd	Tc03g019360	AT5G51990	1.99	Dehydration-responsive element-binding protein 1D
2nd	Tc03g003050	AT1G68320	1.93	Predicted protein
2nd	Tc09g034330	AT3G51770	1.9	Ethylene-overproduction protein 1
2nd	Tc01g017710	AT1G05010	1.52	1-aminocyclopropane-1-carboxylate oxidase 1
2nd	Tc10g003610	AT2G28760	1.49	UDP-glucuronic acid decarboxylase 1
2nd	Tc02g010600	AT2G22570	1.47	Putative Uncharacterized isochorismatase family protein pncA
2nd	Tc09g033020	AT3G45640	1.37	Mitogen-activated protein kinase 3
2nd	Tc06g013590	AT4G18930	1.32	Cyclic phosphodiesterase
2nd	Tc01g039040	AT3G60390	1.31	Homeobox-leucine zipper protein HAT3
2nd	Tc03g020090	AT4G25630	1.22	Putative Fibrillarin
2nd	Tc06g000140	AT4G21130	1.21	Putative U3 small nucleolar RNA-interacting protein 2
2nd	Tc09g015240	AT4G30440	1.21	UDP-glucuronate 4-epimerase 1
1st	Tc06g018680	AT5G54770.1	1.2	Thiazole biosynthetic enzyme, chloroplastic
2nd	Tc05g005020	AT2G37500	1.2	Arginine biosynthesis bifunctional protein argJ
1st	Tc09g004470	AT2G19810.1	1.15	Zinc finger CCCH domain-containing protein 20
2nd	Tc05g007980	AT3G10040	1.15	Putative uncharacterized protein
2nd	Tc08g005530	AT3G21110	1.09	Phosphoribosylaminoimidazole-succinocarboxamide synthase, chloroplastic
2nd	Tc05g020320	AT3G13570	1.08	Serine/arginine rich splicing factor, putative
2nd	Tc01g034910	AT3G61620	1.06	Exosome complex exonuclease RRP41
2nd	Tc01g004540	AT2G03500	1.04	Putative uncharacterized protein
2nd	Tc09g021860	AT5G20730	1.04	Auxin response factor 2
2nd	Tc02g003310	AT1G25280	1.03	Tubby-like F-box protein 8
2nd	Tc06g000890	AT1G30620	1.03	UDP-arabinose 4-epimerase 1
2nd	Tc02g009470	AT4G37260	1	Putative Transcription factor MYB44
2nd	Tc07g008920	AT1G29900	0.99	Carbamoyl-phosphate synthase large chain
2nd	Tc03g014010	AT4G13940	0.98	Adenosylhomocysteinase 1

1st	Tc03g009540	AT3G01470.1	0.96	Putative uncharacterized protein
2nd	Tc05g009030	AT1G08360	0.96	60S ribosomal protein L10a-1
2nd	Tc05g027100	AT5G28650	0.94	Probable WRKY transcription factor 74
1st	Tc03g013820	AT4G24770.1	0.93	31 kDa ribonucleoprotein, chloroplastic
2nd	Tc02g006270	AT4G38900	0.89	DNA binding protein, putative
2nd	Tc01g028210	AT3G63250	0.87	Hypothetical protein
2nd	Tc04g019520	AT1G06070	0.85	Probable transcription factor PosF21
2nd	Tc05g010140	AT2G36310	0.83	Putative Uncharacterized protein C1683.06c
2nd	Tc00g015920	AT3G10690	0.82	DNA gyrase subunit A, chloroplastic/mitochondrial
1st	Tc01g013570	AT3G58610.2	0.81	Ketol-acid reductoisomerase, chloroplastic
2nd	Tc00g068210	AT5G14800	0.81	Pyrroline-5-carboxylate reductase
2nd	Tc06g009950	AT1G72830	0.81	Nuclear transcription factor Y subunit A-3
2nd	Tc05g015540	AT3G57080	0.8	Putative DNA-directed RNA polymerases I, II, and III subunit RPABC1
2nd	Tc04g017610	AT1G05710	0.76	Transcription factor bHLH113
2nd	Tc01g005580	AT2G23320	0.75	Putative Probable WRKY transcription factor 7
1st	Tc04g019410	AT3G48560.1	0.73	Acetolactate synthase 2, chloroplastic
1st	Tc04g004710	AT3G26900.3	0.71	Putative Shikimate kinase, chloroplastic
1st	Tc09g030140	AT2G29690.1	0.69	Anthranilate synthase component I-2, chloroplastic
2nd	Tc02g032660	AT1G26640	0.69	Putative Uncharacterized protein MJ0044
2nd	Tc01g026730	AT4G16430	0.67	Transcription factor bHLH3
2nd	Tc02g034130	AT3G25890	0.67	Putative Ethylene-responsive transcription factor ERF118
2nd	Tc01g013340	AT1G33420	0.64	PHD finger protein At1g33420
1st	Tc09g023530	AT3G10330.1	0.63	Transcription initiation factor IIB
2nd	Tc00g036580	AT3G28480	0.63	Prolyl 4-hydroxylase alpha subunit, putative
2nd	Tc09g005770	AT2G26300	0.62	Guanine nucleotide-binding protein alpha-1 subunit
1st	Tc03g019670	AT5G52100.1	0.58	Putative dihydrodipicolinate reductase 3, chloroplastic
2nd	Tc01g031870	AT2G47300	0.57	Putative uncharacterized protein
1st	Tc09g004460	AT3G53920.1	0.55	Putative uncharacterized protein
2nd	Tc02g013270	AT5G08520	0.51	Predicted protein
2nd	Tc07g011170	AT1G53320	0.51	Tubby-like F-box protein 7
1st	Tc02g019690	AT5G62000.3	0.48	Auxin response factor 2
1st	Tc02g013800	AT5G08280.1	0.41	Porphobilinogen deaminase, chloroplastic
2nd	Tc01g035400	AT5G52660	-0.49	Predicted protein
2nd	Tc03g029280	AT4G23980	-0.59	Auxin response factor 9
1st	Tc03g006290	AT1G18330.1	-0.62	Predicted protein
2nd	Tc05g018100	AT3G57800	-0.62	Putative Transcription factor bHLH60
2nd	Tc01g003050	AT2G18280	-0.63	Tubby-like F-box protein 5
2nd	Tc09g032990	AT2G28550	-0.63	Ethylene-responsive transcription factor RAP2-7
2nd	Tc01g030640	AT2G47590	-0.65	Blue-light photoreceptor PHR2
1st	Tc09g009310	AT1G65660.1	-0.68	Hypothetical protein
2nd	Tc04g022630	AT5G35630	-0.69	Glutamine synthetase leaf isozyme, chloroplastic

2nd	Tc03g006490	AT1G19000	-0.71	Transcription factor Myb1
2nd	Tc10g002590	AT3G55610	-0.71	Delta-1-pyrroline-5-carboxylate synthase
2nd	Tc05g016260	AT5G36170	-0.72	Putative Peptide chain release factor 2
2nd	Tc01g001360	AT5G66120	-0.73	3-dehydroquinate synthase
2nd	Tc01g004960	AT4G37000	-0.73	Red chlorophyll catabolite reductase, chloroplastic
2nd	Tc04g019390	AT2G31380	-0.74	Salt tolerance-like protein
2nd	Tc00g054320	AT2G27470	-0.78	Predicted protein
2nd	Tc03g026520	AT1G63000	-0.78	Probable rhamnose biosynthetic enzyme 1
2nd	Tc02g034100	AT2G02710	-0.79	Protein TWIN LOV 1
2nd	Tc01g025670	AT1G04020	-0.83	Brca1 associated ring domain, putative
2nd	Tc06g019200	AT4G04640	-0.84	ATP synthase gamma chain, chloroplastic
2nd	Tc01g017730	AT2G32950	-0.85	E3 ubiquitin-protein ligase COP1
1st and 2nd	Tc05g002470	AT5G01410.1	-0.9	Probable pyridoxal biosynthesis protein PDX1
2nd	Tc01g038370	AT4G00050	-0.9	Transcription factor UNE10
2nd	Tc01g037800	AT1G02000	-0.91	UDP-glucuronate 4-epimerase 3
1st and 2nd	Tc03g023580	AT2G35390.2	-0.97	Ribose-phosphate pyrophosphokinase 1
2nd	Tc01g037410	AT2G45440	-0.99	Dihydrodipicolinate synthase 2, chloroplastic
1st	Tc03g022890	AT1G64660.1	-1	Putative Methionine gamma-lyase
2nd	Tc02g003980	AT5G22800	-1.04	Alanyl-tRNA synthetase
1st and 2nd	Tc09g004830	AT4G33010.1	-1.07	Glycine dehydrogenase [decarboxylating], mitochondrial
2nd	Tc08g000970	AT2G26540	-1.07	Uroporphyrinogen-III synthase, chloroplastic
2nd	Tc04g020470	AT4G13930	-1.11	Serine hydroxymethyltransferase 1
2nd	Tc07g007410	AT5G54470	-1.12	Transcription factor, putative
2nd	Tc02g003500	AT4G35270	-1.15	Putative uncharacterized protein
2nd	Tc03g025170	AT4G00150	-1.17	Putative uncharacterized protein
2nd	Tc05g031470	AT1G09340	-1.17	Uncharacterized protein At1g09340, chloroplastic
1st and 2nd	Tc03g017540	AT5G24120.1	-1.18	RNA polymerase sigma factor rpoD, putative
2nd	Tc08g008620	AT2G22430	-1.23	Putative Homeobox-leucine zipper protein ATHB-16
2nd	Tc01g002070	AT4G38960	-1.28	Putative uncharacterized protein
2nd	Tc09g029070	AT1G08200	-1.29	Putative Bifunctional polymyxin resistance protein arnA
2nd	Tc02g006400	AT4G38970	-1.3	Probable fructose-bisphosphate aldolase 1, chloroplastic
2nd	Tc04g000940	AT5G14760	-1.3	Putative L-aspartate oxidase 1
2nd	Tc03g019660	AT5G52100	-1.33	Putative uncharacterized protein
2nd	Tc03g016190	AT5G51110	-1.36	Putative pterin-4-alpha-carbinolamine dehydratase
2nd	Tc01g033120	AT3G62030	-1.47	Peptidyl-prolyl cis-trans isomerase CYP20-3, chloroplastic
2nd	Tc01g033910	AT4G00870	-1.53	Putative Transcription factor MYC2
2nd	Tc05g025090	AT1G08550	-1.68	Violaxanthin de-epoxidase, chloroplastic
2nd	Tc01g040660	AT2G41900	-1.75	Zinc finger CCCH domain-containing protein 30
2nd	Tc00g064020	AT1G70000	-1.81	Putative uncharacterized protein
2nd	Tc05g028380	AT4G32890	-1.95	Putative GATA transcription factor 9
2nd	Tc05g005790	AT3G09600	-2.39	Putative Myb-like protein G

2nd	Tc02g006880	AT4G39250	-2.52	Predicted protein
1st and 2nd	Tc02g014240	AT3G21890.1	-2.88	Predicted protein
1st	Tc01g033560	AT1G01060.3	-3.01	Putative Protein LHY
2nd	Tc04g024360	AT4G14690	-3.21	Early light-induced protein, chloroplastic

Supplementary Table 10. ***Theobroma cacao*** **genes differentially expressed in third microarray experiment (Time course).**

Oligo ID (unigene)	Tc Identifier	At Identifier	Fold change T3/T0	Fold change T7/T0	Fold change T14/T0
LITE_CONTIG_1906	Tc00g017490	AT1G52360	1.152 down	1.016 up	1.235 up
KBA4YG21FM1_	Tc00g020030	0	2.267 down	1.032 up	1.314 up
LITE_CONTIG_1719	Tc00g024070	0	1.179 down	2.248 down	2.971 down
KAS2YM21FM1_hypothetical	Tc00g029100	AT3G13060	1.000 down	1.844 up	2.550 up
KAV15YJ03FM1_gi_9757817_dbj_BAB08335_1_	Tc00g035510	AT2G25340	1.381 down	1.016 up	1.142 up
KAP7YL12FM1_gi_105830184_gb_ABF74716_1_	Tc00g045850	AT2G41980	1.800 down	1.023 down	1.554 up
LITE_CONTIG_622	Tc00g047150	AT1G12910	1.152 up	1.838 up	2.033 up
KAV6YH06FM1_Ubiquitin	Tc00g048850	AT5G06600	1.067 up	1.746 up	2.666 up
LITE_CONTIG_3001	Tc00g051020	AT1G43700	1.093 down	1.665 up	2.127 up
LKBE6YL09FM1_	Tc00g051020	0	1.020 up	2.603 up	3.208 up
KAA10YA06FM1_Strong	Tc00g051910	AT1G60070	1.107 down	1.449 up	1.675 up
LITE_CONTIG_3597	Tc00g063560	0	1.134 down	2.624 down	3.675 down
KCAE7YB21FM1_hypothetical	Tc00g085970	AT1G14920	1.416 down	1.975 up	2.267 up
LITE_CONTIG_6101	Tc00g087300	AT5G56670	1.227 up	1.778 up	1.680 up
LITE_CONTIG_6486	Tc00g087460	0	1.051 down	2.264 down	2.753 down
KAS2YJ15FM1_Fas_associated	Tc00g092190	AT4G10790	1.462 down	1.112 up	1.385 up
LITE_CONTIG_4140	Tc01g005290	AT4G34050	1.763 up	2.012 down	3.598 down
LITE_CONTIG_5585	Tc01g006660	AT1G07360	1.183 down	1.638 up	1.665 up
LITE_CONTIG_6631	Tc01g009580	AT4G39680	1.015 up	1.445 up	1.483 up
KAQ9YL01FM1_leucine_rich	Tc01g009920	AT5G10020	1.567 down	1.804 up	2.361 up
KAA12YH23FM1_gi_125562971_gb_EAZ08351_1_	Tc01g019220	AT3G07660	1.132 down	3.580 down	4.437 down
LITE_CONTIG_3415	Tc01g021920	0	1.058 up	1.604 up	1.679 up
KCL2YG24FM1_hypothetical	Tc01g022530	0	1.032 down	1.669 up	1.778 up
LITE_CONTIG_6201	Tc01g025670	AT1G04020	1.273 down	1.477 down	2.221 down
KBB13YA22FM1_gi_110735118_gb_ABG89129_1_	Tc01g029320	AT3G63000	1.292 down	2.025 up	2.475 up
172868417_m00099	Tc01g036690	AT4G00730	1.034 up	2.357 up	3.435 up
KBB10YA01FM1_gi_21281074_gb_AAM45055_1_	Tc01g037860	AT2G45260	1.154 down	1.367 up	1.399 up
361268416_m06790	Tc01g037950	AT3G60690	1.032 up	1.655 up	2.195 up
LITE_CONTIG_2414	Tc01g040740	AT3G08580	1.122 down	1.593 up	2.020 up
LITE_CONTIG_2526	Tc02g000220	AT4G20110	1.138 up	1.851 up	2.662 up
KAP15YH02FM1_hypothetical	Tc02g001520	AT1G33980	1.108 down	1.790 up	2.252 up
LITE_CONTIG_4326	Tc02g004750	AT2G20930	1.078 up	1.586 up	1.664 up
KAC3YE20FM1_gi_110738152_dbj_BAF01007_1_	Tc02g005290	AT3G16620	1.099 up	2.481 up	2.710 up
LITE_CONTIG_4699	Tc02g005650	AT4G34840	1.196 down	1.919 down	1.657 down
LITE_CONTIG_2779	Tc02g008990	AT4G34050	1.225 up	2.014 down	2.902 down
KAV15YD13FM1_hypothetical	Tc02g009070	AT4G34100	1.167 up	1.717 up	2.057 up
KAS1YC12_large	Tc02g009720	0	1.155 up	1.389 down	1.428 down
LITE_CONTIG_4780	Tc02g011880	AT5G64130	1.064 up	1.504 down	1.593 down
CL283Contig1_aminoacylase,	Tc02g012580	AT4G38220	1.088 down	1.701 up	1.886 up
1308_68418_m02752	Tc02g012930	AT5G23450	1.010 down	1.649 up	1.869 up

LITE_CONTIG_3783	Tc02g014332	0	1.125 down	2.958 down	3.675 down
KCL4YJ02FM1_hypothetical	Tc02g017370	AT5G08690	1.074 down	1.646 up	1.673 up
KAT3YK06FM1_hypothetical	Tc02g025290	AT1G24560	1.547 down	1.523 up	1.590 up
LITE_CONTIG_1626	Tc02g026040	AT1G67700	1.122 down	1.925 down	2.823 down
KBB14YL09FM1_NADH	Tc02g026650	AT5G08530	1.218 down	1.413 up	1.751 up
KAP24YN02FM1_gi_22136836_gb_AAM91762_1_	Tc02g028410	AT2G01600	1.038 down	1.275 up	1.489 up
KBB12YH21FM1_gi_51971597_dbj_BAD44463_1_	Tc02g031380	AT1G26300	1.017 down	1.361 up	1.585 up
2004_68416_m00180	Tc02g032000	AT3G02130	1.393 down	1.026 up	1.690 up
KAV11YK16FM1_gi_8778406_gb_AAF79414_1_AC068197_24	Tc02g032380	AT1G13900	1.260 up	2.188 up	3.645 up
LITE_CONTIG_5947	Tc02g034730	AT1G26110	1.060 down	1.483 up	1.944 up
KCAA9YI24FM1_gi_26452241_dbj_BAC43208_1_	Tc03g000230	0	1.078 up	1.133 down	1.496 down
LITE_CONTIG_3444	Tc03g002220	AT4G24690	1.146 down	1.877 up	1.997 up
KAC2YL13FM1_	Tc03g002950	0	1.203 up	1.533 up	1.494 up
LITE_CONTIG_763	Tc03g014080	AT3G48420	1.093 down	1.885 down	2.459 down
LITE_CONTIG_1291	Tc03g016980	AT5G51300	1.325 down	1.611 up	1.470 up
KBA3YO22FM1_putative	Tc03g019770	AT1G79940	1.121 down	1.083 up	1.455 up
LITE_CONTIG_3405	Tc03g020440	AT4G20850	1.300 down	1.278 up	1.308 up
LITE_CONTIG_5625	Tc03g022770	AT5G42090	1.154 down	1.158 up	1.717 up
LITE_CONTIG_1902	Tc03g024770	AT5G47430	1.007 down	1.309 up	1.550 up
LITE_CONTIG_3755	Tc03g026500	AT1G62990	1.173 up	1.798 up	2.224 up
118968418_m05006	Tc03g027230	AT5G41190	1.030 up	2.187 up	2.060 up
KAT3YC16FM1_	Tc03g029560	0	1.159 up	2.213 up	3.168 up
KBA4YA16FM1_gi_25083846_gb_AAN72126_1_	Tc03g029720	AT1G32340	1.101 down	1.368 up	1.530 up
KAA10YO11FM1_hypothetical	Tc03g029940	AT1G32230	1.061 up	1.248 up	1.666 up
1486_68418_m01713	Tc04g001160	AT5G14610	1.027 up	1.855 up	2.064 up
LITE_CONTIG_3820	Tc04g002820	AT3G27240	1.129 up	1.458 up	1.508 up
LITE_CONTIG_2359	Tc04g011870	AT3G02360	1.016 down	1.111 up	1.471 up
LKBE5YG15FM1_GAT	Tc04g016790	AT2G38410	1.365 down	2.732 up	3.141 up
LITE_CONTIG_609	Tc04g017420	0	1.134 up	1.351 down	1.247 down
322268415_m05361	Tc04g018830	0	1.459 down	1.761 up	2.884 up
1426_68416_m02975	Tc04g020060	AT1G05520	1.214 down	1.316 up	1.724 up
LITE_CONTIG_890	Tc04g021340	AT3G24160	1.077 down	1.335 up	1.662 up
LITE_CONTIG_3484	Tc04g022120	AT3G24010	1.131 up	1.384 down	1.065 down
LITE_CONTIG_3573	Tc04g023730	AT1G05380	1.006 down	1.122 up	1.481 up
LITE_CONTIG_1418	Tc04g023840	AT2G32090	1.202 down	1.885 down	1.373 down
169868417_m02235	Tc04g024790	AT4G14500	1.028 down	3.003 up	2.582 up
KAV3YB19FM1_hypothetical	Tc04g026080	AT4G14300	1.065 up	1.602 up	1.859 up
KAA15YF17FM1_gi_5931637_emb_CAB56575_1_	Tc04g027170	AT5G18830	1.088 down	1.296 up	1.490 up
LITE_CONTIG_1943	Tc04g030610	AT1G09520	1.116 down	2.352 up	2.571 up
LITE_CONTIG_6206	Tc05g000050	AT2G43970	1.115 down	2.288 up	2.325 up
KAA5YD18FM1_gi_56806666_dbj_BAD83567_1_	Tc05g000780	ATMG00030	1.067 up	4.523 down	4.052 down
LCL1Contig2_Sabia	Tc05g000780	0	1.393 down	1.342 up	1.635 up
CL115Contig1_hypothetical	Tc05g003160	AT2G38000	1.230 down	1.339 up	1.829 up
KBB16YN05FM1_gi_110743100_dbj_BAE99442_1_	Tc05g005250	AT5G02580	1.106 up	4.770 up	7.419 up
LITE_CONTIG_6247	Tc05g008730	AT3G52760	1.120 down	1.457 up	1.477 up

KBB13YC13FM1_hypothetical	Tc05g009290	AT5G22400	1.142 up	1.580 up	1.602 up
KAS7YL07FM1_gi_23198400_gb_AAN15727_1_	Tc05g014010	AT2G40950	1.411 down	1.198 up	1.457 up
CL303Contig1_hypothetical	Tc05g030270	AT3G05545	1.142 down	1.263 up	1.526 up
LITE_CONTIG_4112	Tc05g031360	AT1G09270	1.128 up	1.487 up	1.859 up
KAQ9YK05FM1_hypothetical	Tc06g000870	AT4G20440	1.031 down	1.843 up	1.938 up
LITE_CONTIG_2144	Tc06g001640	AT1G52570	1.170 down	1.094 up	1.444 up
LITE_CONTIG_1702	Tc06g001640	AT3G15730	2.147 down	1.187 up	1.542 up
KAV14YP03FM1_inorganic	Tc06g003340	AT1G15690	1.220 down	2.154 up	2.412 up
LITE_CONTIG_2720	Tc06g004850	0	1.027 down	1.358 up	1.375 up
218168416_m00051	Tc06g007720	AT3G01340	1.106 down	1.200 up	1.319 up
KCAK2YD19FM1_hypothetical	Tc06g008930	AT5G56890	1.013 up	1.141 down	1.893 down
LITE_CONTIG_1792	Tc06g009960	AT5G20510	1.088 up	1.103 down	1.527 down
LITE_CONTIG_341	Tc06g012360	0	1.062 up	1.572 up	1.937 up
LITE_CONTIG_4919	Tc06g014120	AT4G18710	1.029 up	2.094 up	2.926 up
CL149Contig1_eukaryotic	Tc06g015690	AT5G44320	1.497 down	2.355 up	2.998 up
LITE_CONTIG_1705	Tc07g000170	AT4G26450	1.178 down	1.251 up	1.583 up
KBB10YM23FM1_gi_20334818_gb_AAM16165_1_	Tc07g002500	AT4G26750	1.244 up	1.890 up	2.081 up
KAQ9YO04FM1_unnamed	Tc07g008580	AT3G15180	1.212 down	1.260 up	2.024 up
KBB2YJ24FM1_hypothetical	Tc08g001190	AT1G06870	1.681 down	1.770 up	2.480 up
LITE_CONTIG_6495	Tc08g004720	AT4G35230	1.151 down	1.224 up	1.708 up
LITE_CONTIG_5592	Tc08g007870	AT3G50050	1.005 down	1.582 up	1.767 up
KBB3YP12FM1_	Tc08g008580	0	1.105 up	2.484 up	3.204 up
KAA11YE20FM1_gi_13876508_gb_AAK43484_1_AC084807_9	Tc08g011660	AT1G44180	1.851 down	1.281 up	1.925 up
KAA11YO10FM1_gi_140062975_gb_ABO82144_1_	Tc08g015730	AT1G73720	1.515 down	1.123 up	1.138 up
LITE_CONTIG_5594	Tc09g002430	AT5G25400	1.233 down	1.842 up	3.096 up
KAQ11YJ11FM1_gi_22327070_ref_NP_680223_1_	Tc09g003670	AT5G25757	1.057 up	1.929 up	2.361 up
KAP7YK21FM1_gi_7269883_emb_CAB79742_1_	Tc09g007370	AT4G29840	1.518 down	1.753 up	2.068 up
LITE_CONTIG_4051	Tc09g007710	AT2G19480	1.016 up	1.546 up	1.666 up
KCL2YH24FM1_hypothetical	Tc09g008570	AT4G26270	1.218 down	1.236 up	1.309 up
KAQ10YO22FM1_	Tc09g008570	0	1.525 down	1.773 up	1.895 up
KCL5YO15FM1_gi_17065608_gb_AAL33784_1_	Tc09g008900	AT4G29120	1.025 down	2.365 up	2.555 up
LITE_CONTIG_4412	Tc09g009540	AT4G29010	1.016 down	1.669 up	1.997 up
LITE_CONTIG_1749	Tc09g010120	AT4G13350	1.365 down	1.735 up	1.921 up
LITE_CONTIG_1333	Tc09g010660	AT3G15610	1.410 down	2.044 up	2.373 up
LITE_CONTIG_1045	Tc09g012390	AT2G11890	1.121 down	1.174 up	1.337 up
KAV6YI09FM1_gi_110737119_dbj_BAF00511_1_	Tc09g012410	AT5G20350	1.393 down	1.456 up	2.042 up
KBF7YM19FM1_gi_7270166_emb_CAB79979_1_	Tc09g012940	AT4G32620	1.060 up	1.425 up	1.789 up
KAP13YK03FM1_Heat	Tc09g015680	AT5G57710	1.437 down	1.393 up	1.674 up
LITE_CONTIG_4310	Tc09g018410	AT3G13460	1.142 up	1.591 up	1.791 up
LITE_CONTIG_4006	Tc09g022240	AT1G18450	1.091 down	1.819 up	1.856 up
KAP13YI10FM1_	Tc09g025960	0	1.084 down	2.048 up	1.870 up
KAQ5YN11FM1_hypothet	Tc09g029380	AT2G27960	1.126 down	2.139 down	3.073 down

ical					
KAA15YL19FM1_gi_395 45872_gb_AAR27999_1_	Tc09g031190	AT1G07480	1.156 down	1.312 up	1.879 up
KAP3YI06FM1_RNA_bin ding	Tc09g033000	AT5G60170	1.019 down	1.543 up	2.013 up
LITE_CONTIG_3523	Tc09g033150	AT3G20770	1.161 down	1.768 up	2.245 up
LKBE9YG15FM1_transcri ption	Tc09g033150	AT3G20770	1.284 down	1.755 up	2.441 up
LITE_CONTIG_1475	Tc10g000070	AT5G58230	1.013 up	1.524 up	1.770 up
LITE_CONTIG_234	Tc10g001880	AT3G09200	1.149 up	2.030 up	2.448 up
LITE_CONTIG_2460	Tc10g002050	AT2G39980	1.031 up	1.401 down	1.691 down
KAA15YK21FM1_serine/t hreonine	Tc10g002440	AT2G39840	1.132 up	1.699 up	2.066 up
LITE_CONTIG_1548	Tc10g002950	AT2G44920	1.990 up	1.336 up	1.605 down
LITE_CONTIG_5046	Tc10g004430	AT1G28060	1.076 down	1.836 up	2.135 up
LITE_CONTIG_6287	Tc10g005640	AT2G01720	1.035 up	1.713 up	2.122 up
gi_148726971_gb_EH0577 21_1_EH057721	0	0	1.076 down	1.716 down	1.986 down
KCAE7YH06FM1_	0	0	1.159 down	2.930 down	2.856 down
LITE_CONTIG_6386	0	0	1.850 down	4.567 down	4.397 down
LITE_CONTIG_505	0	0	1.423 down	3.807 up	4.670 up
